# Dual-ligand PROTACS mediate superior target protein degradation *in vitro* and therapeutic efficacy *in vivo*†

**DOI:** 10.1039/d4sc03555k

**Published:** 2024-10-02

**Authors:** Yong Chen, Zihan Xia, Ujjwal Suwal, Pekka Rappu, Jyrki Heino, Olivier De Wever, Bruno G. De Geest

**Affiliations:** a Department of Pharmaceutics, Ghent University 9000 Ghent Belgium br.degeest@ugent.be; b Laboratory of Experimental Cancer Research, Department of Human Structure and Repair, Ghent University 9000 Ghent Belgium olivier.dewever@ugent.be; c Cancer Research Institute Ghent 9000 Ghent Belgium; d Department of Life Technologies, InFLAMES Flagship, University of Turku 20520 Turku Finland

## Abstract

Proteolysis targeting chimeras (PROTACs) are revolutionizing the drug development landscape due to their unique ability to selectively degrade disease-associated proteins. Conventional PROTACs are bivalent entities that induce ubiquitination and subsequent proteolysis of a chosen protein of interest (POI) by forming a ternary complex with an E3 ligase. We hypothesized that dual-ligand PROTACs, featuring two copies each of a POI ligand and an E3 ligase ligand, would facilitate the formation of high-avidity, long-lived ternary complexes inside cells, thereby increasing POI degradation potency. To this end, we developed a convergent synthesis route, using l-aspartic acid as a building block for homodimer synthesis, followed by copper-catalyzed azide–alkyne cycloaddition (CuAAC) to conjugate both dimers through a flexible linker. Dual-ligand PROTACs achieved up to a tenfold increase in degradation efficiency and a hundredfold increase in cytotoxicity *in vitro* across various cancer cell lines compared to their single-ligand counterparts. Furthermore, dual-ligand PROTACs sustain prolonged protein degradation, up to 60 hours after pulsing and washout. *In vivo*, in a mouse tumor model, the superior therapeutic activity of dual ligand PROTACs was observed.

## Abstract 2

Dual-ligand PROTACs which comprise of two copies of each E3 ligase ligand and targeted protein ligand display superior activity compared to conventional single-ligand PROTACs. The higher activity of dual-ligand PROTACs is enabled by the stabilized and long-lived ternary complex formation.

## Graphical abstract

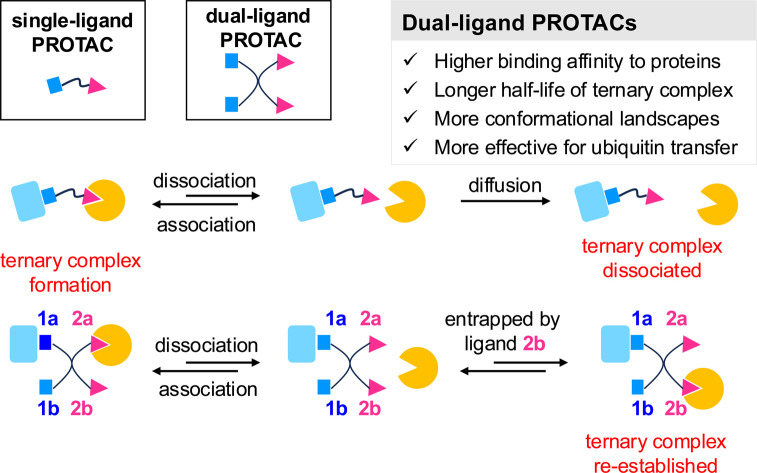

## Introduction

Proteolysis-targeting chimeras (PROTACs) are revolutionizing the field of drug development by enabling the degradation of disease-related proteins, rather than merely inhibiting them.^1^ These bivalent small molecules consist of a ligand for the protein of interest (POI) connected to a ubiquitin E3 ligase ligand *via* a linker. Through their unique mechanism of action, PROTACs mediate the formation of ternary complexes involving the POI, PROTAC, and E3 ligase. This complex formation brings the POI and E3 ligase into proximity, thereby instigating polyubiquitination of the POI which is subsequently flagged for proteasomal degradation.^2^ PROTACs have several advantages over traditional small molecule inhibitors. They demonstrate remarkable selectivity among homologous proteins and exhibit enhanced potency due to their catalytic mode of action, as a single PROTAC molecule can mediate degradation of multiple POIs.^3–7^ Unlike inhibitors, which often interact with the active site of a protein, PROTACs can induce favorable target-ligase protein–protein interactions (PPIs), and result in the eventual degradation of POI, including target proteins that have thus far been considered undruggable.^8,9^ The current trajectories for PROTAC development heavily rely on empirical methods, necessitating extensive optimization *via* combinatorial testing of various ligands and linker designs.^10^ However, even minor changes to the chemical structures of the ligands and linkers can significantly influence the PROTACas' degradation potency and specificity.^11–13^

**Fig. 1:**
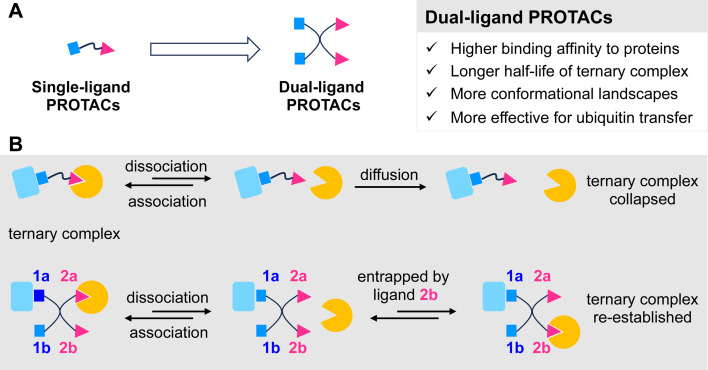
Rationale for the design of dual-ligand PROTACs. (A) Conceptual representation of conventional single-ligand PROTACs and dual-ligand PROTACs. (B) The fate of ternary complex mediated by single-ligand PROTACs and dual-ligand PROTACs. According to our hypothesis, dual-ligand PROTACs can increase both the half-life and conformational landscape of the ternary complex. In the context of dual-ligand PROTACs-mediated ternary complex dynamic equilibrium, a protein that dissociates from ligand 2a possesses the potential to be promptly re-engaged by an adjacent identical ligand 2b. This interaction facilitates the formation of a novel ternary complex or enables the re-establishment of the initial ternary complex configuration. Conversely, in the scenario of single-ligand PROTACs systems, the disengagement of the protein from its ligand typically results in its subsequent diffusion away from the complex, resulting in the collapse of the original ternary complex structure.

Although relatively weak binding ligands can be used for PROTAC design,^6,14^ a threshold binding affinity of the ligand must be achieved for long-lived ternary complex formation and efficient PROTAC-mediated POI degradation.^15–18^ Here, we hypothesized that dual-ligand PROTACs – comprising two copies each of the POI and E3 ligands (Fig. 1A) – would further promote high-avidity ternary complex formation by clustered multivalency,^19,20^ which increases the local ligand concentration (Fig. 1B). A more stable ternary complex could extend the intracellular residence time of the PROTAC molecules and reduce efflux. In addition, dual-ligand PROTACs could provide a 2 × 2 conformational landscape for the ternary complex, in contrast to the 1 × 1 conformation of classical PROTACs (Fig. 1B). This expanded conformational space could potentially increase the likelihood of productive ternary complex formation and subsequent transfer of ubiquitin motifs to the POI, thereby marking it for proteasomal degradation.

While the potential advantages associated with dual-ligand PROTACs are exciting, they also present potential drawbacks. Firstly, the inclusion of two E3 ligase ligands in dual-ligand PROTACs could lead to unwanted homo-degradation, resulting in the loss of functional E3 ligase and hindering target protein degradation.^21,22^ Secondly, due to their high molecular weight, dual-ligand PROTACs would significantly deviate from Lipinski's rule-of-five for small molecule drug design, reducing cellular uptake and altering pharmacokinetic properties compared to conventional small molecule drugs.^23^

In this paper, we counter the aforementioned concerns and present evidence that dual-ligand PROTACs featuring two copies of a ligand targeting a POI for degradation and two copies of either Cereblon (CRBN) or von Hippel–Lindau (VHL) ligand, respectively, as the E3 ligase ligand, can degrade that target POI much more potently *in vitro*, and show increased cytotoxic activity on 2D and 3D cell cultures. These findings could be translated *in vivo*, showing an enhanced therapeutic activity in a tumor-bearing mouse model, in comparison to conventional single-ligand PROTACs. For proof-of-concept we focus in this work on the bromodomain extra-terminal (BET) family of proteins, which influence gene expression and contribute to the development of cancer, as the model POI. Notably, PROTACs consisting of two copies of a ligand for the BET family proteins and a single VHL E3 ligase ligand have been recently reported by the Ciulli group, demonstrating substantial degradation of BRD proteins *in vitro*, attributed to both avidity and cooperativity effects.^18^ However, to the best of our knowledge, dual-ligand PROTACs containing multiple copies of each ligand have not yet been reported.

## Results

### Synthesis of dual-ligand PROTACs

**Fig. 2:**
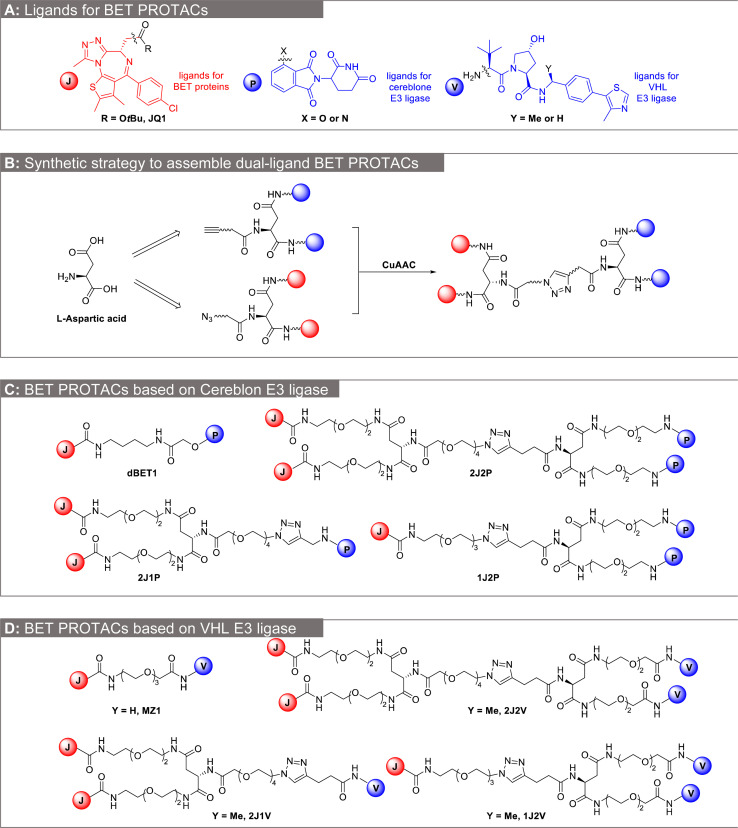
Convergent synthesis of dual ligand BET PROTACs. (A) Chemical structure of the ligands used for the design of BET PROTACs. (B) General synthetic strategy to assemble the dual-ligand BET PROTACs. (C) Chemical structure of BET PROTACs based on pomalidomide for recruitment of the CRBN E3 ligase: dBET1 (single-ligand PROTACs), trivalent PROTACs (2J1P, 1J2P) and dual-ligand PROTACs (2J2P) (D) chemical structure of BET PROTACs based on VHL-ligand for recruitment of the VLH E3 ligase: MZ1 (single-ligand PROTACs), trivalent PROTACs (2J1V, 1J2V) and dual-ligand PROTACs (2J2V).

As a proof of concept, we selected a well-established PROTAC system utilizing JQ1 (J, represented in red in Fig. 2A) as a ligand for the BET family of proteins.^24^ The pomalidomide derivative (P, represented in blue in Fig. 2A) was selected as a ligand targeting the E3 ubiquitin ligase Cereblon (CRBN),^25^ and the tripeptide von Hippel–Lindau (VHL) ligand (V, represented in blue in Fig. 2A) was selected as a ligand for the von Hippel–Lindau E3 ubiquitin ligase.^26–28^JQ1 is a potent inhibitor of the BET family of proteins, specifically BRD2, BRD3, and BRD4, which are actively explored therapeutic targets in cancer through inhibition or degradation.^29^ The PROTACs, dBET1 (Fig. 2B) and MZ1 (Fig. 2C), incorporating JQ1 and P or V ligands (Fig. 2A), respectively, have undergone extensive optimization and have been widely employed as molecular probes for investigating various mechanistic aspects of PROTACs.^4,5,30^ The envisioned dual-ligand PROTACs 2J2P and 2J2V were shown in Fig. 2B and C. To compare the 2 × 2 combination with 1 × 2 or 2 × 1 combinations, we also synthesized the trivalent PROTACs of 1J2P, 2J1P, 1J2V and 2J1V (Fig. 2B and C). Dual-ligand PROTACs were synthesized through a convergent approach in which each half of the dual-ligand compounds was synthesized separately, followed by their subsequent conjugation (Fig. 2D). For the latter purpose, we utilized l-aspartic acid as the molecular cornerstone. Its two carboxylic acid groups were simultaneously derivatized with two identical ligand copies, while the remaining amino group was further modified with an azide or alkyne moiety, respectively. The obtained two homodimers were then conjugated by copper catalyzed azide alkyne cycloaddition (CuAAC) reaction, to form dual-ligand PROTACs (Fig. 2D).^31^ To mitigate the likelihood of homo-degradation of E3 ligase itself, we selected a relatively long polyethylene glycol (PEG) linker, consisting of ≥3 ethylene glycol repeating units, between each E3 ligase ligand. Furthermore, another long (≥3) oligo(ethylene glycol) spacer was inserted between the E3 ligase ligand and the POI ligand moieties. We hypothesized that a long and flexible linker would minimize both steric hindrance and unfavorable interactions at the interface of the target protein and the E3 ligase.^32^

**Fig. 3:**
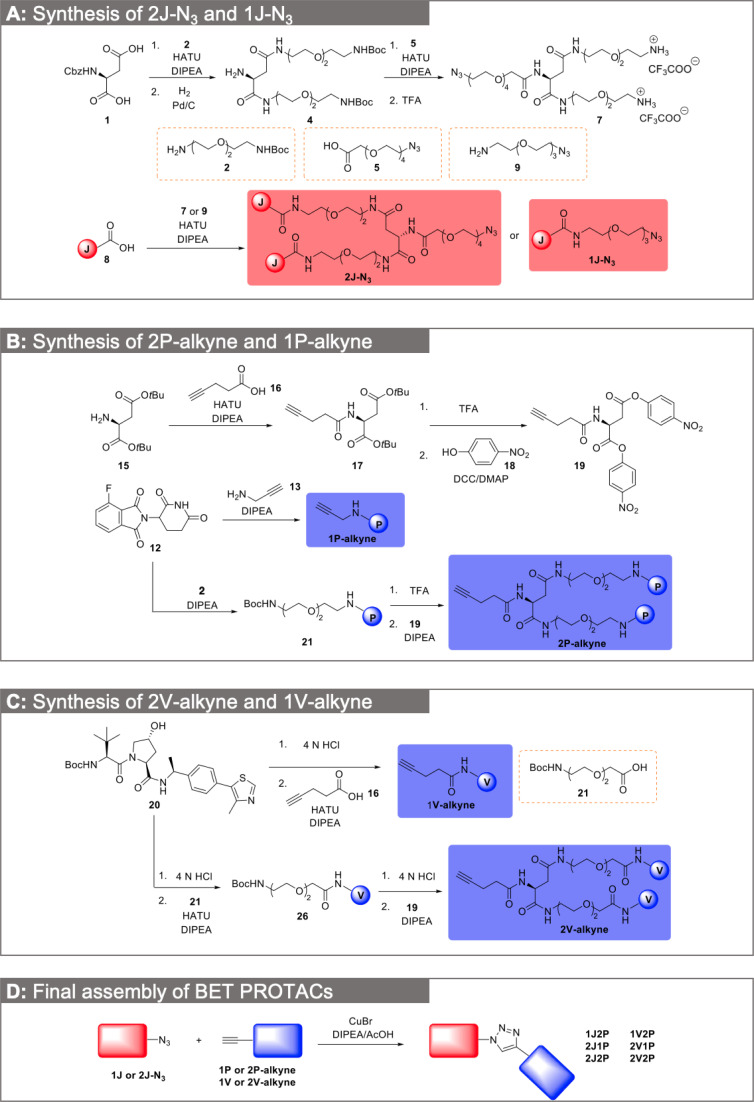
Detailed synthesis of dual-ligand and trivalent BET PROTACs. (A) Synthesis of 2J-N_3_ and 1J-N_3_. (B) Synthesis of 2P-alkyne and 1P-alkyne. (C) Synthesis of 2V-alkyne and 1V-alkyne. (D) Final PROTAC assembly by CuAAC conjugation.

The synthesis commenced with the dimerization of the JQ1 acid 8 (Fig. 3A). *N*-Cbz-l-aspartic acid 1 first reacted with *t*-Boc-*N*-amido-PEG2-amine 2 to deliver a bis-amide, which was subsequently submitted to hydrogenolysis to afford amine 4. Amine 4 was then coupled with azido-PEG4-acid 5, followed by trifluoroacetic acid (TFA) deprotection of Boc group to deliver the key bisamine 7. The amidation between JQ1 acid 8 and bisamine 7 readily provided product 2J-N_3_. Meanwhile, the 1J-N_3_ was also readily obtained by coupling acid 8 with azido amine 9 through HATU-mediated amide bond formation. The homodimerization of the CRBN and VHL ligands is shown in Fig. 3B and C. The key intermediate 19 with bis *para*-nitrophenol activated esters was an easy-to-handle solid and could be readily obtained from l-aspartic acid di-*tert*-butyl ester hydrochloride 15*via* a 3-step transformation sequence (Fig. 3B). The pomalidomide derivative 21 or VHL ligand derivative 26, after Boc deprotection, reacted readily with compound 19 to deliver the bisamide product 2P-alkyne or 2V-alkyne in high yield. Compared to the one-step amide coupling reaction between an amine and a corresponding aspartic acid derivative, aminolysis of compound 19 proved to be superior in both reaction reproducibility and yield. In parallel, 1P-alkyne and 1V-alkyne were efficiently obtained from compounds 12 and 20, respectively. With both dimeric POI and E3 ligase ligands at hand, the final PROTACs were readily assembled by CuAAC click reaction (Fig. 3D).^33^ It is noteworthy that by separately assembling each half of the PROTACs, our synthetic route is highly modular, convergent and amenable to easy derivatization. This is illustrated by applying this route for the straightforward synthesis of trivalent PROTACs containing two E3 ligases and a single POI moiety or *vice versa*, which will serve for head-to-head comparison with dual ligand PROTACs in further experiments.

### Dual-ligand PROTACs induce potent degradation of BET family proteins *in vitro*

**Fig. 4:**
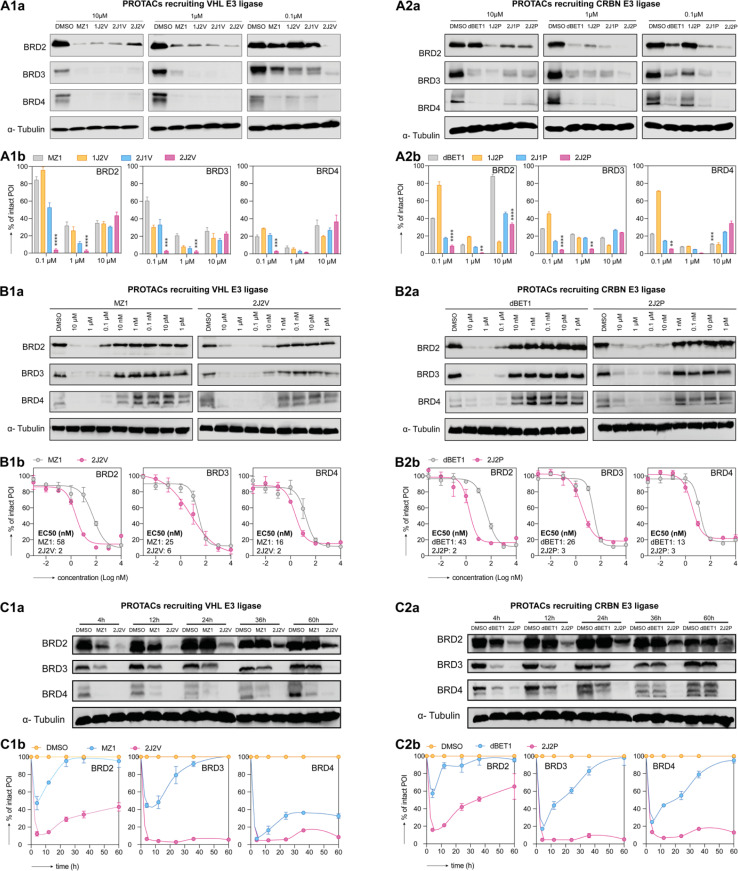
Dual-ligand PROTACs induce robust and prolonged target protein degradation *in vitro*. HEK293 cells were treated with PROTACs recruiting the VHL E3 ligase (panels 1) and the CRBN E3 ligase (panels 2) for 4 h. Subsequently, cells were either lysed (A and B) or washed and cultured in the fresh medium until the desired time point of lysis (C). BRD2, BRD3 and BRD4 protein levels were measured by western blot (panels a for a representative image) and quantification was done by optical density integration of the protein bands (panels b). (A) BET protein degradation was screened for all synthesized PROTACs and commercial single-ligand PROTACs at a concentration of 0.1, 1, 10 μM, respectively with DMSO as the control vehicle. Experiments were repeated as independent replicates. Statistical analysis by one-way ANOVA. (**: *p* < 0.01, ***: *p* < 0.001, ****: *p* < 0.0001) (B) BET protein degradation by single- and dual-ligand PROTACs was tested over a 1 pM–10 μM concentration range (vehicle-only control contained DMSO). EC_50_ values were calculated by curve fitting of three independent experiments. (C) BET protein degradation by single- and dual-ligand PROTACs over time after wash-out. Experiments were performed as three independent replicates.

We conducted western blot analysis to assess the potential of our synthesized PROTACs to degrade BRD2, BRD3, and BRD4 proteins as target POIs in HEK293 human embryonic kidney cells. We compared the efficacy of these PROTACs with the single-ligand PROTACs, dBET1 and MZ1, at concentrations of 0.1 μM, 1 μM, and 10 μM. Our findings (Fig. 4A) revealed that at the lower concentrations (*i.e.*, 0.1 μM and 1 μM), the dual-ligand PROTACs 2J2P and 2J2V exhibited superior degradation efficiency compared to all single- and trivalent PROTACs across all three BRD proteins. However, at a higher concentration of 10 μM, the dual-ligand PROTACs lost their advantage, likely due to the occurrence of the hook effect, wherein the formation of ternary complexes is hindered in favor of binary ligand–protein complex formation. The superior performance of dual-ligand PROTACs over single-ligand PROTACs at low concentrations was also observed in A549 human non-small cell lung cancer cells (see ESI Fig. S1†). The potent degradation efficiency of dual-ligand PROTACs is notable considering their large size (2J2P: *M*_W_ ∼2.4 kDa and 2J2V: *M*_W_ ∼2.8 kDa) and polarity (tPSA > 600 Å), suggesting their ability to effectively cross the cell membrane and reach their intracellular targets. A parallel artificial membrane permeability assay (PAMPA) (ESI Section 4.9†) was performed to evaluate the membrane permeability of dBET1, MZ1, 2J2P and 2J2V, demonstrating that dBET1, MZ1 and 2J2V had very limited membrane permeability, while, interestingly, 2J2P showed high membrane permeability.

To further assess the efficacy of the dual-ligand PROTACs, *i.e.*, 2P2J and 2J2V, we conducted experiments on HEK293 cells across a wider range of concentrations and compared their performance to that of their single-ligand counterparts (Fig. 4B). Our results consistently demonstrated that dual-ligand PROTACs exhibited approximately one order of magnitude higher potency than single-ligand PROTACs for all tested BRD proteins, irrespective of whether the PROTAC was pomalidomide- or VHL-ligand-based. Interestingly, while the single-ligand PROTACs displayed a more efficient degradation of BRD3 and BRD4 compared to BRD2, the dual-ligand PROTACs indiscriminately degraded all three proteins.

To mitigate the influence of the variable linker length used in the PROTACs molecules, we resynthesized single-ligand PROTACs 1J1P*, 1J1V* and trivalent PROTACs 2J1P*, 2J1V* with longer but similar linker length to that of the dual-ligand PROTACs (ESI 3.4 and 3.5†). Western blot analysis demonstrated that the newly synthesized PROTACs 1J1P* and 1J1V* significantly lost their protein degradation potency compared to the optimized PROTACs dBET1 or MZ1. While the 2J1P* and 2J1V* could still potently degrade the BRD proteins, the dual-ligand PROTACs 2J2P and 2J2V featured similar or superior protein degradation potency (ESI Fig. S2†).

To investigate the dynamics of protein degradation over time, we treated HEK293 cells with a concentration of 100 nM of PROTACs for 4 hours and subsequently replaced the culture medium with fresh medium devoid of PROTACs. Western blot analysis of BRD2, BRD3, and BRD4 protein levels at different time points revealed rapid protein degradation in all PROTAC-treated cells within the initial 4 hours period. However, following medium replacement, the protein levels in cells treated with single-ligand PROTACs quickly recovered within the first 20 hours. In contrast, cells treated with dual-ligand PROTACs exhibited either slow recovery of protein levels for BRD2 or sustained low levels for BRD3 and BRD4 for up to 60 hours post-treatment (Fig. 4C). We attribute the prolonged degradation effect to the formation of a highly stabilized intracellular ternary complex composed of BET protein, dual-ligand PROTACs, and E3 ligase. This stabilization traps the dual-ligand PROTAC molecules inside the cell and prevents their washout (ESI Fig. S3†). Such an intracellular sink effect has been documented in the literature and was recently leveraged in the design of bifunctional small molecule protein inhibitors.^34–36^ These findings demonstrate that dual-ligand PROTACs likely form a more stable and long-lived ternary complex compared to single-ligand PROTACs, exert a prolonged degradation effect, and exhibit superior performance, even at very low concentrations.

### Mechanistic aspects of dual ligand PROTACs

To confirm the role of proteasomal degradation in the action of dual-ligand PROTACs on BET target proteins, cells were treated for 4 hours with or without the proteasome inhibitor MG132. Afterwards, the medium was removed and replaced by fresh medium containing single and dual-ligand PROTACs, respectively. Western blot analysis (Fig. S4A†) revealed that MG132 blocked the degradation activity of both single and dual-ligand PROTACs, emphasizing their dependence on the proteasome as the pivotal mechanism for PROTAC-mediated protein degradation.

Homo-bifunctional compounds containing two E3 ligase ligand copies have been reported to induce degradation of the E3 ligase itself, a phenomenon referred to as ‘homo-degradation.’ To investigate whether dual-ligand PROTACs also induced E3 ligase degradation, we treated HEK293 cells with PROTACs at three different concentrations, followed by western blot analysis of the levels of CRBN and VHL E3 ligase in cell lysate. Our results revealed that 1J2P and 2J2P PROTACs did not induce any significant decrease of CRBN at all three concentrations (Fig. S5A†). This observation is consistent with literature reports suggesting that a similar linker length between pomalidomide does not induce homo-degradation of CRBN.^22^ However, 1J2V and 2J2V PROTACs did induce homo-degradation of VHL at concentrations of 1 μM and 10 μM, but not at 0.1 μM. The unaltered level of VHL at lower concentrations could be attributed to the preferential formation of a BRD/PROTACs/VHL complex over a VHL/PROTACs/VHL complex since the BRD protein can be effectively degraded at these concentrations.

To further examine these phenomena, we treated HEK293 cells with bivalent pomalidomide and VHL ligand constructs, *i.e.*, 2V-alkyne and 2P-alkyne (for structures see Fig. 3B and C), respectively, which lacked the BET protein ligand. Western blot analysis of cell lysates revealed that only 2V-alkyne could degrade VHL ligase, even at a concentration of 0.1 μM (Fig. S5B†). This suggests that the dual-ligand PROTACs 2J2V exhibits preferential degradation of the target protein over the VHL E3 ligase at lower concentrations. In contrast, ligand 2P-alkyne did not induce any degradation of CRBN at any of the tested concentrations (Fig. S5B†). These observations indicate that the free bivalent CRBN ligand alone does not possess inherent CRBN degrading activity. To exclude the possibility that the CRBN ligand of the dual-ligand PROTACs 2J2P could function as a hydrophobic tag and subsequently induce the protein destabilization and degradation, we incubated HEK293 with 2J2P in the presence or absence, respectively, of lenalidomide and monitored the BRD4 protein level (ESI Fig. S4B†). We observed that the addition of lenalidomide significantly reduced the 2J2P protein degradation efficiency, highlighting the importance of the E3 ligase engagement. These experiments collectively supported 2J2P and 2J2V are *bona fide* PROTACs.

### Dual-ligand PROTACs inhibit cancer cell growth *in vitro*

**Fig. 5:**
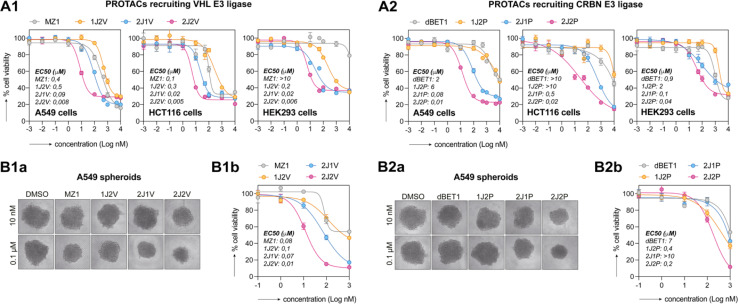
Dual-ligand PROTACs inhibit cancer cell growth in 2D and 3D *in vitro* cell cultures. (A) 2D-cultured cell lines were treated with an escalating dose of PROTACs for 48 h. Metabolic activity was assessed by CellTiter-Glo 2D assay. EC_50_ values were calculated from fitted curves. (B) A549 spheroids were treated with an escalating dose of PROTACs for 72 h. (Ba) Representative transmitted microscopy images. (Bb) Metabolic activity was assessed by CellTiter-Glo 3D assay. Both experiments were performed as three independent replicates.

Growing evidence coming from preclinical studies and clinical trials indicates the role of BET proteins in carcinogenesis and has provided the rationale for targeting BET proteins as a strategy for the development of new anticancer drugs. To confirm the greater potency of our synthesized PROTACs, we evaluated the inhibition of metabolic activity of HEK293, A549, HCT116 and SKOV-3 by CellTiter-Glo assay (Fig. 5A and S7†). Dual-ligand PROTACs exhibited significantly higher metabolic inhibition compared to both single-ligand and trivalent PROTACs, respectively, with an approximately 100-fold increase in potency. The trivalent PROTACs, 2J1P and 2J1V, also exhibited higher metabolic inhibition compared to single-ligand PROTACs, which is consistent with recent findings reported by the Ciulli group.^18^ Furthermore, we observed that the VHL ligand-based dual-ligand PROTACs 2J2V displayed greater metabolic response compared to the CRBN ligase-based dual-ligand PROTACs 2J2P, despite both PROTACs demonstrating a similar efficiency in degrading BRD proteins *in vitro* (Fig. 4C). To investigate whether the potent activity of 2J2V was merely due to enhanced inhibition of BRD proteins by dual display of the JQ1 ligand, we treated A549 cells with 2J2V, 2J-N_3_ (see structure in Fig. 3A), and JQ1 for 48 hours, with and without washout after 8 hours of treatment (Fig. S6†). We observed that 2J-N_3_ and JQ1 had similar cytotoxic activity, whereas 2J2V was much more potent and, importantly, largely retained its potency after washout.

We then tested the growth inhibition activity of the PROTACs on A549 spheroids (Fig. 5B), as a more complex model system mimicking the heterogeneity of the tumor microenvironment. Cytotoxicity was analyzed by measuring spheroid size and metabolic activity by CellTiter-Glo assay. On A549 spheroids, both 2J2P and 2J2V outperformed single-ligand and trivalent analogues. Also in these experiments, VHL-ligand based PROTACs, *i.e.*, MZ1 and 2J2V, exhibited superior activity over pomalidomide-based PROTACs, *i.e.*, dBET1 and 2J2P. Similar observations were also made in HCT116 spheroids (ESI Fig. S7B and C†).

### Dual-ligand PROTACs exhibit enhanced *in vivo* anti-tumor efficacy compared to conventional single-ligand PROTACs

**Fig. 6:**
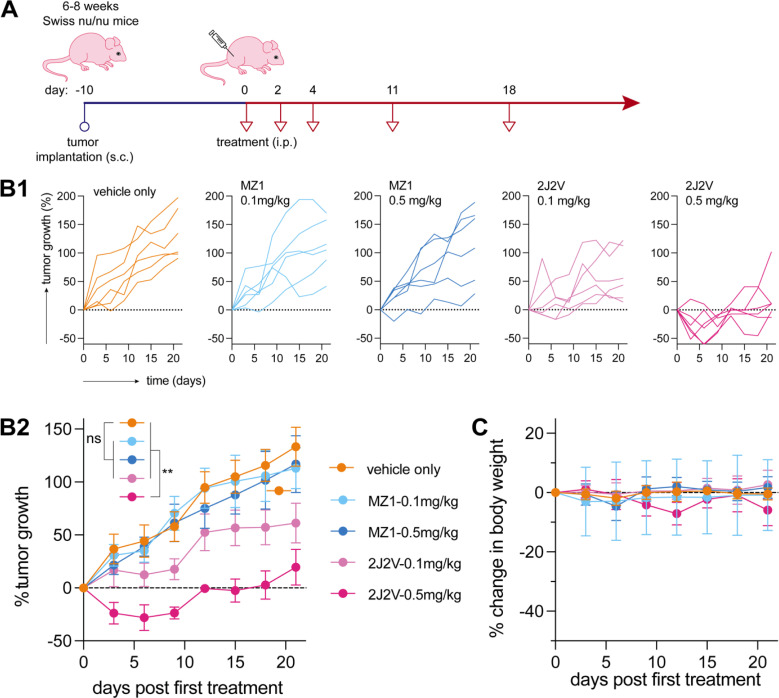
Dual-ligand PROTACs 2J2V reduce tumor growth *in vivo*. A549 lung tumor cells were xenografted in Swiss nude immunodeficient (Crl:NU(Ico)-Foxn1nu) mice. Mice were treated with 2J2V and MZ1 through i.p. administration (A) treatment timeline. (B) Tumor growth curves. The tumor growth at day *X* is defined as the value of (tumor volume at day *X*) – (tumor volume at day 0)/(tumor volume at day 0) × 100%. (C) Body weight change curves. The body weight change at day *X* is defined as the value of (body weight at day *X*) – (body weight at day 0)/(body weight at day 0) × 100%. Statistical analysis was performed by two-way ANOVA. (**: *p* < 0.01).

Based on its superior *in vitro* activity, we selected 2J2V to evaluate its therapeutic potential compared to the single-ligand PROTAC MZ1 in an A549 xenograft mouse model (Fig. 6A). Prior to *in vivo* anti-tumor evaluation, we tested the stability of MZ1 and 2J2V in mouse serum by HPLC-MS/MS, indicating that 88% of 2J2V and 95% of MZ1 remained intact after 2 hours of incubation (see ESI Table S1†), highlighting the excellent stability of both compounds. Next, Swiss nude immunodeficient (Crl:NU(Ico)-Foxn1nu) mice were subcutaneously implanted with A549 cells and tumors were allowed to grow until reaching a palpable size of approximately 100 mm^3^. Subsequently, the mice were given three consecutive intraperitoneal (i.p.) injections of MZ1 and 2J2V every other day, followed by two injections at a one-week interval. Two doses were administered, *i.e.*, 0.5 and 0.1 mg kg^−1^, respectively.

The tumor volume was monitored regularly using calipers, and the tumor growth was expressed as the percentage increase in tumor volume. While the single-ligand MZ1 PROTAC did not exhibit significant anti-tumor activity, the dual-ligand 2J2V PROTAC exhibited a potent reduction in tumor growth at the higher dose of 0.5 mg kg^−1^ (Fig. 6B). In addition, we monitored the body weight change. In response to the higher potency of 2J2V, we observed that the mice displayed a slight body weight decrease upon the injection of 2JZV at 0.5 mg kg^−1^. However, no detectable decrease in body weight was observed with 2J2V at 0.1 mg kg^−1^ (Fig. 6C).

**Fig. 7:**
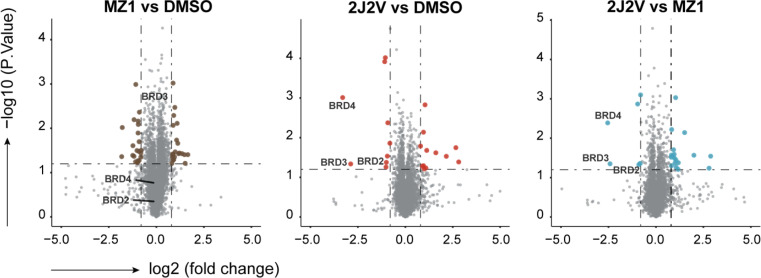
Effects of MZ1 and 2J2V treatment on the proteome of A549 cells. A549 cells were treated with 10 nM of MZ1, 2J2V or an equal volume of DMSO for 4 hours and then the cell was lysed and the protein lysate was digested and prepared for Liquid chromatography-tandem mass spectrometry analysis. Further details are in the ESI data Section 4.8.† Data are plotted as the log 2 of the normalized fold change in abundance against −log 10 of the *P* value per protein from three independent experiments. All *t*-tests performed were two-tailed assuming equal variances. Quantification of representative proteins can be found in ESI Fig. S8.†

To assess the selectivity of 2J2V for BET proteins in A549 cells, mass spectrometry proteomic experiments were performed to monitor protein levels quantitatively and unbiasedly. A549 cells were treated in triplicate with drug vehicle (0.1% DMSO), 10 nM of MZ1 or 2J2V for 4 hours. Among the 7592 proteins quantified, BRD2, BRD3 and BRD4 were identified as the top proteins being degraded the most by 2J2V compared with MZ1 or DMSO (Fig. 7). Notably, a much lower change in BET protein abundance was observed in cells treated with MZ1 (Fig. S8†). Together, these data reaffirm the superior degradation efficiency of 2J2V dual-ligand PROTAC over the conventional single-ligand PROTAC MZ1 with high selectivity and specificity.

## Conclusion

In this study, we demonstrated that dual-ligand PROTACs featuring two copies of a POI ligand and two copies of an E3 ligase ligand exhibit strongly improved activity, compared to conventional single-ligand PROTACs. Dual-ligand PROTACs were synthesized using a modular and convergent synthetic strategy using aspartic acid for homodimer synthesis and CuAAC for conjugating homodimers into dual-ligand PROTACs. *In vitro* assays revealed that these dual-ligand PROTACs exhibit potent and long-term target protein degradation. The longevity of the protein degradation is hypothesized to rely on the cell acting as an intracellular sink for these PROTACs that, owing to their bivalent nature, likely form ternary complexes between target proteins and E3 ligases with higher avidity. Furthermore, dual-ligand PROTACs outperformed conventional single-ligand PROTACs across multiple cancer cell lines, on the level of cytotoxicity. The high *in vitro* potency could be translated to an improved therapeutic response in an *in vivo* model. Our findings underscore the superior degradation capabilities of dual-ligand PROTACs, likely due to the formation of a more stable, enduring ternary complex as a result of the multivalent effect. These results were achieved without further structural optimization, suggesting that the integration of dual-ligand entities into PROTAC design holds significant promise for the advancement of targeted protein degradation therapies. Although the Ciulli group has already reported on a BET trivalent PROTACs, referred to as SIM1, as a potent degrader by simultaneously binding two bromodomains in the BET protein, herein we demonstrated that the dimerization of the E3 ligase can further improve the PROTACs activity despite the observation of the homo-degradation of the VHL E3 ligase in the case of 2J2V. Further efforts could focus on optimizing dual-ligand PROTACs to minimize self-degradation of the E3 ligase, thereby enhancing the overall efficacy of the degradation process. In addition, this study could potentially be extended to PROTACs with format of ‘2Y1P1V’, whereas two same warhead ligand Y were linked to two each E3 ligase ligand P and V. While the avidity effect could be preserved by the display of two ligand Y, the ‘2Y1P1V’ PROTAC design might offer the additional benefit of reduced sensitivity to variations in E3 ligase expression levels.

## Author contributions

Yong Chen and Zihan Xia contributed equally.

## Conflicts of interest

The authors declare no competing financial interest.

## Supplementary Material

SC-015-D4SC03555K-s001

## Data Availability

The mass spectrometry proteomics data have been deposited to the ProteomeXchange Consortium *via* the PRIDE partner repository with the dataset identifier PXD049407.
