# Supplementary material for: Dual-ligand PROTACS mediate superior target protein degradation in vitro and therapeutic efficacy in vivo
Source: Chem Sci. 2024 Oct 2;15(42):17691–701. doi: 10.1039/d4sc03555k (PMC11462456; doi:10.1039/d4sc03555k)
Supplement: SC-015-D4SC03555K-s001 [file SC-015-D4SC03555K-s001.pdf]

Supplementary Information (SI) for Chemical Science.
This journal is © The Royal Society of Chemistry 2024

Supplementary Materials for

DUAL-LIGAND PROTACS MEDIATE SUPERIOR TARGET PROTEIN DEGRADATION IN VITRO AND THERAPEUTIC EFFICACY IN VIVO

Yong Chen,[1,3#] Zihan Xia,[2,3#] Suwal *Ujjwal,* [4] Pekka Rappu,[4] Jyrki Heino,[4] Olivier De Wever[2,3*], Bruno G. De Geest[1,3*]

1 Department of Pharmaceutics, Ghent University, 9000 Ghent, Belgium.

2 Laboratory of Experimental Cancer Research, Department of Human Structure and Repair, 9000 Ghent University, Ghent, Belgium.

3 Cancer Research Institute Ghent, 9000 Ghent, Belgium.

4 Department of Biochemistry, University of Turku, 20520 Turku, Finland.

# These authors contributed equally to this work.

* corresponding authors: olivier.dewever@ugent.be, br.degeest@ugent.be

1. General information

Unless otherwise stated, all glassware was oven dried before use and all reactions were carried out under an argon atmosphere using standard Schlenk-techniques. Dry solvents were purchased from Acros Organics or Sigma-Aldrich and used without further purification. All reagents were purchased from commercial sources and were used without further purification unless otherwise stated. Reaction progress was monitored by thin layer chromatography (TLC) performed on aluminum plates coated with Kieselgel F254 with 0.2 mm thickness. Visualization was achieved by ultraviolet light (254 nm) or by staining with potassium permanganate. Flash column chromatography was performed using silica gel 60 (230-400 mesh, Merck ans co.). Mass spectra were obtained using a Finnigan MAT 8200 (70 eV), an Agilent 5973 (70 eV), using electrospray ionization (ESI) or electron impact ionization (EI). All ^{1}H NMR, ^{13}C NMR NMR were recorded on a BrukerAV-400 spectrometer in Chloroform-d1 or DMSO-d6. Chemical shifts are given in parts per million (ppm), referenced to tetramethylsilane using the solvent peak as internal standard ($CDCl_3$: ^{1}H = 7.26 ppm, ^{13}C = 77.16 ppm; CD_3SOCD_3: ^{1}H = 2.50 ppm, ^{13}C = 39.52 ppm). Coupling constants were quoted in Hz. ^{1}H NMR splitting patterns were designated as singlet (s), broad (brd), doublet (d), triplet (t), quartet (q), pentet (p), sextet (se), septet (sep), octet (o) or combinations thereof. Splitting patterns that could not be interpreted were designated as multiplet (m).

2. Abbreviations

RT: Room temperature

Atm: Atmospheric pressure

TLC: Thin layer chromatography

Sat.: Saturated

Aq.: Aqueous

Ar: Aromatic

H: hour

Min: minute

DCM: Dichloromethane

DMF: Dimethylformamide

TFA: Trifluoroacetic acid

HATU: Hexafluorophosphate azabenzotriazole tetramethyl uranium

DIPEA: N,N-Diisopropylethylamine

Et_3N: Triethylamine

DCC: N,N′-Dicyclohexylcarbodiimide

DMAP: 4-Dimethylaminopyridine

Cbz: Benzyloxycarbonyl group

LRMS: Low resolution mass spectrometry

LCMS: Liquid chromatography-mass spectrometry

TBTA: Tris(benzyltriazolylmethyl)amine

3. Chemical synthesis

3.1 Synthesis of 1J-N_3 and 2J-N_3

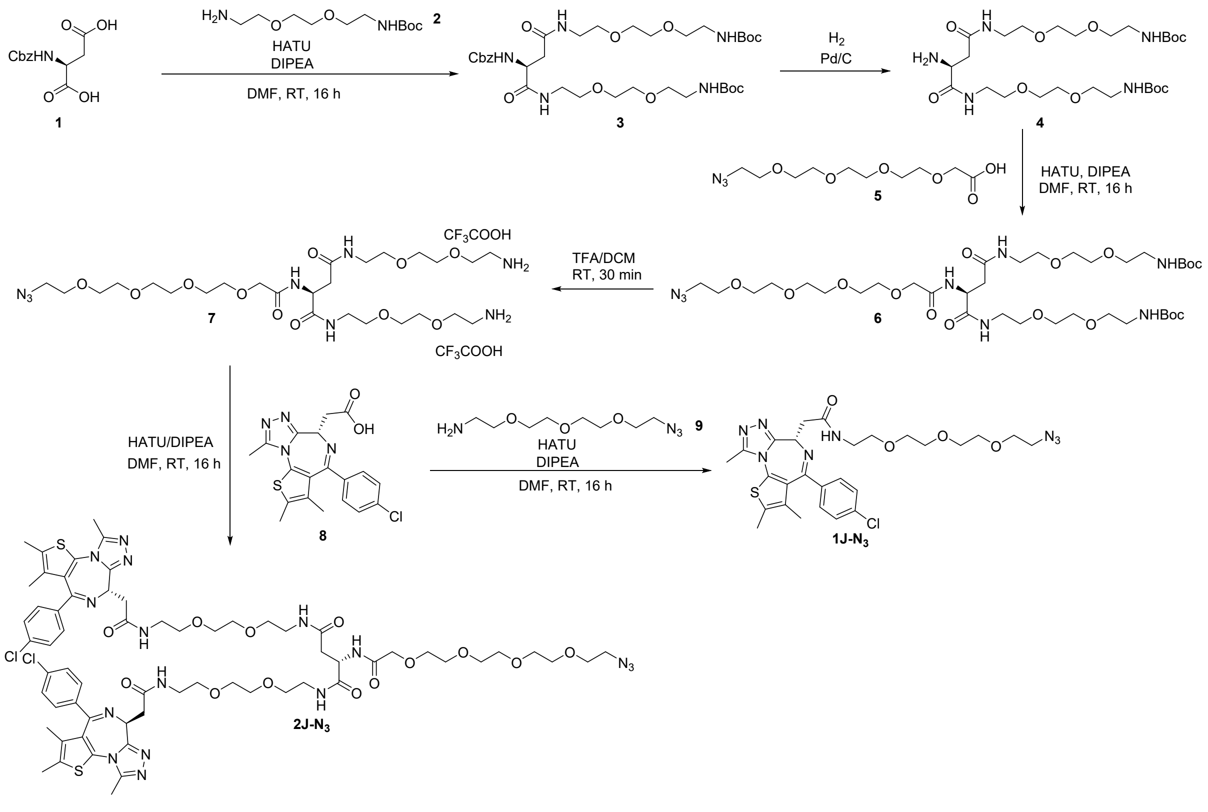

Scheme 1, synthetic route to 1J-N_3 and 2J-N_3.

<u>Synthesis of compound 3</u>

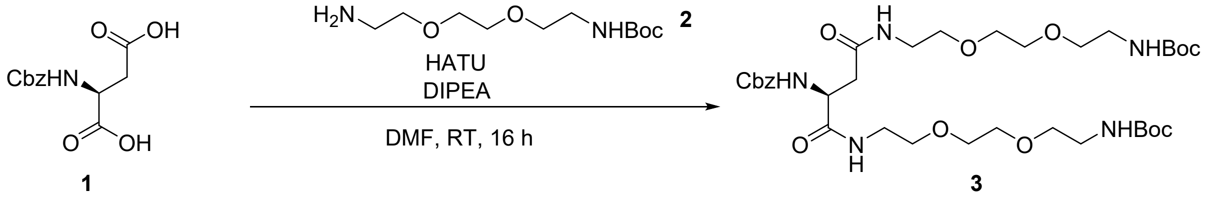

Compound **1** (938 mg, 3.51 mmol, 1.0 equiv.) was dissolved in DMF (10 mL). Then at 0 °C, DIPEA (1.60 ml, 10.5 mmol, 3.0 equiv.) was added, followed by HATU (2.94 g, 7.72 mmol, 2.2 equiv.). The mixture was stirred at 0 °C for 10 min. Then compound **2** (2.09 g, 8.43 mmol, 2.4 equiv.) in DMF (5.0 mL) was added, and the mixture was stirred and allowed to warm to room temperature for 16 h. Then DMF was removed under vacuum. The mixture was redissolved by DCM, washed by saturated NH_4Cl (aq.) two times, brine one time. The organic phase was then dried over Na_2SO_4, filtered, and concentrated. The crude product was then purified by silica gel column chromatography (DCM/MeOH = 20:1 to 15:1) to give desired compound **3** (2.12 g, 83% yield) as a colorless oil.

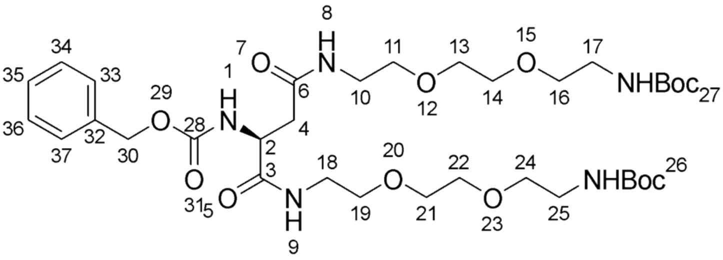

^{1}H NMR (400 MHz, DMSO-*d*$_6$) δ 7.84 (q, *J* = 5.6 Hz, 2H, **NH8, NH9**), 7.38-7.31 (m, 6H, **Ar-H, NH1**), 6.75 (t, *J* = 5.8 Hz, 2H, **BocNH**), 5.06-4.98 (m, 2H, **H30**), 4.31 (td, *J* = 8.3, 5.3 Hz, 1H, **H2**), 3.48 (brd, 8H, **H13, H14, H21, H22**), 3.39-3.34(m, 8H, **H11, H16, H19, H24**), 3.20-3.16 (m, 4H, **H10, H18**), 3.05 (app. q, *J* = 6.1 Hz, 4H, **H17, H25**), 2.48 (partly immersed in DMSO-d6, 1H, **H4**), 2.37 (dd, *J* = 15.0, 8.5 Hz, 1H, **H4**), 1.37 (s, 18H, **Boc-Me**). **^{13}C NMR (100 MHz, DMSO-*d*$_6$)** δ 171.1, 169.3, 155.65, 155.58, 136.9 (**C32**), 128.3 (**Ar-C**), 128.0 (**Ar-C**), 127.7 (**Ar-C**), 77.6 (O**C**Me3), 69.5, 69.4, 69.2, 69.0, 68.8, 65.5 (**C30**), 54.9 (**DCM**), 51.8 (**C2**), 39.7 (**C17, C25**), 38.6 (**C10, C18**), 37.7 (**C4**), 28.2 (OC(**C**H$_3$)$_3$). **LRMS (ESI) (m/z)**: calculated for $[M+H]^+$ ($C_{34}H_{58}O_{12}N_5$) requires 728.4, found: 728.3.

<u>Synthesis of compound 25</u>

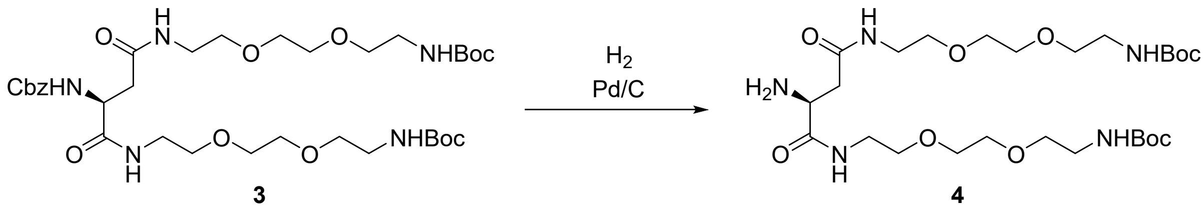

3 **4**

Compound **3** (2.12 g, 2.91 mmol, 1.0 equiv.) was dissolved in MeOH (25 mL) in a two-neck round-bottom flask, then Pd/C (10% w/w, 310 mg, 0.291 mmol, 0.1 equiv.) was added under N_2. Then 1 atm. H_2 balloon was inserted onto the septum of the flask through a syringe needle. The mixture was stirred at room temperature for about 4 hours. Use TLC to monitor the reaction. Once compound **3** was consumed, the Pd/C was filtered off and washed by DCM. The filtrate was then collected, concentrated and the crude product **4** was used in the next step without further purification.

Note: during filtration, do not let the solvent above Pd/C go dry. Continuous sucking of air through Pd/C can cause fire. The Pd/C were then immediately stored in a waste bottle with water.

Synthesis of compound 6

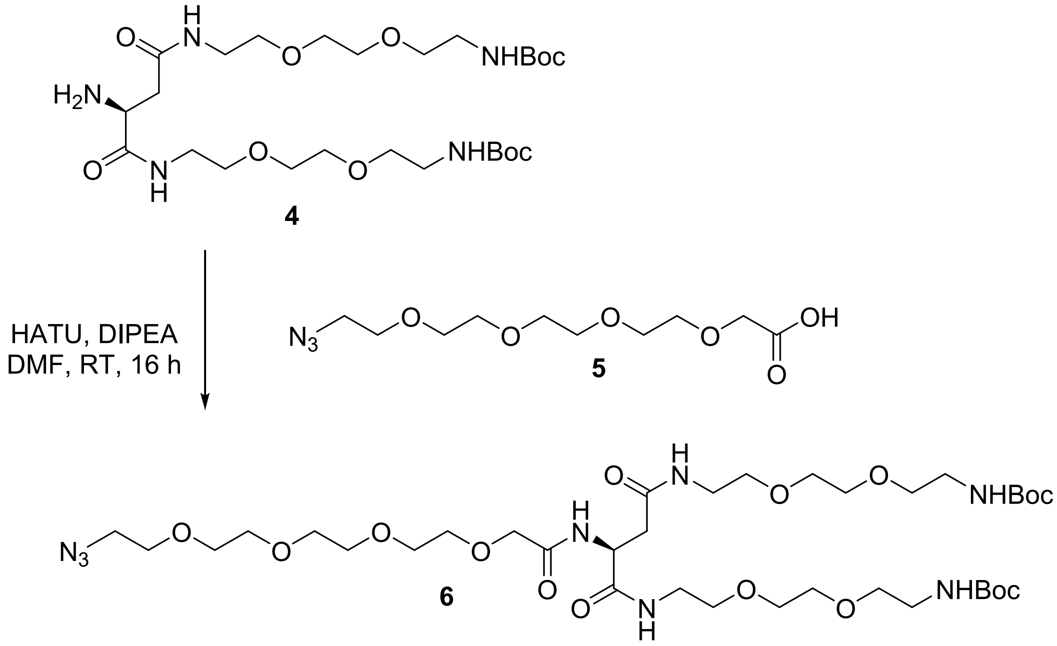

Commercially available compound **5** (140 µl, 0.5 M in ^{t}BuOMe, 0.070 mmol, 1.0 equiv.) and DIPEA (24 µl, 0.14 mmol, 2.0 equiv.) were dissolved in DMF (1.0 mL), then HATU (29 mg, 0.077 mmol, 1.1 equiv.) was added. After stirring at room temperature for 5 min, compound **25** (50 mg, 0.084 mmol, 1.2 equiv.) was added. The mixture was stirred at room temperature for 12 h. Then DMF was removed by rotavapor, and the mixture was diluted by DCM, washed by saturated NH_4Cl (aq.) 1 time, brine 1 time. The organic phase was then dried over Na_2SO_4, filtered, and concentrated. The crude product was then purified by silica gel column chromatography (DCM/MeOH = 20:1 to 10:1) to give desired compound **6** (52 mg, 86% yield) as a light yellowish oil.

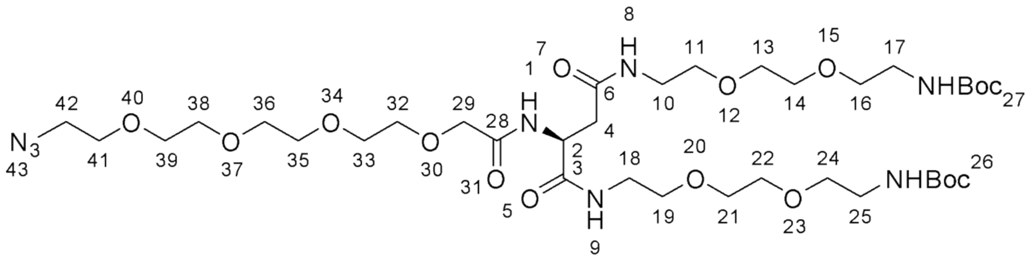

^{1}H NMR (400 MHz, $CDCl_3$) δ 7.97 (d, *J* = 7.8 Hz, 1H, **NH-1**), 7.29 (s, 1H, **NH**), 6.57 (s, 1H, **NH**), 5.24 (s, 1H, **NH**), 5.13 (s, 1H, **NH**), 4.81-4.76 (m, 1H, **H2**), 4.05 (d, *J* = 3.4 Hz, 2H, **H29**), 3.71-3.66 (m, 14H), 3.61-3.59 (m, 8H), 3.56-3.53 (m, 8H), 3.45-3.42 (m, 4H, **H10, H18**), 3.40 (*J* = 5.0 Hz, 2H, **H42**), 3.31 (brd, 4H, **H17, H25**), 2.80 (dd, *J* = 15.1, 4.7 Hz, 1H, **H4**), 2.64 (dd, *J* = 15.1, 6.2 Hz, 1H, **H4**), 1.44 (s, 18H, **Boc-H**). **^{13}C NMR (100 MHz, $CDCl_3$)** δ 171.23, 171.20, 170.8, 170.6, 156.3, 71.2, 70.82, 70.80, 70.75, 70.69, 70.6, 70.45, 70.42, 70.37, 70.3, 70.1,

69.6, 54.5, 50.8, 49.6 (**C2**), 42.7, 39.73, 39.66, 37.7 (**C4**), 28.6 (**Boc-CH$_3$**) ppm. **LRMS (ESI) (m/z)**: calculated for $[M+H]^+$ ($C_{36}H_{69}O_{15}N_8$) requires 853.5, found: 853.4.

Synthesis of 2J-N$_3$

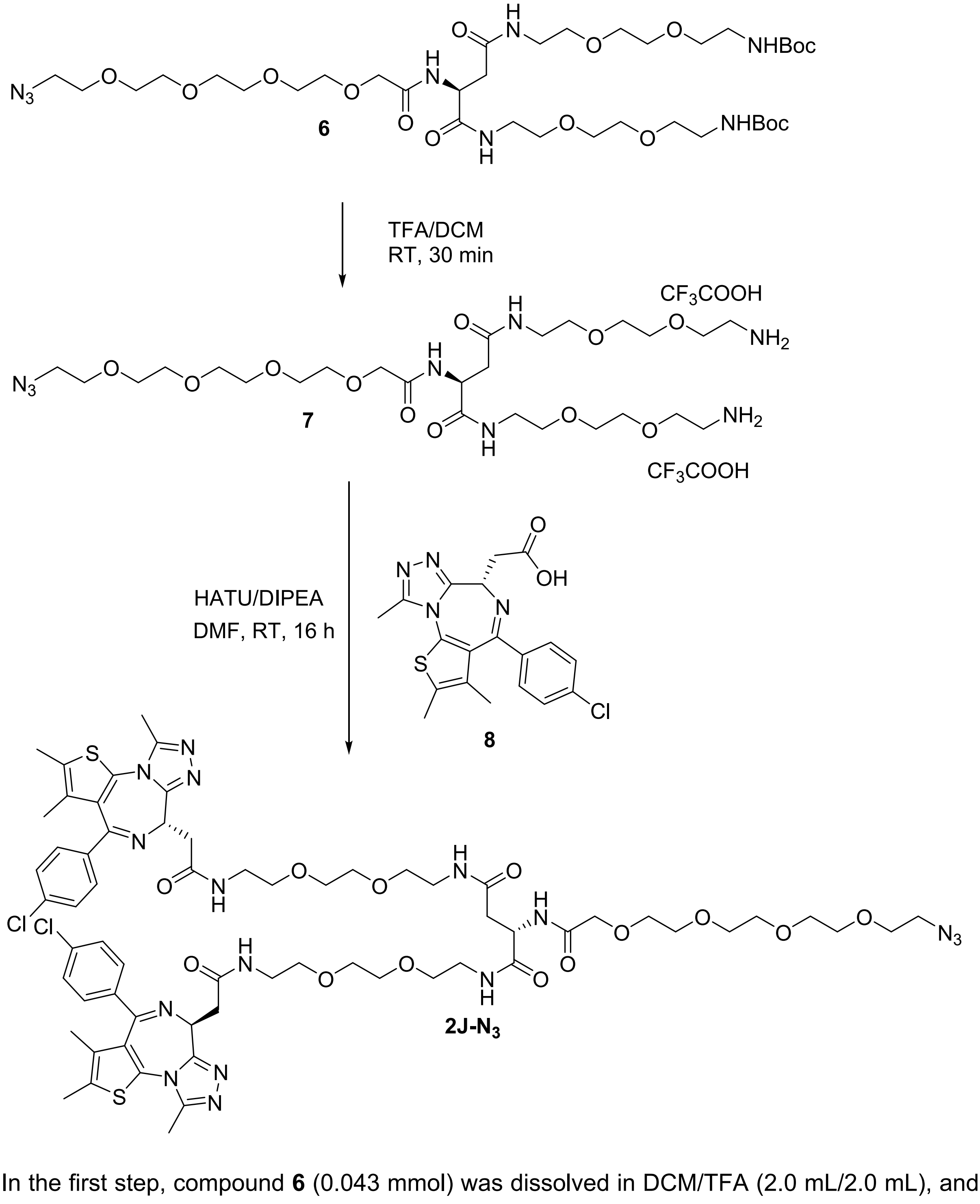

In the first step, compound **6** (0.043 mmol) was dissolved in DCM/TFA (2.0 mL/2.0 mL), and the mixture was stirred at room temperature for 1 h. The solvent was evaporated under vacuum, and the crude residue was then co-evaporated with toluene 3 times and then crude compound **7** was used in the next step without further purification.

In the second step, the JQ1 acid **8** (21 mg, 0.053 mmol, 2.4 equiv.) and DIPEA (38 μl, 0.22 mmol, 10 equiv.) were dissolved in DMF (1.5 mL). Then at room temperature HATU (20 mg, 0.053 mmol, 2.4 equiv.) was added. The mixture was stirred at room temperature for another 5 min. Then half of the crude product **7** from above step (about 0.22 mmol, 1.0 equiv.) in DMF (1.0 mL) was added. The mixture was stirred at room temperature for 12 h. Then DMF was removed under vacuum. The mixture was redissolved by DCM, washed by saturated NH_4Cl (aq.). one time and the aqueous phase was extracted by DCM 1 time. The combined organic phase was washed by brine, dried over Na_2SO_4, filtered, and concentrated. The crude mixture was then purified by preparative TLC plate (silica gel, DCM/MeOH = 5:1) to give desired compound **2J-N$_3$** (15 mg, 50% yield) as a colorless oil.

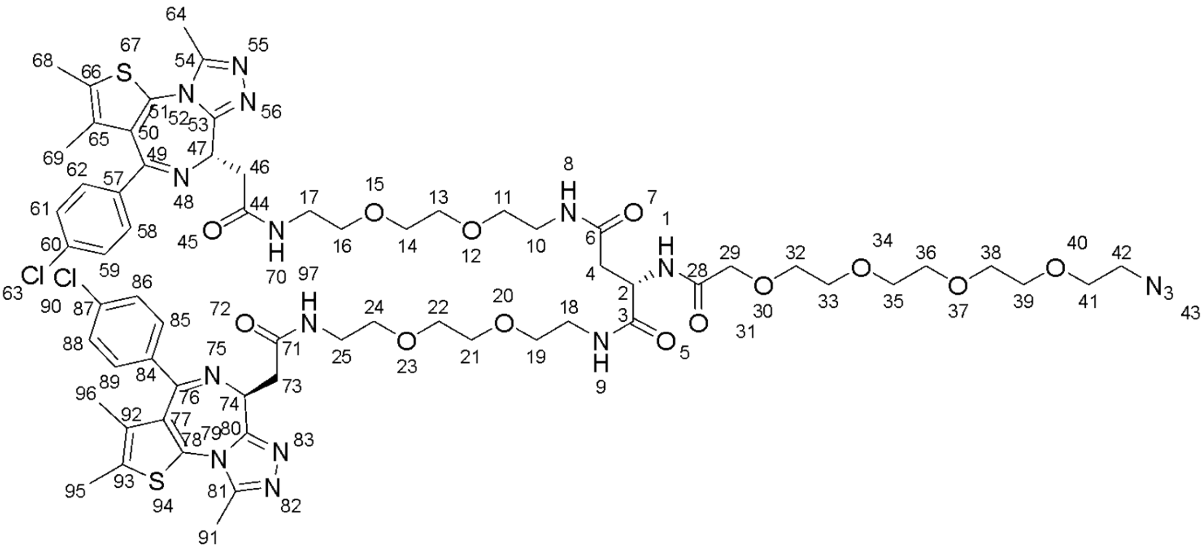

^{1}H NMR (400 MHz, Chloroform-*d*) δ 8.02 (d, *J* = 7.9 Hz, 1H), 7.54 (t, *J* = 5.7 Hz, 1H), 7.41-7.36 (m, 6H), 7.33-7.30 (m, 4H), 4.85-4.80 (m, 1H, **H2**), 4.68-4.63 (m, 2H, **H47, H74**), 4.02 (d, *J* = 2.1 Hz, 2H, **H29**), 3.68-3.34 (m, 42H), 2.78 (dd, *J* = 15.1, 5.7 Hz, 1H, **H4**), 2.67-2.61 (m, 7H, **including H4, Ar-CH3 X2**), 2.39 (s, 6H, **Ar-CH3 X2**), 1.66 (s, 6H, **Ar-CH3 X2**). **^{13}C NMR (100 MHz, CDCl$_3$)** δ170.90, 170.88, 170.78, 170.72, 170.4, 164.0, 163.9, 155.86, 155.80, 150.06, 150.00, 136.88, 136.81, 136.74, 132.22, 132.19, 131.06, 130.99, 130.90, 130.7, 130.0, 128.82, 128.78, 71.1, 70.77, 70.76, 70.73, 70.68, 70.65, 70.46, 70.44, 70.38, 70.12, 70.07, 69.98, 69.93, 69.75, 54.52 (**C47 or C74**), 54.47 (**C47 or C74**), 50.8, 49.9 (**C2**), 39.59, 39.52, 39.2, 38.0, 29.8, 14.5 (**Ar-CH3**), 13.2 (**Ar-CH3**), 11.9 (**Ar-CH3**). **LRMS (ESI) (m/z)**: calculated for $[M+2H]^{2+}$ ($C_{64}H_{84}O_{13}N_{16}S_2Cl_2$) requires 709.3, found: 709.3.

Synthesis of 1J-N_3

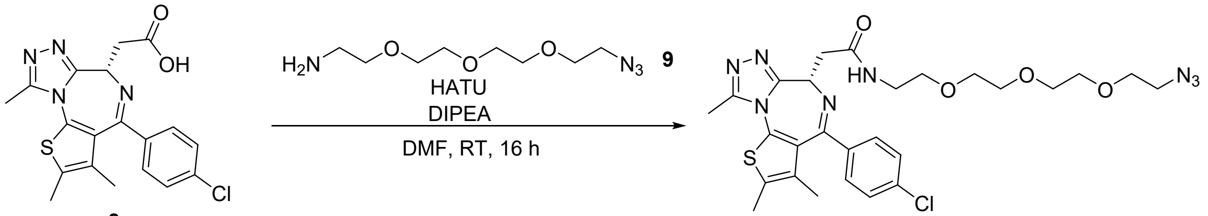

8 **1J-N_3**

JQ1 acid **8** (10 mg, 0.025 mmol, 1.0 equiv.) and DIPEA (12 μl, 0.070 mmol, 2.8 equiv.) were mixed in DMF (1.0 mL) and then HATU (11 mg, 0.030 mmol, 1.1 equiv.) was added. The mixture was stirred at room temperature for 5 min. Afterwards, commercially available compound **9** (7.6 mg, 0.035 mmol, 1.3 equiv.) was added. The mixture was stirred at room temperature for 12 h. Then the crude reaction mixture was diluted by DCM, washed by 1 M NaOH (aq.), 1 M HCl (aq.) and then brine. The organic phase was then dried over Na_2SO_4, filtered, and concentrated. The crude mixture was then purified by preparative TLC plate (DCM/MeOH = 15:1) to give **1J-N_3** (10 mg, 67% yield) as a colorless oil.

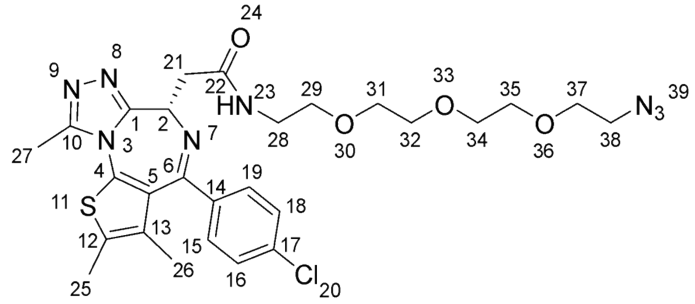

^{1}H NMR (400 MHz, Chloroform-*d*) δ 7.41 (d, *J* = 8.3 Hz, 2H, **Ar-H**), 7.33 (d, *J* = 8.3 Hz, 2H, **Ar-H**), 6.77 (t, *J* = 5.5 Hz, 1H, **NH-23**), 4.65 (t, *J* = 7.0 Hz, 1H, **H2**), 3.69-3.67 (m, 10H), 3.63-3.59 (m, 2H), 3.55-3.49 (m, 3H, including **H28, H21**), 3.41-3.55 (m, 3H, including **H21, H38**), 2.67 (s, 3H, **Ar-CH3**), 2.40 (s, 3H, **Ar-CH3**), 1.67 (s, 3H, **Ar-CH3**). **^{13}C NMR (100 MHz, $CDCl_3$)** δ 170.6, 164.0, 155.8, 150.0, 136.9, 136.8, 132.3, 131.1, 130.9, 130.6, 130.0 (**C15 or C16**), 128.8 (**C15 or C16**), 70.9, 70.81, 70.80, 70.6, 70.2, 69.9, 54.5 (**C2**), 50.8 (**C38**), 39.6 (**C28 or C21**), 39.3 (**C28 or C21**), 14.5 (**Ar-CH3**), 13.2 (**Ar-CH3**), 12.0 (**Ar-CH3**). **LRMS (ESI) (m/z)**: calculated for $[M+H]^+$ ($C_{27}H_{34}O_4N_8S_1Cl_1$) requires 602.1, found: 602.1.

3.2 Synthesis of 1P-alkyne and 2P-alkyne

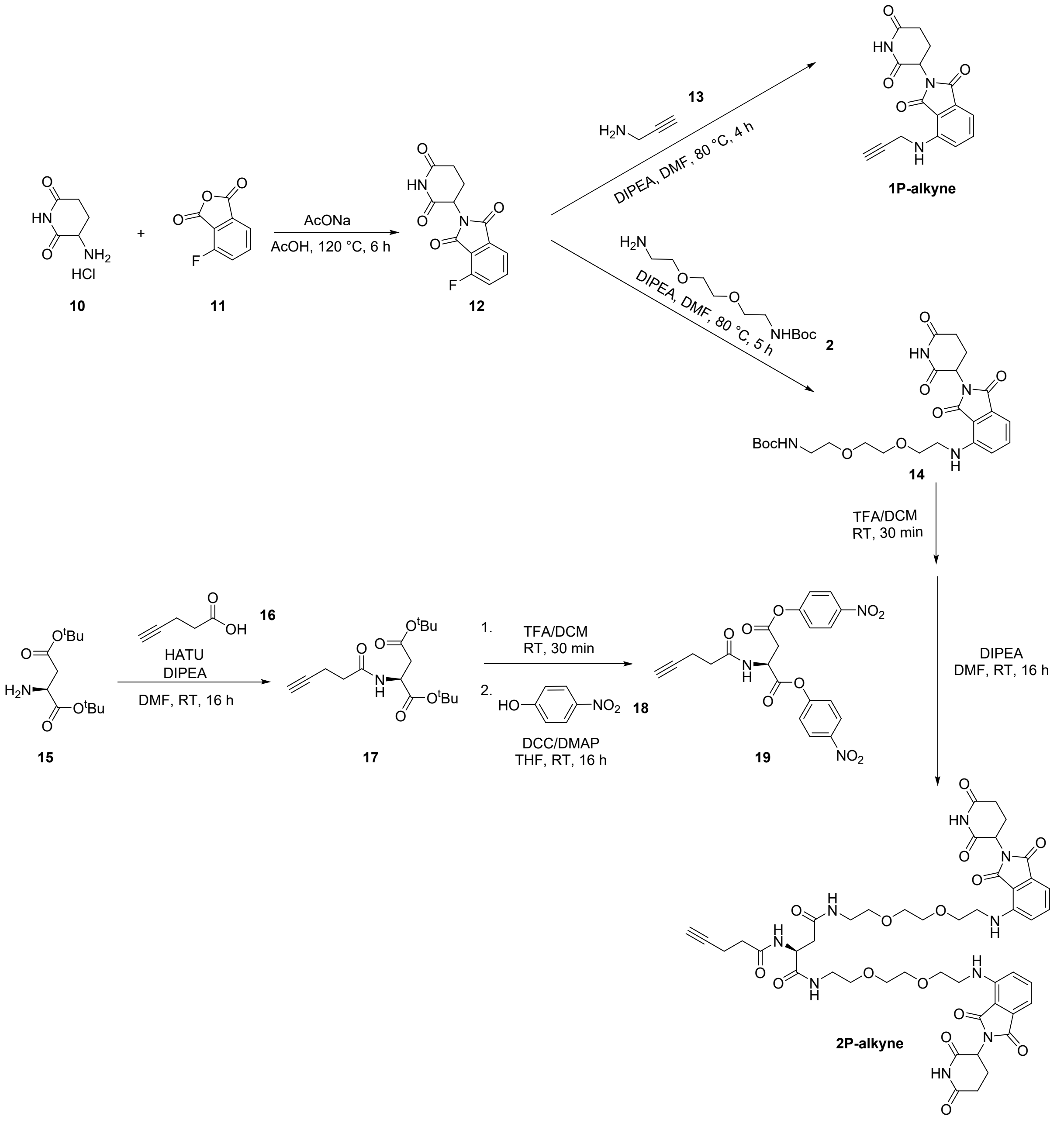

Scheme 2, synthetic route to 1P-alkyne and 2P-alkyne.

Synthesis of compound 12

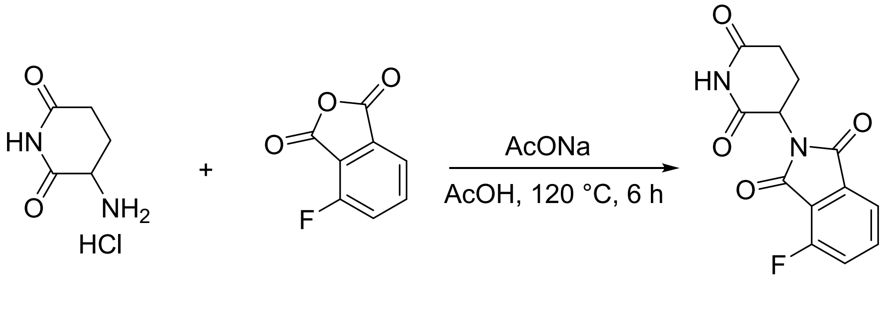

10 11 12

Note: Compound **12** was synthesized according to the known procedure (*J. Med. Chem.*, **2022**, *65*, 9096-9125.).

Compound **10** (823 mg, 5.0 mmol, 1.0 equiv.), compound **11** (1.25 g, 7.5 mmol, 1.5 equiv.) and sodium acetate (492 mg, 6.0 mmol, 1.2 equiv.) were mixed in acetic acid (20 mL) and then refluxed for 6 hours. After cooling, the reaction mixture was poured onto water and the solid was collected by filtration. The solid was then further washed with water, ether and dried in vacuum. The obtained grey solid (980 mg, 71% yield) was used directly in the next step without further purification. The obtained ^{1}H NMR spectra data of compound **12** were in good agreement with the literature (*J. Med. Chem.*, **2022**, *65*, 9096-9125.).

Synthesis of 1P-N_3

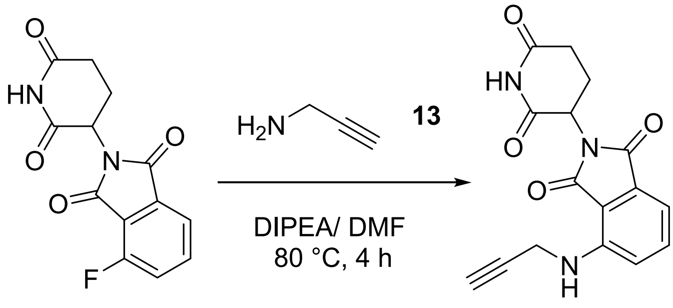

12 **1P-alkyne**

Note: Compound **1P-alkyne** were synthesized according to the known procedure (*J. Med. Chem.*, **2019**, *62*, 7042-7057.).

Compound **12** (400 mg, 1.45 mmol, 1.0 equiv.), compound **4** (139 μl, 2.17 mmol, 1.5 equiv.) and DIPEA (506 μl, 2.90 mmol, 2.0 equiv.) were mixed in DMF (5.0 mL) and then stirred at 80 °C for 4 hours. After cooling, the reaction mixture was concentrated and directly purified by silica gel column chromatography (hexane/ethyl acetate = 1/1, then DCM/MeOH = 30/1) to give **1P-alkyne** as a yellowish oil (125 mg, 28% yield). The obtained ^{1}H NMR spectra data of **1P-alkyne** were in good agreement with the literature (*J. Med. Chem.*, **2019**, *62*, 7042-7057.).

Synthesis of compound 17

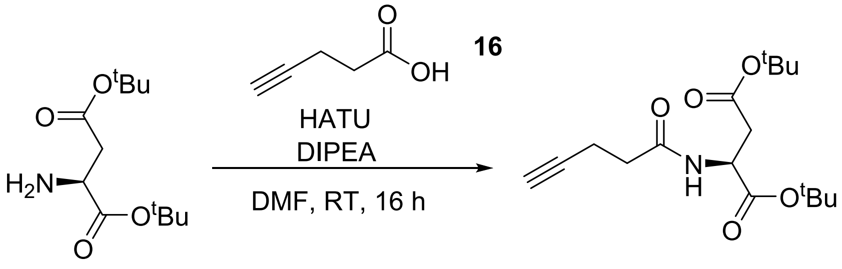

15 **17**

Compound **16** (490 mg, 5.00 mmol, 1.0 equiv.) and DIPEA (1.75 mL, 10.0 mmol, 2.0 equiv.) were first dissolved in DMF (10.0 mL). Then at 0 °C, HATU (1.90 g, 5.00 mmol, 1.0 equiv.) was added, the mixture was stirred at 0 °C for 10 min. Then compound **15** (1.41 g, 5.0 mmol, 1.0 equiv.) was added, and the mixture was allowed to warm to room temperature over 12 h. Afterwards, DMF was first removed under vacuum. The mixture was dissolved by DCM and washed by Sat. NH_4Cl (aq.) and brine. The organic phase was then dried over Na_2SO_4, filtered, and concentrated. The resulting residue was purified by silica gel column chromatography (hexane/ethyl acetate = 3:1) to deliver compound **17** (1.42 g, 87% yield) as a colorless oil.

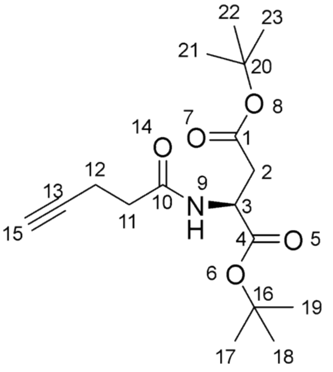

^{1}H NMR (400 MHz, DMSO-*d*$_6$) δ 8.26 (d, *J* = 8.1 Hz, 1H, **NH9**), 4.50-4.45 (m, 1H, **H3**), 2.74 (t, *J* = 2.4 Hz, 1H, **H15**), 2.63 (dd, *J* = 16.1, 6.2 Hz, 1H, **H2**), 2.50 (dd, *J* = 16.1, 7.3 Hz, 1H, **H2**, **immersed in DMSO-*d*$_6$ peaks**), 2.37-2.28 (m, 4H, **H11 & H12**), 1.40 (s, 9H, **^{t}Bu-H**), 1.38 (s, 9H, **^{t}Bu-H**) ppm. **^{13}C NMR (100 MHz, DMSO-*d*$_6$)** δ 170.2 (**C10**), 169.7 (**C4 or C1**), 169.1 (**C4 or C1**), 83.5 (**C13**), 80.9 (**C16 or C20),** 80.4 (**C16 or C20**), 71.3 (**C15**), 49.3 (**C3**), 37.3 (**C2**), 33.8 (**C11**), 27.7 (**^{t}Bu-CH$_3$**), 27.5 (**^{t}Bu-CH$_3$**), 14.1 (**C12**) ppm. **LRMS (ESI) (m/z)**: calculated for $[M+H]^+$ ($C_{17}H_{28}O_5N_1$) requires 326.2, found: 326.2.

Synthesis of compound 19

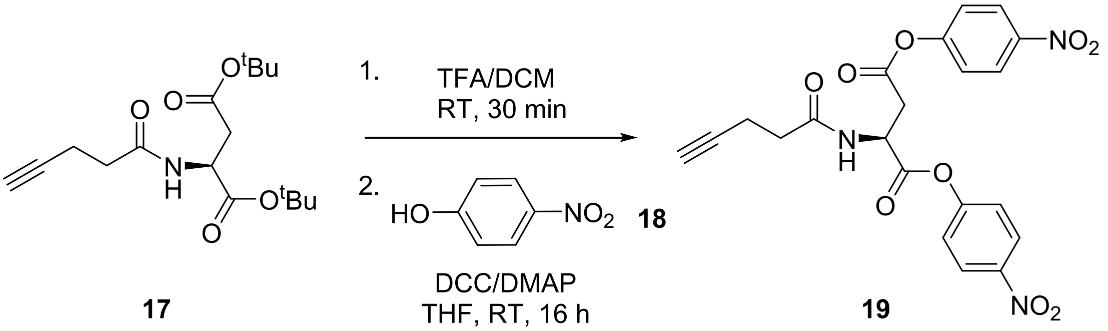

17 **19**

In the first step, compound **17** (1.42 g) was dissolved in DCM/TFA (5.0 mL/2.0 mL) and stirred at room temperature for 30 min. The solvent was removed by rotavapor and the crude diacid was obtained as a colorless solid and used in the next step without further purification.

In the second step, the crude diacid (238 mg, 1.12 mmol, 1.0 equiv.) was dissolved in THF (5.0 mL) and at 0 °C, para-nitrophenol **18** (343 mg, 2.46 mmol, 2.2 equiv.) and DMAP (13 mg, 0.11 mmol, 0.1 equiv.) were added, followed by DCC (507 mg, 2.46 mmol, 2.2 equiv.). The mixture was stirred at 0 °C and allowed to warm to room temperature over 12 h. Then the precipitate was filtered off, and the filtrate was collected, concentrated. The solid was formed again upon concentration, which was then filtered and washed by DCM. The remaining solid was then collected to give compound **19** (116 mg, 23% yield) as a colorless solid. (**Note**: the product contains trace amount of free para-nitrophenol, but this contamination did not influence the next reaction.)

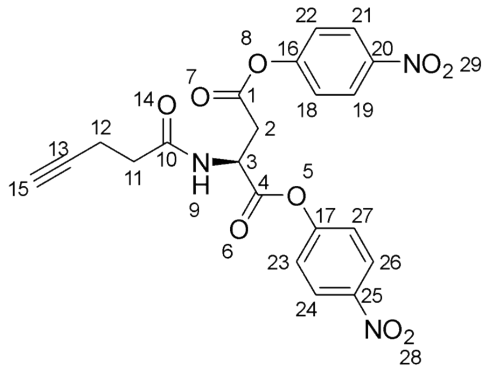

^{1}H NMR (400 MHz, Chloroform-*d*) δ 8.30-8.27 (m, 4H, **H19, H21, H24, H26**), 8.16 (d, *J* = 9.1 Hz, 1H, **free para-nitrophenol**), 7.32-7.26 (m 4H, **H18, H22, H23, H27**), 6.91 (d, *J* = 9.1 Hz, 1H, **free para-nitrophenol**), 6.77 (d, *J* = 7.7 Hz, 1H, **free para-nitrophenol**), 5.25 (dt, *J* = 7.6, 4.6 Hz, 1H, **H3**), 3.48 (dd, *J* = 17.7, 4.7 Hz, 1H, **H2**), 3.38 (dd, *J* = 17.6, 4.5 Hz, 1H, **H2**), 2.59-2.55 (m, 4H, **H11, H12**), 1.98 (d, *J* = 2.5 Hz, 1H, **H15**). **^{13}C NMR (4100 MHz, Chloroform-*d*)** δ 171.4, 169.2, 168.6, 126.4 (**free para-nitrophenol**), 125.57, 125.54, 122.45, 122.37, 115.8 (**free para-nitrophenol**), 82.5 (**C13**), 70.0 (**C15**), 49.0 (**C3**), 36.8 (**C2**), 35.2 (**C11**), 14.9 (**C12**). **LRMS (ESI) (m/z)**: calculated for $[M+H]^+$ ($C_{21}H_{18}O_9N_3$) requires 456.1, found: 456.2.

<u>Synthesis of compound 14</u>

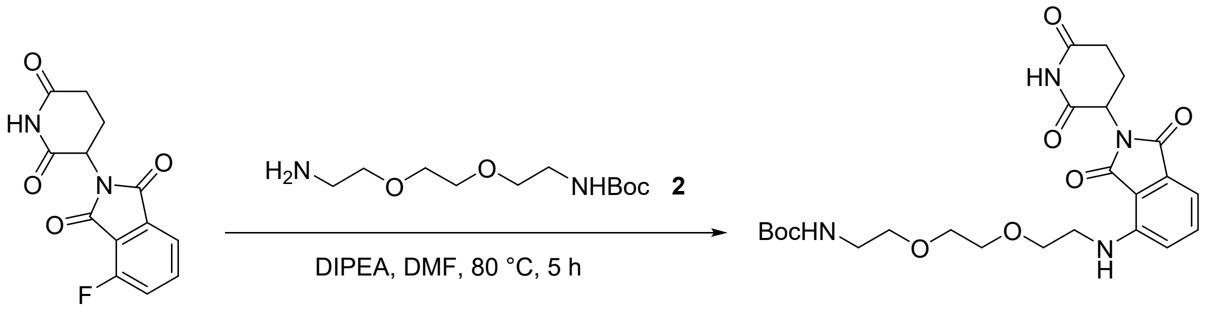

12 **14**

Compound **12** (400 mg, 1.45 mmol, 1.0 equiv.), compound **2** (539 mg, 2.17 mmol, 1.5 equiv.) and DIPEA (506 μl, 2.90 mmol, 2.0 equiv.) were mixed in DMF (5.0 mL) and then stirred at 80 °C for 4 hours. After cooling, the reaction mixture was concentrated and directly purified by silica gel column chromatography (hexane/ethyl acetate = 1/1, then DCM/MeOH = 30/1) to give compound **14** (263 mg, 36% yield) as a yellowish oil.

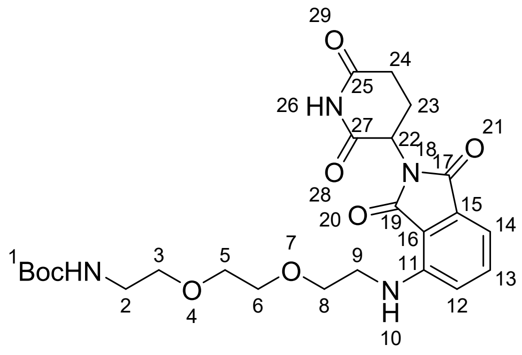

^{1}H NMR (400 MHz, DMSO-d_6) δ 11.09 (s, 1H, **NH26**), 7.58 (dd, *J* = 8.4, 7.2 Hz, 1H, **H13**), 7.15 (d, *J* = 8.6 Hz, 1H, **H12**), 7.04 (d, *J* = 7.1 Hz, 1H, **H14**), 6.73 (t, *J* = 5.5 Hz, 1H, **Boc-NH**), 6.61 (t, *J* = 5.8 Hz, 1H, **NH10**), 5.06 (dd, *J* = 13.0, 5.3 Hz, 1H, **H22**), 3.62 (t, *J* = 5.4 Hz, 2H), 3.56 (dd, *J* = 6.2, 3.3 Hz, 2H), 3.51 (dd, *J* = 6.1, 4.0 Hz, 2H), 3.47 (q, *J* = 5.5 Hz, 2H, **H9**), 3.38 (t, *J* = 6.1 Hz, 2H), 3.05 (q, *J* = 6.0 Hz, 2H, **H2**), 2.93-2.84 (m, 1H, **H24**, immersed in DMF peak), 2.63-2.52 (m, 2H, including **H23**, **H24**), 2.06-1.99 (m, 1H, **H23**), 1.36 (brd, 9H, **Boc-H**). **^{13}C NMR (100 MHz, DMSO-d_6)** δ 172.8, 170.1, 168.9, 167.3, 162.3, 155.6, 146.4, 136.2 (**C13**), 132.1, 117.4 (**C12**), 110.7 (**C14**), 109.3, 77.6, 69.7, 69.5, 69.2, 68.9, 48.6 (**C22**), 41.7 (**C9**), 39.7 (**C2**), 35.8 (**DMF-CH3**), 31.0 (**DMF-CH3**), 30.8 (**C24**), 28.2 (**Boc-CH3**), 22.1 (**C23**). **LRMS (ESI) (m/z)**: calculated for $[M-H]^-$ ($C_{24}H_{31}O_8N_4$) requires 503.2, found: 503.2.

<u>Synthesis of 2P-alkyne</u>

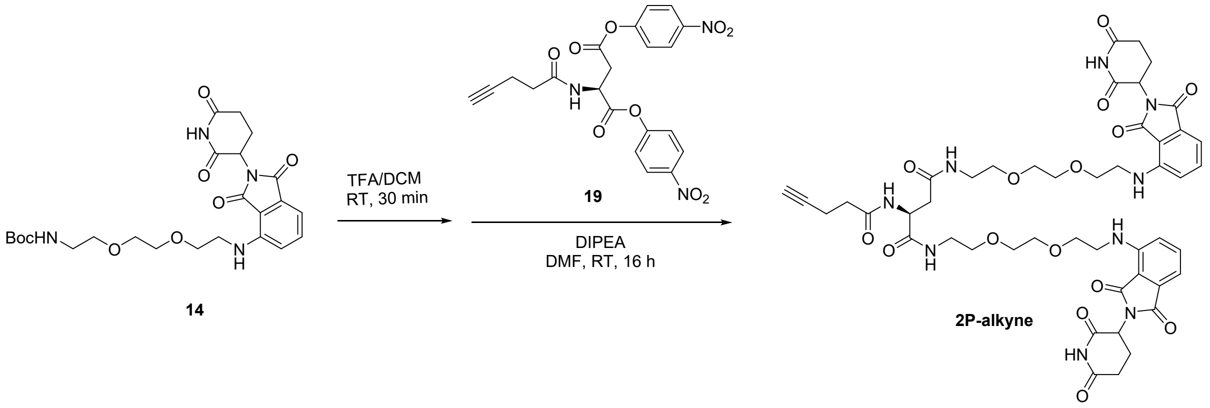

In the first step, compound **14** (172 mg, 0.34 mmol, 2.4 equiv.) was dissolved in DCM/TFA (5.0 mL/2.0 mL) and stirred at room temperature for 30 min. The solvent was removed by rotavapor and the crude product was used in the next step without further purification.

In the second step, compound **19** (64 mg, 0.14 mmol, 1.0 equiv.), crude product from above (176 mg, 0.34 mmol, 2.4 equiv.) and DIPEA (210 μl, 1.41 mmol, 10.0 equiv.) was mixed in DMF (3.0 mL). The mixture was stirred at room temperature for 16 h. Afterwards, DMF was removed under vacuum, and the crude residue was purified by silica gel column chromatography (DCM/MeOH = 20:1 to 10:1) to deliver compound **2P-alkyne** (100 mg, 72% yield) as a yellowish foam.

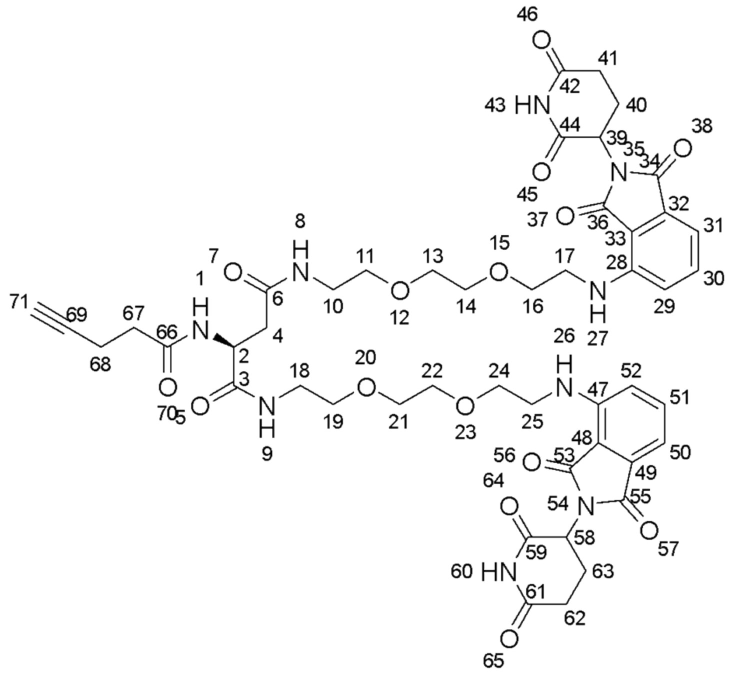

^{1}H NMR (400 MHz, DMSO-d_6) δ 11.08 (s, 2H, **H43, H60**), 8.04 (d, *J* = 8.1 Hz, 1H, **H1**), 7.83 (t, *J* = 5.6 Hz, 1H, **H8 or H9**), 7.69 (t, *J* = 5.7 Hz, 1H, **H8 or H9**), 7.58 (dd, *J* = 8.6, 7.1 Hz, 2H, **H30, H51**), 7.14 (d, *J* = 8.6 Hz, 2H, **H29, H52**), 7.03 (d, *J* = 7.0 Hz, 2H, **H31, H50**), 6.60 (t, *J* = 5.8 Hz, 2H, **H26, H27**), 5.05 (dd, *J* = 12.9, 5.3 Hz, 2H, **H39, H58**), 4.56-4.50 (m, 1H, **H2**), 3.61 (t, *J* = 5.4 Hz, 4H), 3.57-3.54 (m, 4H), 3.52-3.49 (m, 4H), 3.47 (q, *J* = 5.6 Hz, 4H), 3.38 (td, *J* = 6.0, 1.5 Hz, 4H), 3.17 (p, *J* = 5.8 Hz, 4H, **H10, H18**), 2.88 (ddd, *J* = 17.4, 14.2, 5.5 Hz, 2H, **H41, H62**), 2.75 (t, *J* = 2.1 Hz, 1H, **H71**), 2.61-2.47 (m, 5H, overlapped with DMSO peaks, including **H40, H63, H41, H62**), 2.37-2.31 (m, 5H, **including H68, H4**), 2.06-1.99 (m, 2H, **H40, H63**). **^{13}C NMR (100 MHz, DMSO-d_6)** δ 172.8, 170.8, 170.2, 170.1, 169.3, 169.9, 167.3, 146.4, 136.2, 132.1, 117.4, 110.7, 109.2, 83.7, 71.3, 69.66, 69.61, 69.56, 69.1, 68.9, 49.8, 48.6, 41.7, 38.61, 38.56, 37.6, 34.0, 31.0, 22.1 14.1. **LRMS (ESI) (m/z)**: calculated for $[M+H]^+$ ($C_{47}H_{56}O_9N_{15}$) requires 986.4, found: 986.3.

3.3 Synthesis of 1V-alkyne and 2V-alkyne

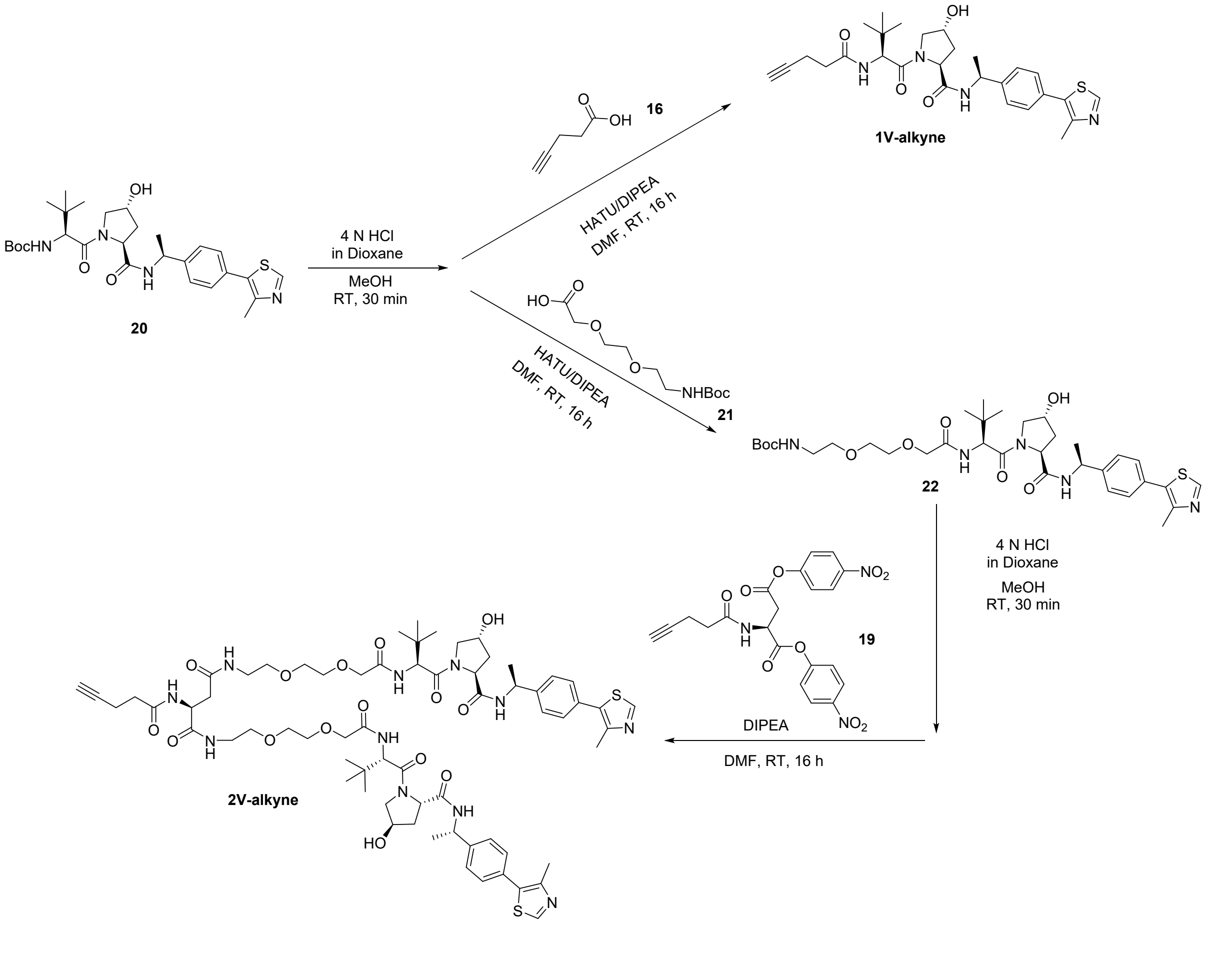

Scheme 3, synthetic route to 1V-alkyne and 2V-alkyne.

<u>Synthesis of 1V-alkyne</u>

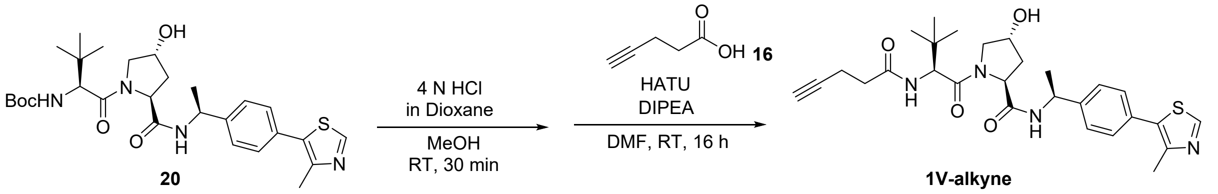

In the first step, compound **20** (prepared according to the known literature: *J. Med. Chem.* **2019**, 62, 1420-1442.) (82 mg, 0.15 mmol, 1.0 equiv.) was dissolved in MeOH (5 mL), to which 4 N HCl in dioxane (1.0 mL) was added. The mixture stirred at room temperature for 30 min, and the solvent was removed by rotavapor. The crude amine salt was used in the next step without further purification.

In the second step, acid **16** (15 mg, 0.15 mmol, 1.0 equiv.) and DIPEA (133 µl, 0.76 mmol, 5.0 equiv.) were first dissolved in DMF (2.0 mL). Then at room temperature, HATU (58 mg, 0.15 mmol, 1.0 equiv.) was added, the mixture was stirred for 10 min. Then the amine salt prepared above was added, and the mixture was stirred at room temperature for 12 h. Afterwards, DMF

was first removed under vacuum. The mixture was dissolved by DCM and washed by Sat. NH_4Cl (aq.) and brine. The organic phase was then dried over Na_2SO_4, filtered, and concentrated. The resulting residue was purified by silica gel column chromatography (DCM/MeOH = 20:1) to deliver compound **1V-alkyne** (52 mg, 67% yield) as a colorless solid.

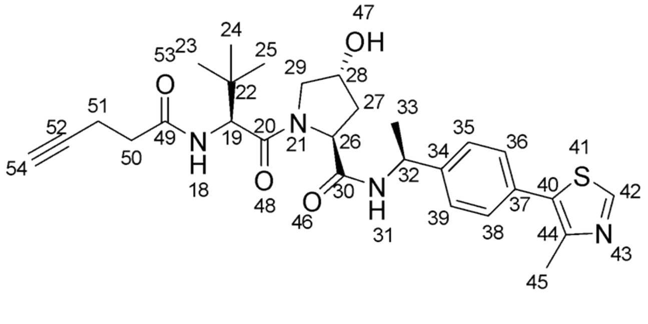

^{1}H NMR (400 MHz, Chloroform-*d*) δ 8.68 (s, 1H, **H42**), 7.42-7.35 (m, 5H, including **H35, H36, H38, H39** and **NH-31**), 6.48 (d, *J* = 8.5 Hz, 1H, **NH-18**), 5.08 (p, *J* = 7.1 Hz, 1H, **H32**), 4.71 (t, *J* = 7.9 Hz, 1H, **H26**), 4.57 (d, *J* = 8.6 Hz, 1H, **H19**), 4.54-4.51(m, 1H, **H28**), 4.10 (d, *J* = 11.2 Hz, 1H, **H29**), 3.61 (dd, *J* = 11.4, 3.7 Hz, 1H, **H29**), 3.21-3.13 (m, 4H), 2.53 (s, 3H, **H45**), 2.51-2.48 (m, 2H, including **H27**), 2.11-2.03 (m, 1H, **H27**), 2.03 (t, *J* = 2.5 Hz, 1H, **H54**), 1.48-1.46 (m, 3H, **H33**), 1.06 (s, 9H, **H23, H24, H25**). **^{13}C NMR (100 MHz, Chloroform-*d*)** δ 172.1, 171.9, 169.7, 150.5 (**C42**), 148.6, 143.2, 131.0, 129.7 (**C35 or C36**), 126.6 (**C35 or C36**), 82.9 (**C52**), 70.2 (**C28**), 69.9 (**C54**), 58.6 (**C26**), 58.0 (**C19**), 56.8 (**C29**), 49.0 (**C32**), 46.8, 43.3, 35.5, 35.3, 35.2, 26.6 (**C23, C24, C25**), 22.3 (**C33**), 16.0 (**C45**). **LRMS (ESI) (m/z):** calculated for $[M+H]^+$ ($C_{28}H_{37}O_4N_4S_1$) requires 525.2, found: 525.2.

Synthesis of compound 22

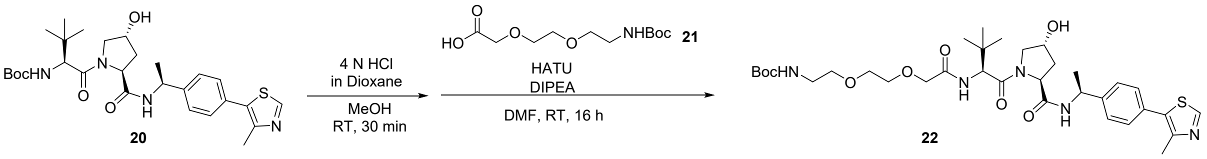

In the first step, compound **20** (prepared according to the known literature: *J. Med. Chem.* **2019**, 62, 1420-1442.) (251 mg, 0.46 mmol, 1.2 equiv.) was dissolved in MeOH (5 mL), to which 4 N HCl in dioxane (1.0 mL) was added. The mixture stirred at room temperature for 30 min, and the solvent was removed by rotavapor. The crude amine salt was used in the next step without further purification.

In the second step, commercially available compound **21** (100 mg, 0.38 mmol, 1.0 equiv.) and DIPEA (133 μl, 0.76 mmol, 2.0 equiv.) were first dissolved in DMF (2.0 mL). Then at room temperature, HATU (160 mg, 0.42 mmol, 1.1 equiv.) was added, the mixture was stirred for 10 min. Then the amine salt prepared above was added, and the mixture was stirred at room temperature for 12 h. Afterwards, DMF was first removed under vacuum. The mixture was dissolved by DCM and washed by Sat. NH_4Cl (aq.) and brine. The organic phase was then dried over Na_2SO_4, filtered, and concentrated. The resulting residue was purified by silica gel

column chromatography (DCM/MeOH = 20:1 to 15:1) to deliver a major compound **22** (195 mg, 74% yield) as a colorless foam. **Note:** compound **22** was mixture of rotamers with ratio of 1.5/1. Its further characterization was delayed in the next step where the Boc group was removed. However, **LRMS** clearly showed compound **22** was obtained. **LRMS (ESI) (m/z):** calculated for $[M+H]^+$ ($C_{34}H_{52}O_8N_5S_1$) requires 690.4, found: 690.3.

Synthesis of compound 22’

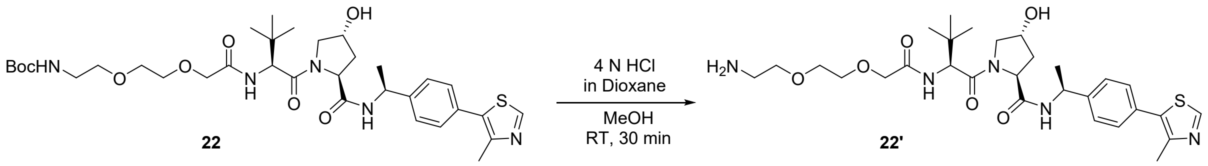

Compound **22** (150 mg, 0.22 mmol, 1.0 equiv.) was dissolved in MeOH (5.0 mL) and then 4 M HCl (2.0 mL) was added. The mixture was stirred at room temperature for 30 min. Then the mixture was concentrated, and the residue was purified by silica gel column chromatography ($DCM/MeOH/NH_4OH$ = 10:1:0 to 100:20:1) to deliver compound **22’** (115 mg, 89% yield) as a pale white foam.

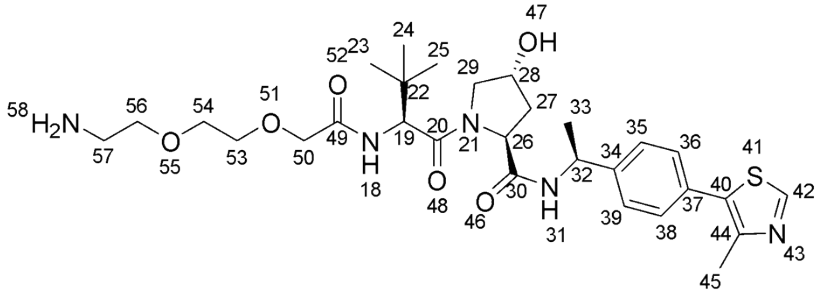

Note: product **22’** exists as a 4.5/1 ratio of rotamers at C19-NH18 bond. Only the major rotamer is characterized here. **^{1}H NMR (400 MHz, Chloroform-*d*)** δ 8.66 (s, 1H, **H42**), 7.71 (d, *J* = 7.9 Hz, 1H), 7.48 (d, *J* = 8.8 Hz, 1H), 7.41-7.35 (m, 4H), 5.13-5.06 (m, 1H, **H32**), 4.73 (td, *J* = 8.4, 2.3 Hz, 1H, **H26**), 4.66 (d, *J* = 9.3 Hz, 1H, **H19**), 4.44 (brd, 1H, **H28**), 4.11 (dd, *J* = 15.7, 2.3 Hz, 1H), 4.02-3.95 (m, 2H, **including H29**), 3.80-3.37 (m, 10H, **including H29**), 2.97-2.87 (m, 2H), 2.52 (brd, 3H, **H45**), 2.36-2.24 (m, 1H, **H27**), 2.15-2.09 (m, 1H, **H27**), 1.49 (dd, *J* = 7.0, 1.4 Hz, 3H, **H33**), 1.03 (brd, 9H, **H23, H24, H25**). **^{13}C NMR (100 MHz, Chloroform-*d*)** δ 171.5, 170.7, 170.1, 150.4, 148.6, 143.6, 131.8, 130.9, 129.6, 126.6, 71.6, 71.0, 69.9, 58.8 (**C26**), 57.1 (**C29**), 56.8 (**C19**), 48.9 (**C32**), 41.1, 36.5 (**C27**), 36.3, 35.7, 31.0, 29.8, 26.6 (**C23, C24, C25**), 22.5 (**C33**), 16.2 (**C45**). **LRMS (ESI) (m/z):** calculated for $[M+H]^+$ ($C_{29}H_{44}O_6N_5S_1$) requires 590.3, found: 590.2.

Synthesis of compound 2V-alkyne

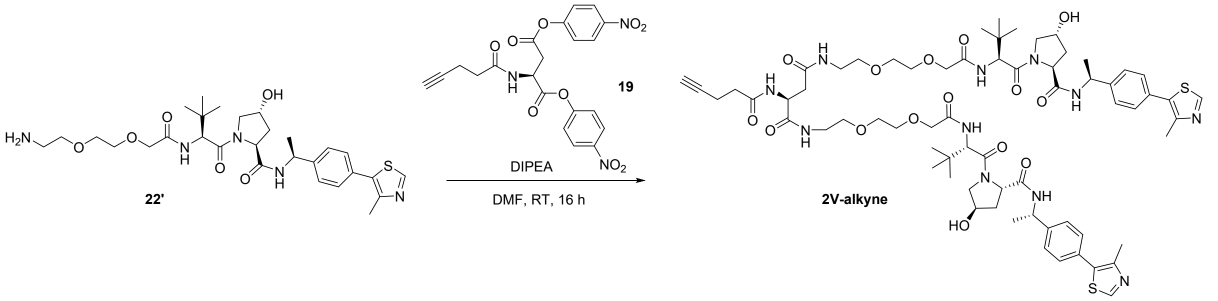

Compound **19** (26 mg, 0.054 mmol, 1.0 equiv.), compound **22'** (70 mg, 0.12 mmol, 2.2 equiv.) and DIPEA (28 μl, 0.16 mmol, 3.0 equiv.) were mixed in DMF (2.0 mL). The mixture was stirred at room temperature for 12 h. Afterwards, DMFwas removed under vacuum, and the crude residue was purified by preparative TLC (DCM/MeOH = 10:1) to deliver compound **2V-alkyne** (38 mg, 52% yield) as a colorless solid.

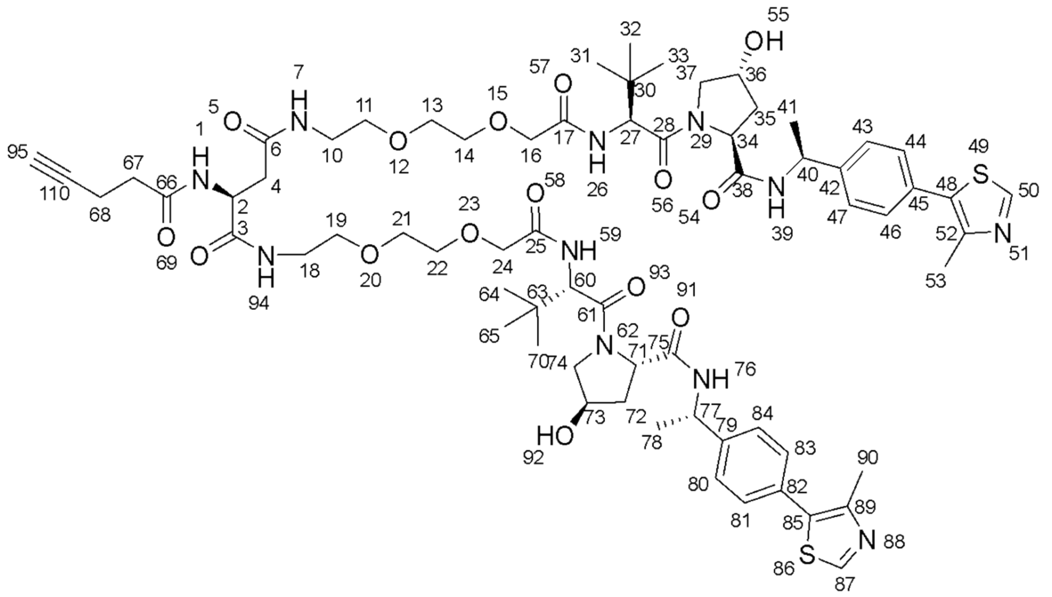

^{1}H NMR (400 MHz, Chloroform-*d*) δ 9.02 (dd, *J* = 7.3, 3.6 Hz, 1H), 8.69-8.67 (m, 3H), 8.34 (d, *J* = 6.5 Hz, 1H), 7.73 (dd, *J* = 9.5, 4.0 Hz, 2H), 7.48 -7.43 (m, 3H), 7.39-7.31 (m, 6H), 7.17 (t, *J* = 5.6 Hz, 1H), 5.44 (ddd, *J* = 11.7, 9.0, 2.3 Hz, 1H), 5.20 (app. p, *J* = 7.1 Hz, 1H), 5.04 (dd, *J* = 8.5, 6.8 Hz, 1H), 4.92 (t, *J* = 6.8 Hz, 1H), 4.88-4.81 (m, 3H), 4.49 (brd, 1H), 4.33 (t, *J* = 3.9 Hz, 1H), 4.16-4.04 (m, 4H), 4.00-3.91 (m, 2H), 3.89-3.80 (m, 4H), 3.77-3.55 (m, 11H), 3.47-3.42 (m, 1H), 3.13-2.97 (m, 4H), 2.63-2.58 (m, 2H), 2.52 (s, 3H), 2.51 (s, 3H), 2.45-2.41 (m, 2H), 2.37-2.34 (m, 2H), 2.33-2.29 (m, 1H), 2.25-2.19 (m, 1H), 2.11-2.01 (m, 2H), 1.95 (t, *J* = 2.5 Hz, 1H, **H95**), 1.42-1.37 (m, 6H, **H41, H78**), 1.08 (s, 9H), 1.07 (s, 9H). **^{13}C NMR (100 MHz, Chloroform-*d*)** δ 173.1, 171.4, 171.3, 171.22, 171.20, 170.8, 170.7, 169.9, 169.6, 144.30, 144.27, 130.8, 130.4, 129.6, 129.4, 126.8, 126.2, 82.8 (**C110**), 71.5, 71.3, 71.2, 70.1 (**C95**), 69.95, 69.85, 69.77, 69.67, 69.4, 68.8, 67.9, 65.1, 59.9, 58.2, 58.0, 57.7, 56.7, 56.6,

50.5, 49.3, 48.4, 45.9, 40.0, 39.4, 38.84, 38.79, 38.4, 36.8, 35.6, 34.9, 34.6, 32.0, 29.83, 29.77, 29.75, 29.71, 29.65, 29.5, 29.4, 28.7, 27.2, 26.65 (**C31, C32, C33**), 26.58 (**C64, C65, C70**), 26.0, 22.8, 22.6 (**41**), 22.2 (**C78**), 16.2 (**C53, C90**). **LRMS (ESI) (m/z):** calculated for $[M+H]^+$ ($C_{67}H_{94}O_{15}N_{11}S_2$) requires 1356.6, found: 1356.4.

3.4 Building block synthesis for control PROTACSs with similar linker length to that of dual-ligand PROTACs

<u>**Synthesis of compound 24**</u>

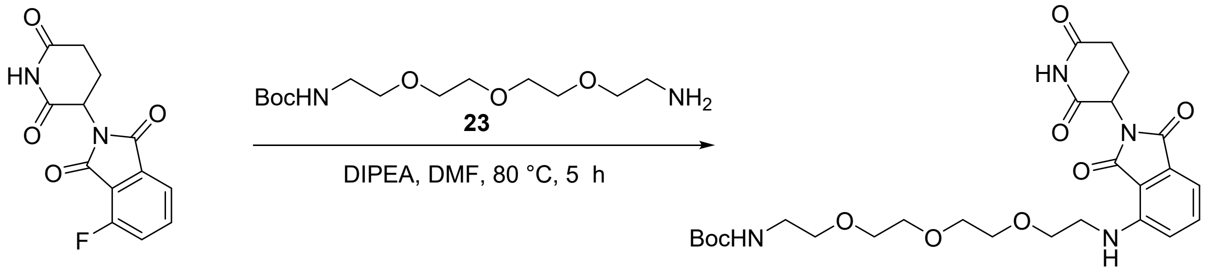

Compound **12** (276 mg, 1.00 mmol, 1.0 equiv.), compound **23** (351mg, 1.20 mmol, 1.2 equiv.) and DIPEA (349 µl, 2.00 mmol, 2.0 equiv.) were mixed in DMF (5.0 mL) and then stirred at 80 °C for 5 hours. After cooling, the reaction mixture was concentrated and directly purified by silica gel column chromatography (hexane/ethyl acetate = 1/1, then DCM/MeOH = 30/1) to give compound **24** (227 mg, 41% yield) as a greenish oil.

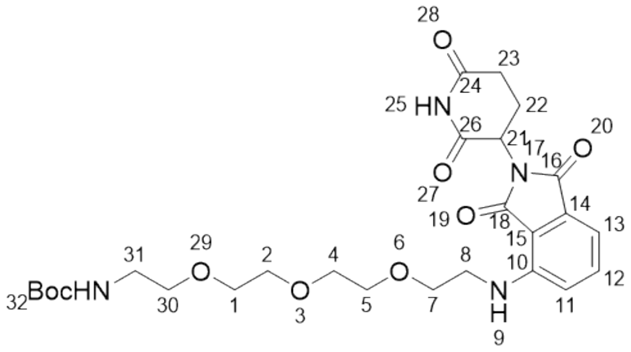

^{1}H NMR (400 MHz, DMSO-d_6) δ 11.09 (s, 1H, **H25**), 7.58 (dd, J = 8.6, 7.1 Hz, 1H, **H12**), 7.14 (d, J = 8.6 Hz, 1H, **H11**), 7.04 (d, J = 7.0 Hz, 1H, **H13**), 6.72 (t, J = 5.8 Hz, 1H, **Boc-NH**), 6.60 (t, J = 5.8 Hz, 1H, **NH9**), 5.05 (dd, J = 12.9, 5.4 Hz, 1H, **H21**), 3.62 (t, J = 5.4 Hz, 2H), 3.59-3.43 (m, 10H), 3.37-3.34 (m, 2H, **immersed in water peak**), 3.05 (q, J = 6.0 Hz, 2H, **H31**), 2.95-2.82 (m, 1H, **H23**), 2.64-2.52 (m, 2H, **including H22, H23**), 2.08-1.98 (m, 1H, **H22**), 1.36 (s, 9H, **Boc-H**). **^{13}C NMR (100 MHz, DMSO-d_6)** δ 172.8, 170.1, 168.9, 167.3, 155.6, 146.4, 136.2, 132.1, 117.4, 110.7, 109.2, 77.6, 69.80, 69.78, 69.5, 69.2, 68.9, 48.6, 41.7, 31.0, 28.2, 22.2. **LRMS (ESI) (m/z)**: calculated for $[M-H]^-$ ($C_{26}H_{35}O_9N_4$) requires 547.2, found: 547.2.

<u>**Synthesis of compound 26**</u>

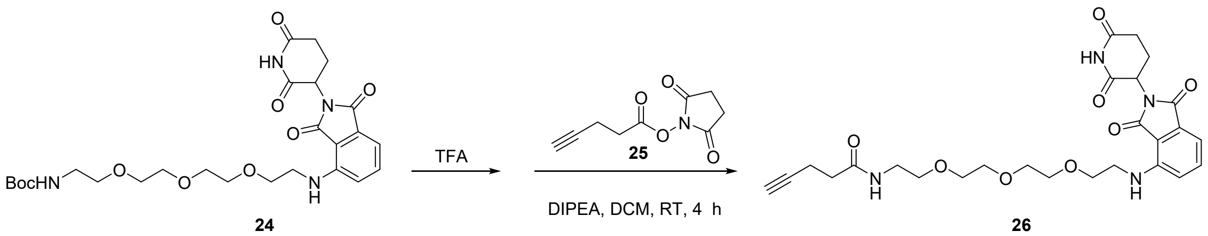

In the first step, compound **24** (227 mg, 0.41 mmol) was dissolved in DCM/TFA (5.0 mL/2.0 mL) and stirred at room temperature for 30 min. The solvent was removed by rotavapor and the crude product was used in the next step without further purification.

In the second step, compound **25** (14 mg, 0.072 mmol, 1.2 equiv.), crude product from above (33 mg, 0.060 mmol, 1.0 equiv.) and DIPEA (10 μl, 0.06 mmol, 10.0 equiv.) was mixed in DCM (3.0 mL). The mixture was stirred at room temperature for 16 h. Afterwards, DCM was removed under vacuum, and the crude residue was purified by preparative TLC plate (DCM/MeOH = 10:1) to deliver compound **26** (24 mg, 76% yield) as a yellowish foam.

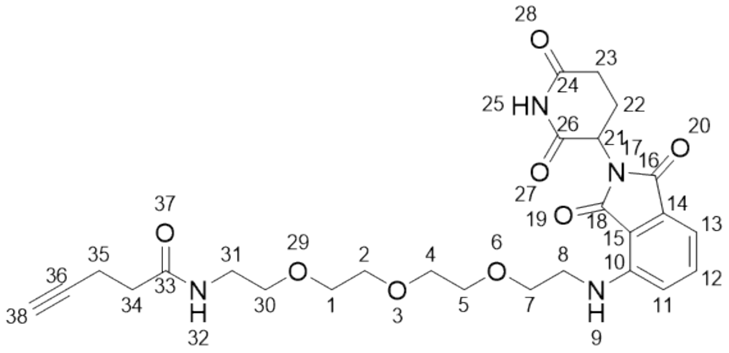

^{1}H NMR (400 MHz, Chloroform-*d*) δ 8.59 (s, 1H, **H25**), 7.48 (dd, *J* = 8.5, 7.1 Hz, 1H, **H12**), 7.09 (d, *J* = 7.1 Hz, 1H, **H13**), 6.90 (d, *J* = 8.6 Hz, 1H, **H11**), 6.47 (s, 1H), 6.31 (s, 1H), 4.91 (dd, *J* = 11.9, 5.5 Hz, 1H, **H21**), 3.71 (t, *J* = 5.4 Hz, 2H), 3.66 (s, 4H), 3.65-3.58 (m, 4H), 3.55 (dd, *J* = 5.5, 4.6 Hz, 2H), 3.50-3.40 (m, 4H), 2.91-2.81 (m, 1H, **H23**), 2.81-2.67 (m, 2H, including **H22, H23**), 2.54-2.45 (m, 2H, **H35**), 2.43-2.34 (m, 2H, **H34**), 2.15-2.07 (m, 1H, **H22**), 2.00 (t, *J* = 2.6 Hz, 1H, **H38**). **^{13}C NMR (100 MHz, Chloroform-*d*)** δ 171.5, 171.2, 169.4, 168.7, 167.7, 146.9, 136.2, 132.6, 116.9, 111.8, 110.4, 83.2 (**C36**), 70.9, 70.7, 70.6, 70.3, 69.9, 69.6, 69.4 (**C38**), 49.0 (**C21**), 42.5, 39.4, 35.3 (**C34**), 31.5, 31.0, 22.9, 14.9 (**C35**). **LRMS (ESI) (m/z)**: calculated for $[M-H]^-$ ($C_{26}H_{31}O_9N_4$) requires 527.2, found: 527.3.

<u>**Synthesis of compound 27**</u>

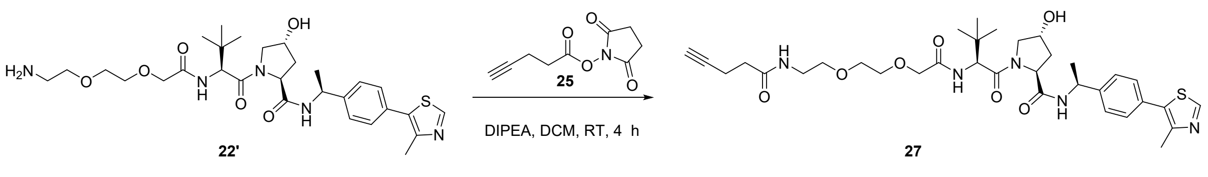

compound **22'** (33 mg, 0.056 mmol, 1.0 equiv.), compound **25** (13 mg, 0.067 mmol, 1.2 equiv.) and DIPEA (20 µl, 0.12 mmol, 2.1 equiv.) was mixed in DCM (3.0 mL). The mixture was stirred at room temperature for 4 h. Afterwards, DCM was removed under vacuum, and the crude residue was purified by preparative TLC plate (DCM/MeOH = 10:1) to deliver compound **27** (30 mg, 80% yield) as a white foam.

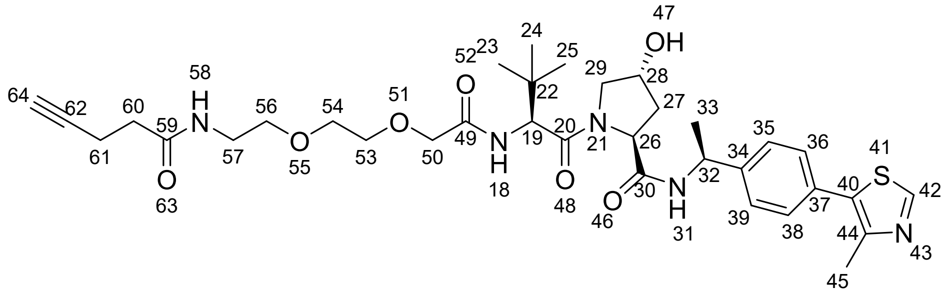

^{1}H NMR (400 MHz, Chloroform-*d*) δ 8.70 (s, 1H, **H42**), 7.51 (d, *J* = 9.4 Hz, 1H), 7.42-7.37 (m, 2H), 7.34 (d, *J* = 8.3 Hz, 2H), 7.09 (d, *J* = 7.7 Hz, 1H), 7.03 (d, *J* = 6.2 Hz, 1H), 5.09 (p, *J* = 6.9 Hz, 1H, **H32**), 4.74-4.63 (m, 2H), 4.55-4.47 (m, 1H), 4.01 (d, *J* = 8.6 Hz, 2H), 3.95 (d, *J* = 11.1 Hz, 1H), 3.75-3.46 (m, 8H), 3.41-3.30 (m, 1H), 2.52-2.51 (m, 8H), 2.40 (ddd, *J* = 13.0, 7.8, 4.8 Hz, 1H), 2.11 (ddt, *J* = 13.2, 8.1, 2.0 Hz, 1H), 1.48 (d, *J* = 6.9 Hz, 3H), 1.05 (s, 9H). **^{13}C NMR (100 MHz, Chloroform-*d*)** δ 171.7, 171.3, 170.7, 170.0, 150.6, 148.4, 143.2, 131.0, 129.7, 126.5, 71.5, 70.5, 70.0, 69.4, 58.9, 57.2, 56.9, 49.0, 39.4, 36.2, 35.2, 31.1, 21.6, 22.2, 16.1, 15.0. **LRMS (ESI) (m/z):** calculated for $[M+H]^+$ ($C_{34}H_{48}O_7N_5S_1$) requires 670.3, found: 670.3.

<u>Synthesis of compound 28</u>

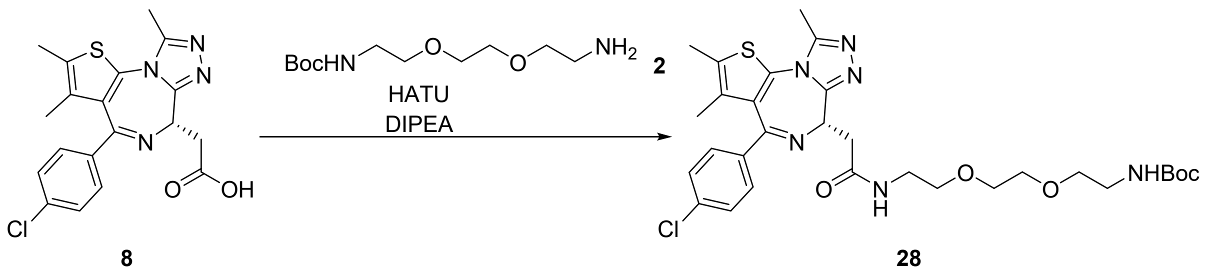

JQ1 acid **8** (100 mg, 0.25 mmol, 1.0 equiv.) and DIPEA (87 µl, 0.50 mmol, 2.0 equiv.) were mixed in DMF (1.0 mL) and then HATU (104 mg, 0.27 mmol, 1.1 equiv.) was added. The mixture was stirred at room temperature for 5 min. Afterwards, commercially available compound **2** (80 mg, 0.32 mmol, 1.3 equiv.) was added. The mixture was stirred at room temperature for 12 h. Then DMF was removed by rotovapor and the crude reaction mixture was diluted by DCM, washed by 1 M HCl (aq.) and then brine. The organic phase was then dried over Na_2SO_4, filtered, and concentrated. The crude mixture was then purified by silica

gel column chromatography (DCM/MeOH = 15:1) to give **28** (157 mg, 91% yield) as a colorless foam.

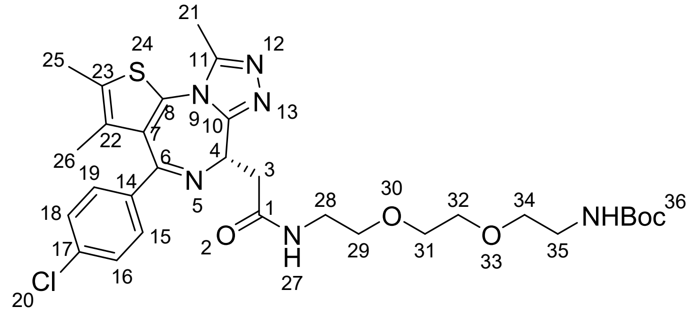

^{1}H NMR (400 MHz, Chloroform-*d*) δ 7.39 (d, *J* = 8.5 Hz, 2H, **Ar-H**), 7.31 (d, *J* = 8.7 Hz, 2H, **Ar-H**), 6.93 (s, 1H), 5.27 (s, 1H), 4.62 (t, *J* = 6.9 Hz, 1H, **H4**), 3.61 (s, 4H), 3.59-3.52 (m, 4H), 3.51-3.47 (m, 2H), 3.40-3.22 (m, 4H), 2.65 (s, 3H, **Ar-CH3**), 2.38 (s, 3H, **Ar-CH3**), 1.65 (s, 3H, **Ar-CH3**), 1.41 (s, 9H). **^{13}C NMR (100 MHz, Chloroform-*d*)** δ 170.7, 164.0, 156.2, 155.7, 150.0, 136.9, 136.7, 132.2, 131.0, 130.5, 130.0, 128.8, 70.3, 69.9, 55.6, 54.5, 45.1, 43.6, 40.4, 39.5, 39.3, 14.5, 13.2. **LRMS (ESI) (m/z)**: calculated for $[M+H]^+$ ($C_{30}H_{40}O_5N_6Cl_1S_1$) requires 631.2, found: 631.2.

<u>Synthesis of compound 29</u>

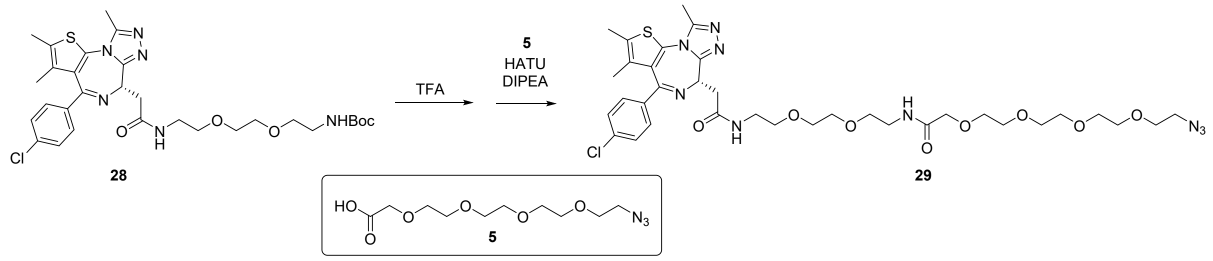

In the first step, compound **28** (150 mg, 0.24 mmol) was dissolved in DCM/TFA (5.0 mL/2.0 mL) and stirred at room temperature for 30 min. The solvent was removed by rotavapor and half of the crude product (0.12 mmol) was used in the next step without further purification.

In the second step, compound **5** (285 uL (0.5 M in tert-butyl methyl ether), 0.14 mmol, 1.2 equiv.), and DIPEA (10 µl, 0.06 mmol, 10.0 equiv.) was mixed in DMF (2.0 mL), then HATU (54 mg, 0.14 mmol, 1.2 equiv.) was added. The mixture was first stirred at room temperature for 5 min and then crude product from the above step (0.12 mol, 1.0 equiv.) and DIPEA (100 uL, 0.60 mmol, 5.0 equiv.) were added. The mixture was stirred at room temperature for 16 h. Afterwards, DMF was removed under vacuum, and the crude residue was purified by silica gel column chromatography (DCM/MeOH = 15:1) to deliver compound **29** (64 mg, 48% yield) as a colorless foam.

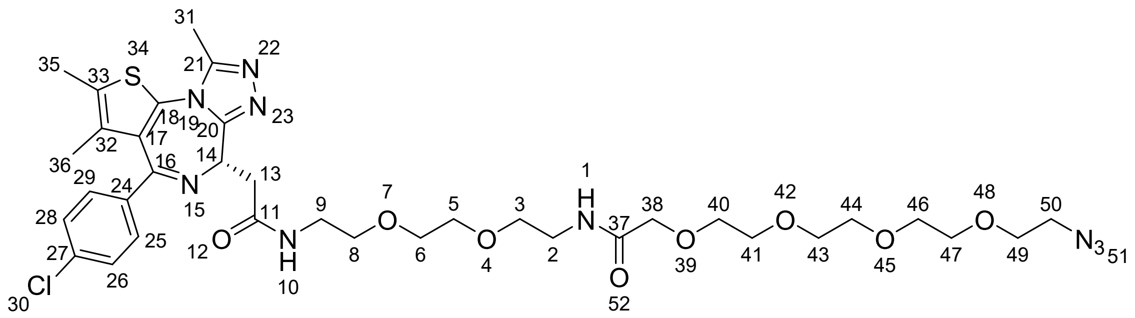

^{1}H NMR (400 MHz, Chloroform-*d*) δ 7.40 (d, *J* = 8.5 Hz, 2H, **Ar-H**), 7.32 (d, *J* = 8.7 Hz, 2H, **Ar-H**), 7.24 (d, *J* = 6.1 Hz, 1H), 6.93 (t, *J* = 5.5 Hz, 1H), 4.63 (t, *J* = 6.9 Hz, 1H, **H14**), 4.00 (s, 2H, **H38**), 3.65-3.47 (m, 28H), 3.38-3.32 (m, 2H), 2.65 (s, 3H, **Ar-CH3**), 2.39 (s, 3H, **Ar-CH3**), 1.66 (s, 3H, **Ar-CH3**). **^{13}C NMR (100 MHz, Chloroform-*d*)** δ 170.7, 170.3, 164.0, 155.8, 150.0, 136.9, 136.7, 131.0, 130.9, 130.6, 130.0, 128.8, 71.0, 70.8, 70.69, 70.66, 70.61, 70.5, 70.3, 70.1, 70.0, 69.9, 55.4, 54.5, 50.8, 43.5, 39.5, 39.2, 38.7, 18.7, 17.3, 14.5, 13.2. **LRMS (ESI) (m/z)**: calculated for $[M+H]^+$ ($C_{35}H_{49}O_8N_9Cl_1S_1$) requires 790.3, found: 790.4.

Note: the following click reaction to construct the PROTACs is the same as described in section 3.5.

3.5 Synthesis of BET PROTACs

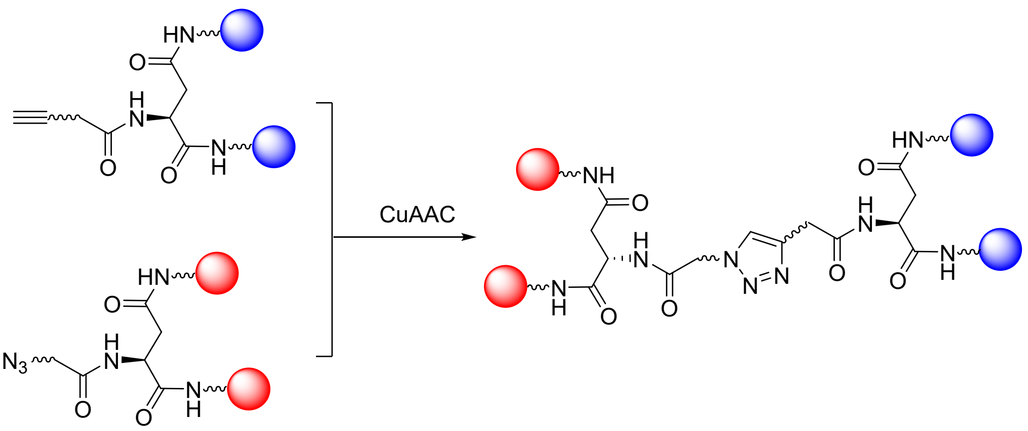

Scheme 4, general procedure for synthesis of dual-ligand PROTACs.

Note: The CuAAC click reaction was adapted from the literature: *J. Org. Chem.*, **2011**, *76*, 6832-6836. Detailed procedures are described below:

1. **Preparation of the stock solution of DIPEA/AcOH in DCM:** AcOH (114 μl, 2.0 mmol) and DIPEA (348 μl, 2.0 mmol) was sequentially added to the dry DCM (2.0 mL), the stock solution was then stored at -20 °C.
2. **Activation of the CuBr:** CuBr (10 g) was added to AcOH (5.0 mL), the mixture was then stirred at room temperature for 6 hours till the CuBr became pale white. Then the

suspension was filtered off, the CuBr was washed by acetone and dried under vacuum. The activated CuBr appears pale white while the oxidized CuBr normally appears light greenish.

3. **Running the CuAAC reaction:** The corresponding azdie (0.005 mmol, 1.0 equiv.) and the corresponding alkyne (0.0055 mmol, 1.0 equiv.) were first dissolved in DCM (1.0 mL) in a 2 mL glass vial. Then 50 μl of the solution of DIPEA/AcOH in dry DCM (prepared as above) was added to the above mixture, followed by the activated CuBr (5.0 mg, 0.035 mmol, 7.0 equiv.) (activated as above). The mixture was then capped well and stirred at room temperature for 2 to 12 hours. Afterwards, the reaction mixture was directly purified by preparative TLC plate to deliver the desired product.

Note: Due to the complicated structure and limited amount of product obtained, the final product was characterized by LCMS.

LCMS conditions:

Equipment: Agilent 1100 series HPLC with DAD detector and Agilent G1956B MS detector equipped with ESI ionization source.

Column: Phenomenex Kinetex C18, 150 x 4.6 mm, 5μm particle size at 35°C.

Flow 1.5 mL/min; eluent A: 5mM NH_4OAc in H_2O; eluent B: CH_3CN using the following gradient:

**Time [min]**	**Eluent B [%]**
0	0
0.5	0
6.5	100
8.5	100
8.7	0
10	0

Characterization of 2J1P

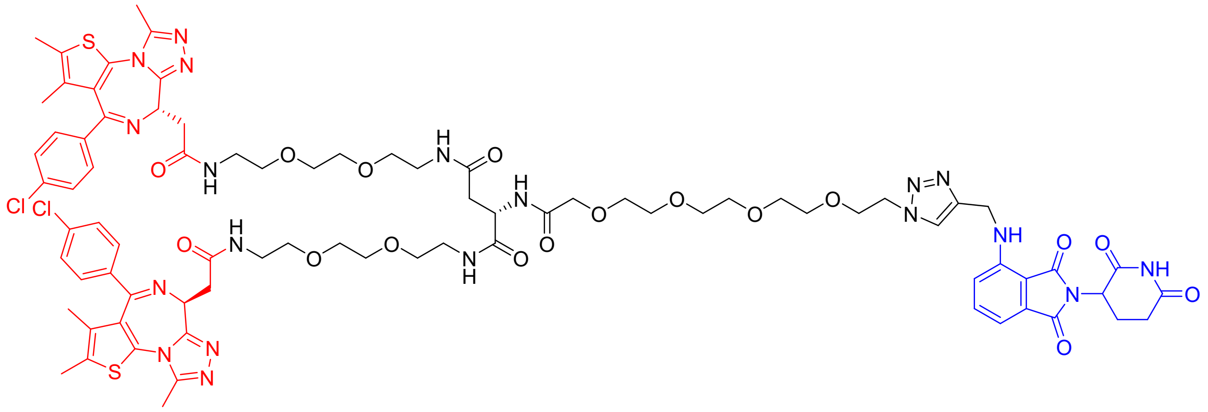

Chemical Formula: $C_{80}H_{95}Cl_2N_{19}O_{17}S_2$

3.8 mg, yield: 62%. Yellowish solid. Eluent for running preparative TLC: DCM/MeOH/NH_4OH = 70/10/0.5. LCMS retention time: 5.87 min. **LRMS (ESI) (m/z):** calculated for $[M+H]^+$ ($C_{80}H_{96}Cl_2O_{17}N_{19}S_2$) requires 1728.6, found: 1728.3.

LCMS spectrum:

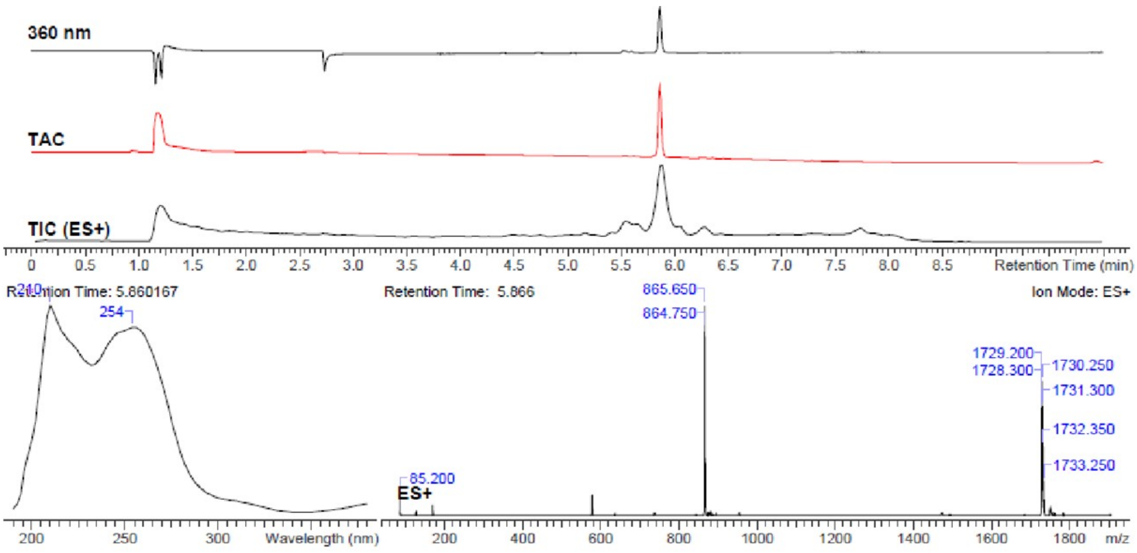

Characterization of 1J2P

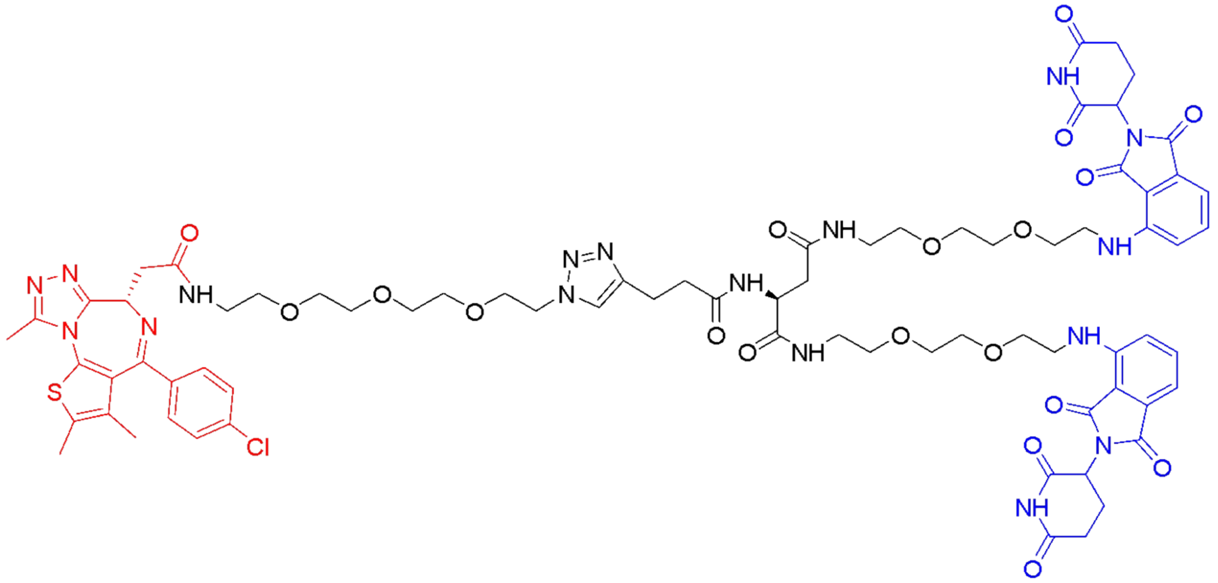

Chemical Formula: $C_{74}H_{88}ClN_{17}O_{19}S$

4.7 mg, 34% yield. Eluent for running preparative TLC: DCM/MeOH = 10/1. Yellowish solid. LCMS retention time: 5.60 min. **LRMS (ESI) (m/z):** calculated for $[M+H]^+$ ($C_{74}H_{89}Cl_{21}O_{19}N_{17}S_1$) requires 1586.6, found: 1586.2.

LCMS spectrum:

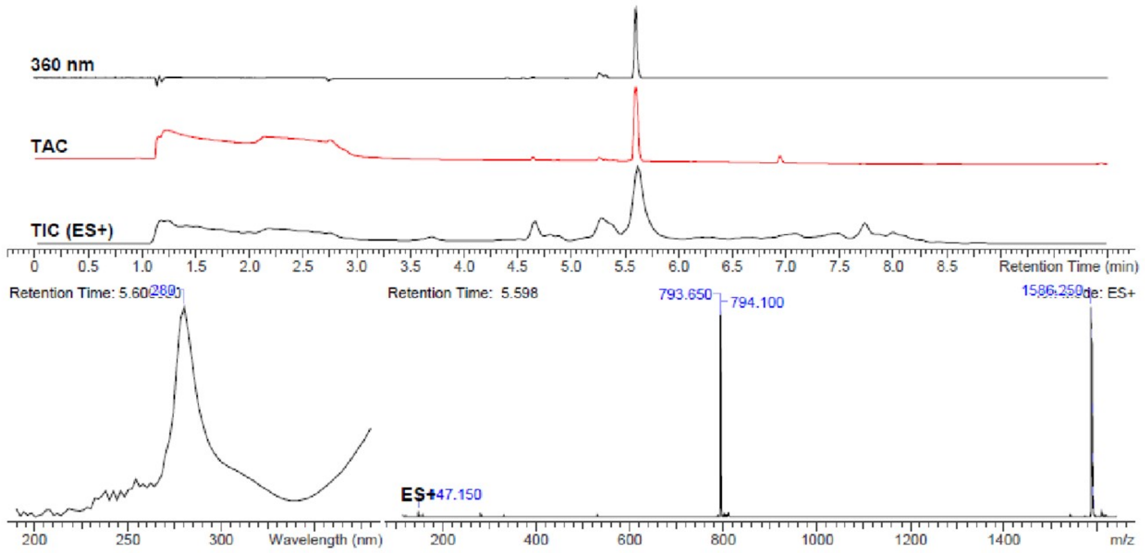

Characterization of 2J2P

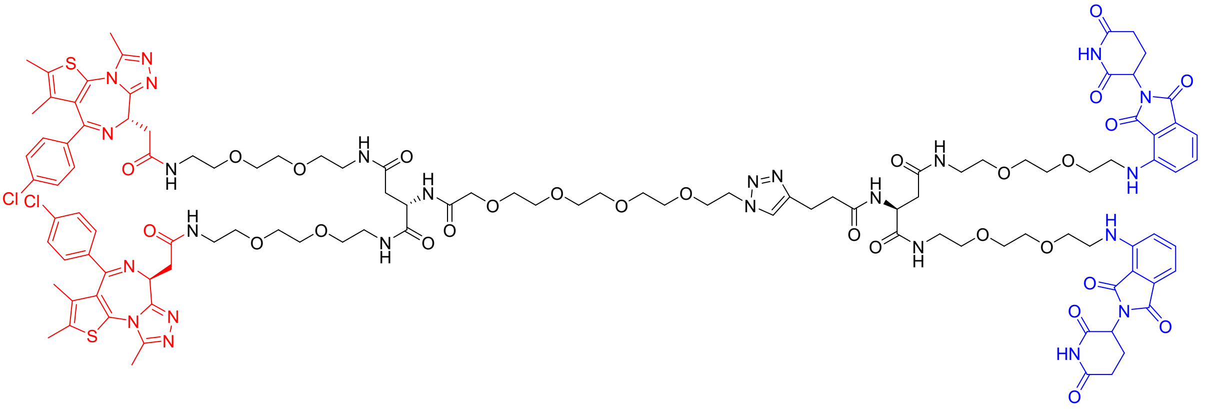

Chemical Formula: $C_{111}H_{137}Cl_2N_{25}O_{28}S_2$

3.5 mg, 58% yield. Yellowish solid. Eluent for running preparative TLC: DCM/MeOH/NH_4OH = 70/10/0.5. Retention time: 5.85 min. **LRMS (ESI) (m/z):** calculated for $[M+2H]^{2+}$ ($C_{111}H_{139}Cl_2O_{28}N_{25}S_2$) requires 1202.4, found: 1202.7.

LCMS spectrum:

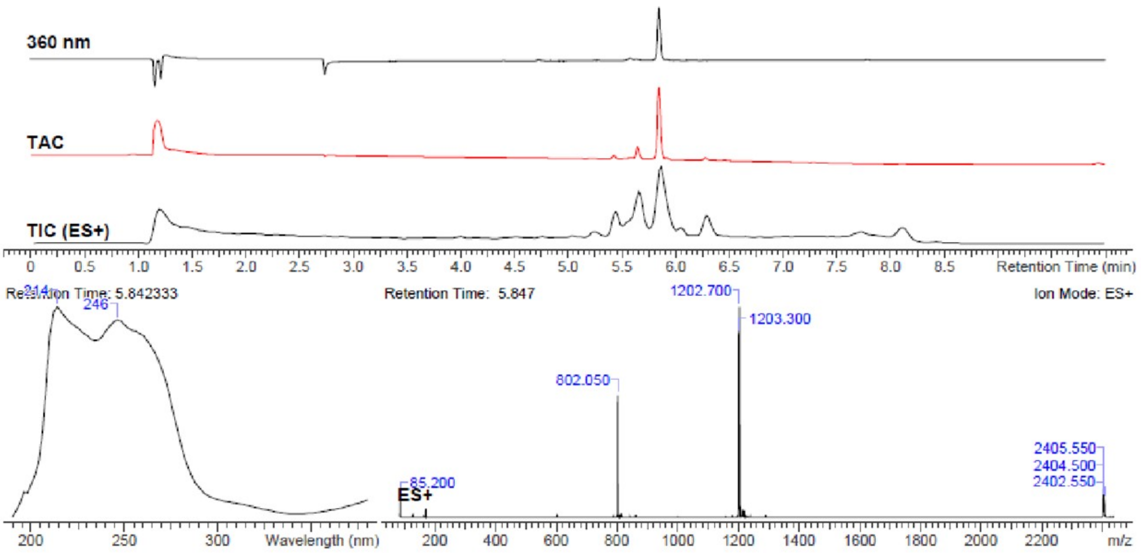

Characterization of 2J1V

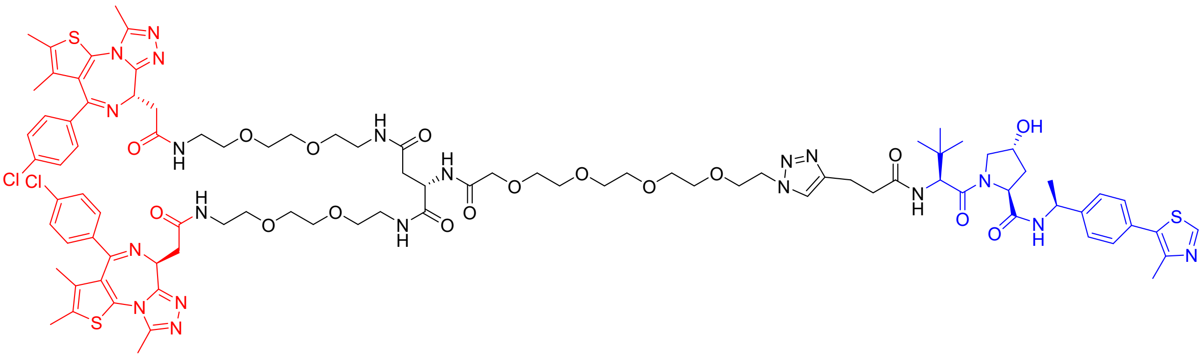

Chemical Formula: $C_{92}H_{118}Cl_2N_{20}O_{17}S_3$

4.5 mg, 42% yield. Colorless solid. Eluent for running preparative TLC: DCM/MeOH = 7/1. LCMS retention time: 5.92 min. **LRMS (ESI) (m/z):** calculated for $[M+2H]^{2+}$ ($C_{93}H_{120}Cl_2O_{17}N_{20}S_3$) requires 971.4, found: 971.2.

LCMS spectrum:

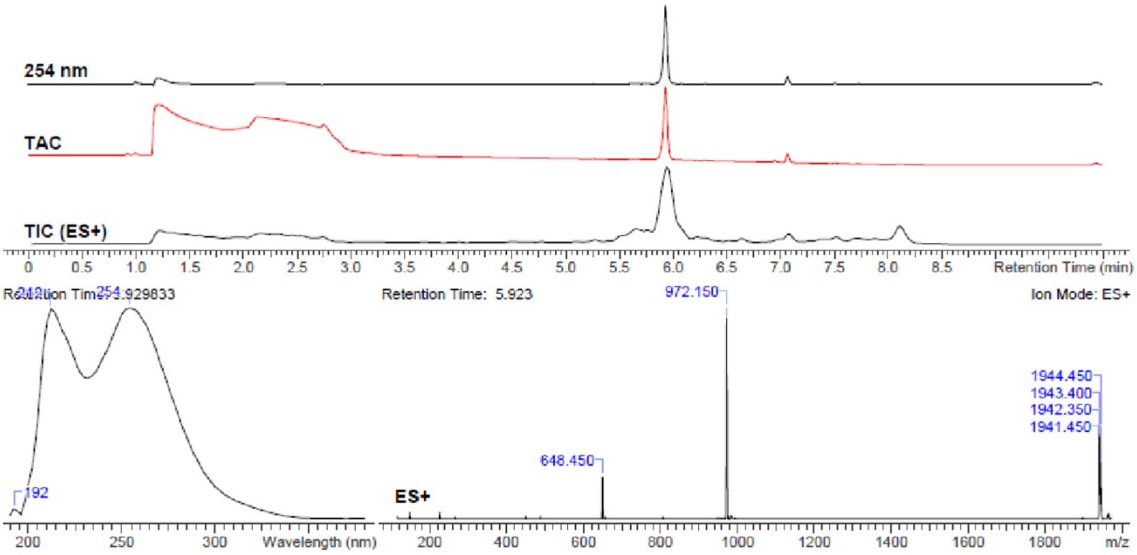

Characterization of 1J2V

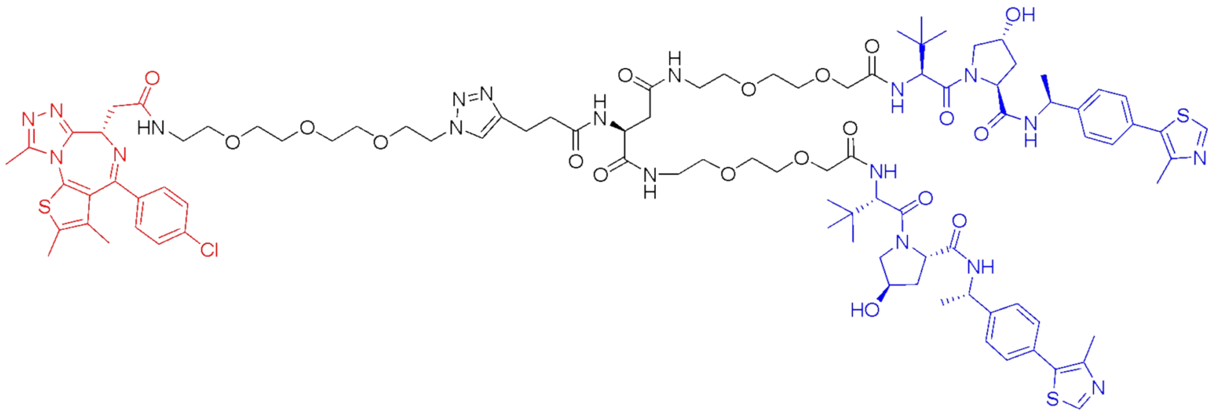

Chemical Formula: $C_{94}H_{126}ClN_{19}O_{19}S_3$

4.3 mg, 53% yield. Colorless solid. Eluent for running preparative TLC: DCM/MeOH = 7/1. LCMS retention time: 5.88 min. **LRMS (ESI) (m/z):** calculated for $[M+2H]^{2+}$ ($C_{94}H_{128}Cl_1O_{19}N_{19}S_3$) requires 979.4, found: 979.4.

LCMS spectrum:

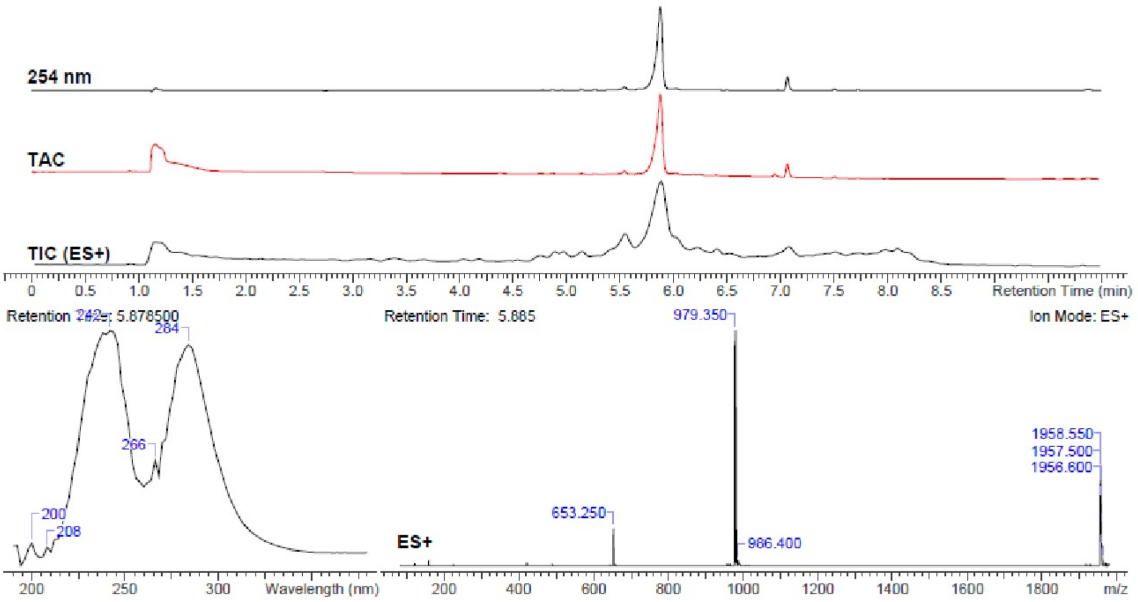

Characterization of 2J2V

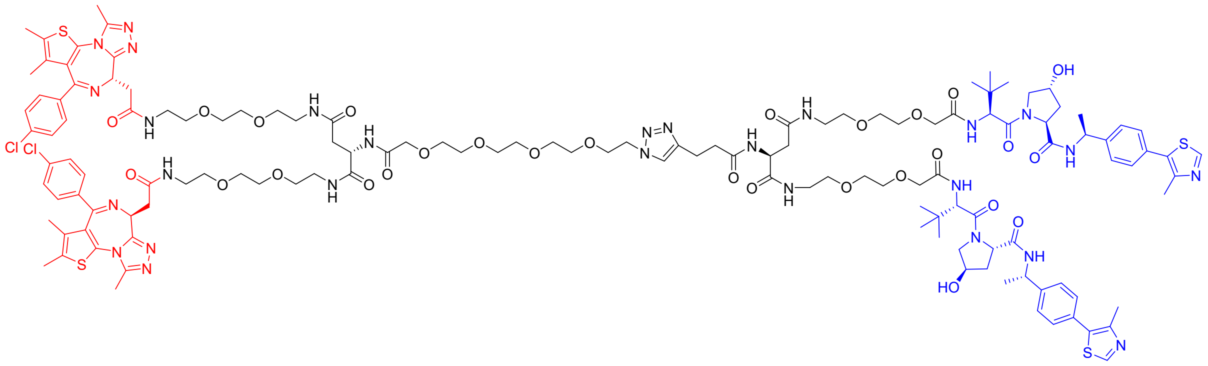

Chemical Formula: $C_{131}H_{175}Cl_2N_{27}O_{28}S_4$

3.7 mg, 24% yield. Colorless solid. Eluent for running preparative TLC: DCM/MeOH = 7/1. LCMS retention time: 6.02 min. **LRMS (ESI) (m/z):** calculated for $[M+2H]^{2+}$ ($C_{131}H_{177}Cl_2O_{28}N_{27}S_4$) requires 1387.6, found: 1387.9.

LCMS spectrum:

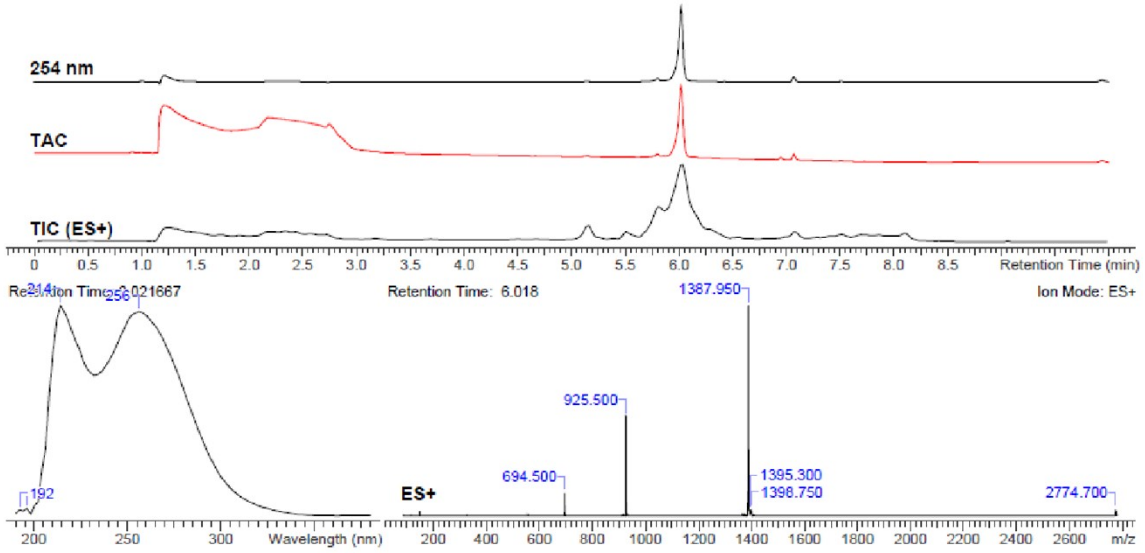

Characterization of 1J1P* (with similar linker length as 2J2P)

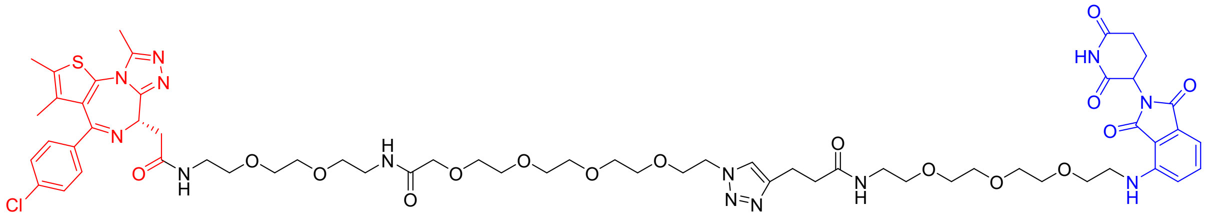

Chemical Formula: $C_{61}H_{80}ClN_{13}O_{16}S$

8.0 mg, 55% yield. Colorless solid. Eluent for running preparative TLC: DCM/MeOH = 8/1. LCMS retention time: 5.33 min. **LRMS (ESI) (m/z):** calculated for $[M+H]^+$ ($C_{61}H_{81}Cl_1O_{16}N_{13}S_1$) requires 1318.5, found: 1318.2.

LCMS spectrum:

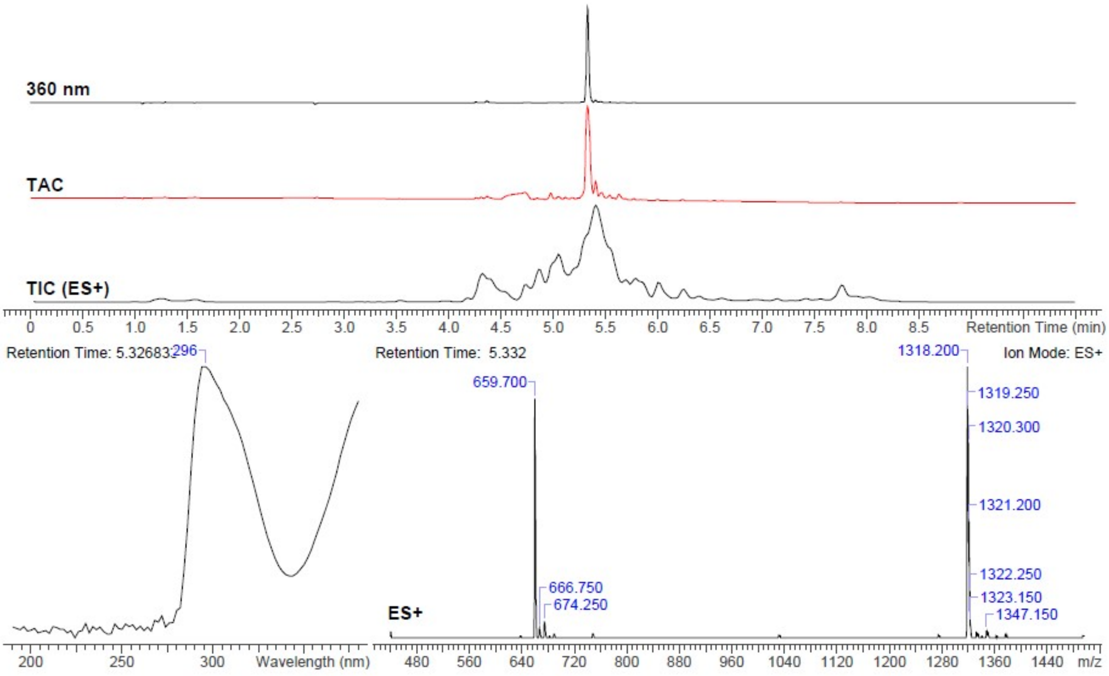

Characterization of 1J1V* (with similar linker length as 2J2V)

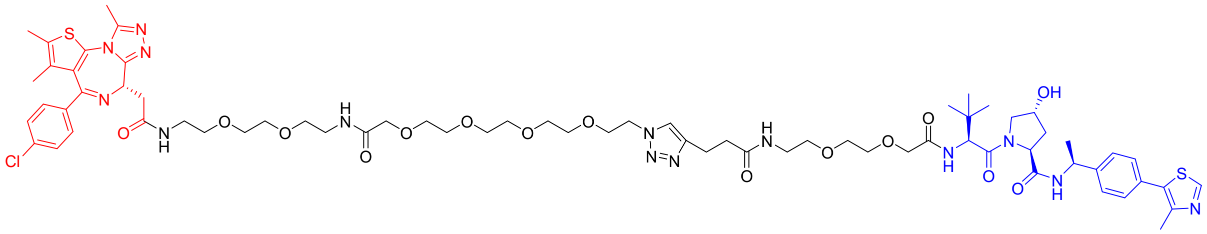

Chemical Formula: $C_{69}H_{95}ClN_{14}O_{15}S_2$

7.4 mg, 46% yield. Colorless solid. Eluent for running preparative TLC: DCM/MeOH = 8/1. LCMS retention time: 5.39 min. **LRMS (ESI) (m/z):** calculated for $[M+H]^+$ ($C_{69}H_{96}Cl_1O_{15}N_{14}S_2$) requires 1459.6, found: 1459.4.

LCMS spectrum:

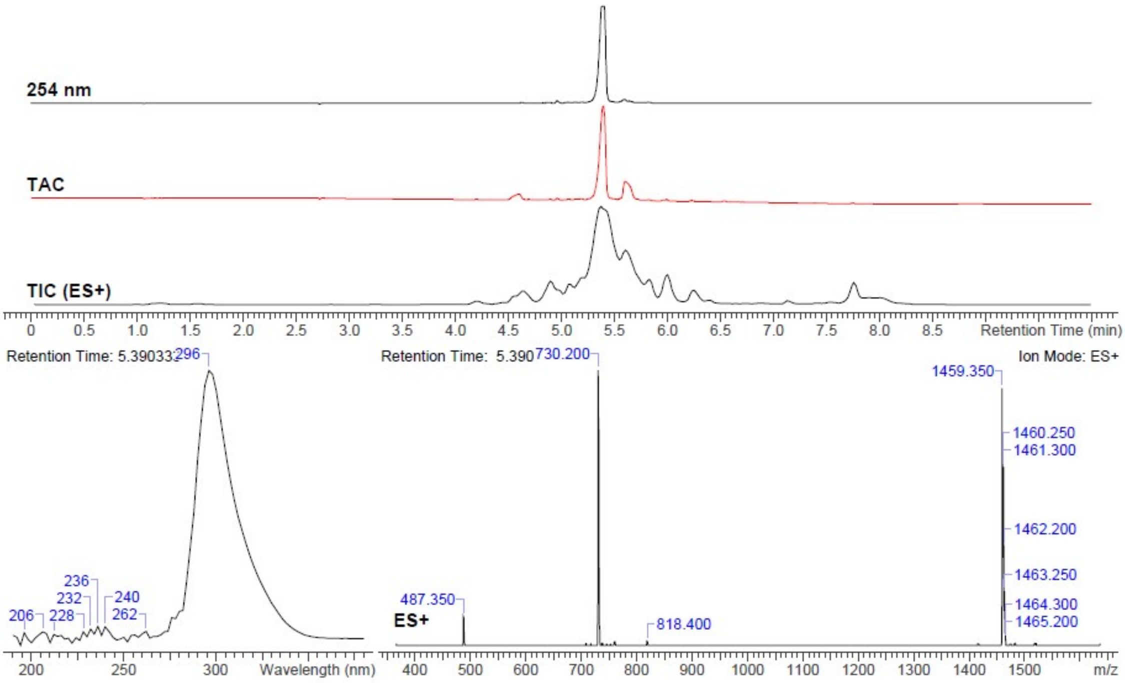

Characterization of 2J1P* (with similar linker length as 2J2P)

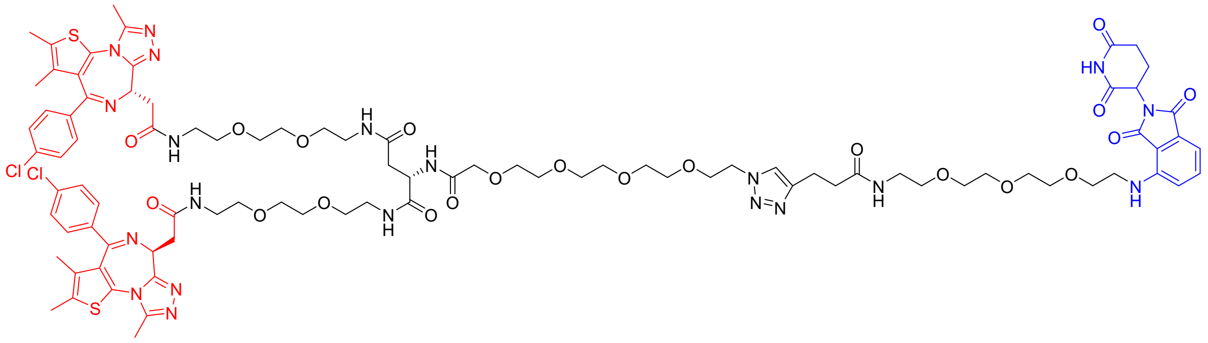

Chemical Formula: $C_{90}H_{114}Cl_2N_{20}O_{21}S_2$

6.4 mg, 40% yield. Colorless solid. Eluent for running preparative TLC: DCM/MeOH = 7/1. LCMS retention time: 5.78 min. **LRMS (ESI) (m/z):** calculated for $[M+2H]^{2+}$ ($C_{90}H_{116}Cl_2O_{21}N_{20}S_2$) requires 973.9, found: 974.1.

LCMS spectrum:

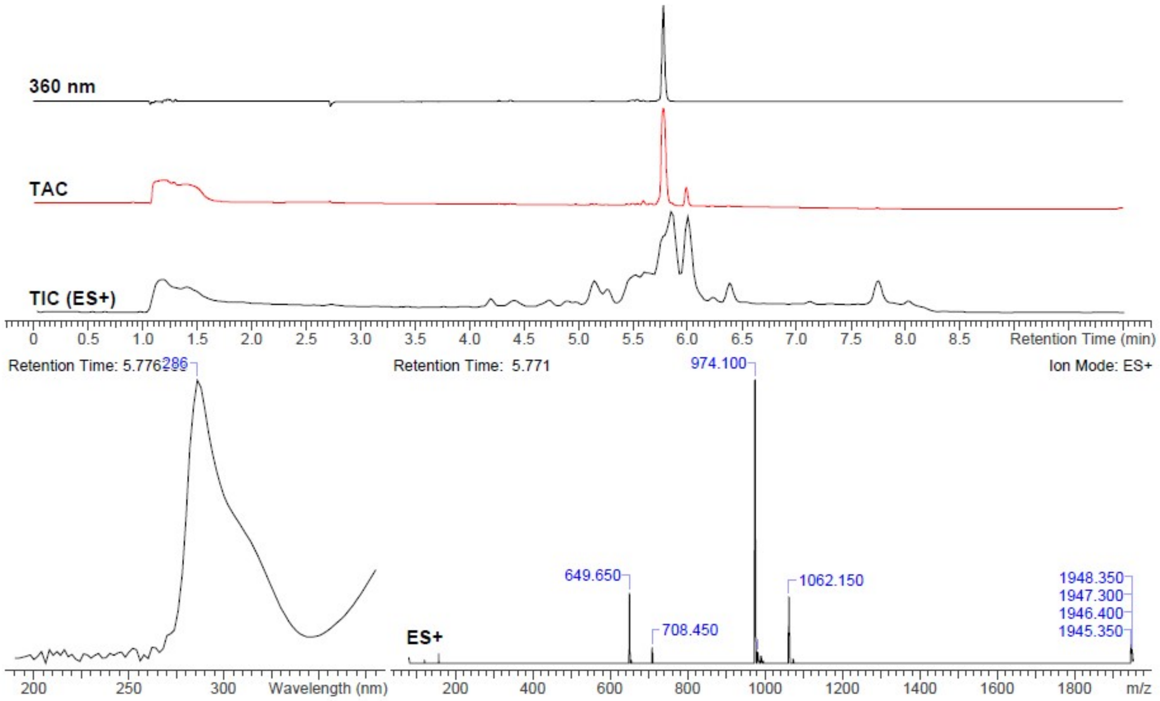

Characterization of 2J1V* (with similar linker length as 2J2P)

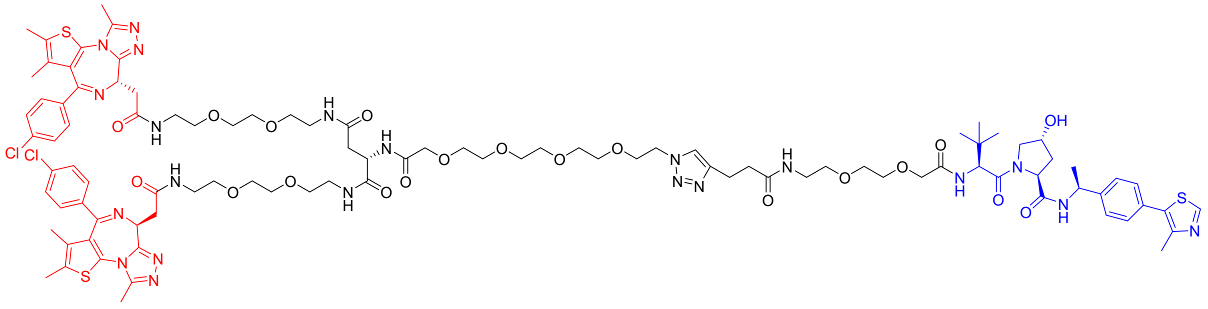

Chemical Formula: $C_{98}H_{129}Cl_2N_{21}O_{20}S_3$

7.2 mg, 54% yield. Colorless solid. Eluent for running preparative TLC: DCM/MeOH = 7/1. LCMS retention time: 5.84 min. **LRMS (ESI) (m/z):** calculated for $[M+2H]^{2+}$ ($C_{98}H_{131}Cl_2O_{20}N_{21}S_3$) requires 1044.4, found: 1044.7.

LCMS spectrum:

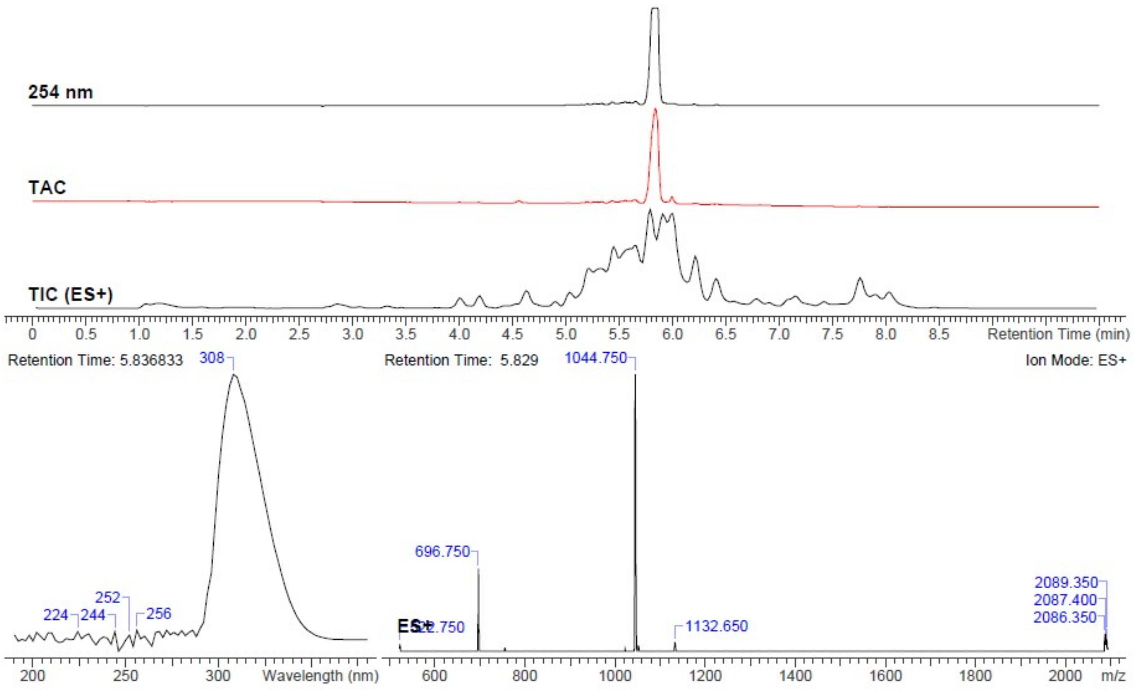

4. Biological experiments

4.1 Reagents

Primary antibodies used for western blotting included anti-human BRD2(1:2000, rabbit monoclonal, Abcam, ab139690), anti-human BRD3 (1:1000, rabbit monoclonal, Abcam, ab264420), anti-human BRD4 (E2A7X) (1:1000, rabbit monoclonal, Cell Signaling Technology, #13440), anti-human VHL (1:1000, rabbit polyclonal, Cell Signaling Technology, #68547), anti-human CRBN (D8H3S) (1:1000, rabbit polyclonal, Cell Signaling Technology, #71810), anti-human alpha Tubulin, clone B-5-1-2 (1:5000, mouse monoclonal, Merck Life Science, # 32-2500).Secondary antibodies used for western blot included: anti-mouse horseradish peroxidase-linked antibody (1:3000, sheep, GE Healthcare Life Sciences, #NA931V), and anti-rabbit horseradish peroxidase-linked antibody (1:8000, donkey, GE Healthcare Life Sciences, #NA934V). Reagent for degradation assay and compounds synthesis included:MG132 (MedChem Express, HY-13259), MZ1(MedChem Express, HY-107425), dBET1 (MedChem Express, HY-101838), lenalidomide (a gift from the lab of Prof. dr. Serge Van Calenbergh, Ghent University).

4.2 Cell lines and culture

HEK293, A549, SKOV-3, HCT116 cells (ATCC)were grown in DMEM (Gibco),and all cells were supplemented with 10% (v/v) fetal bovine serum (FBS) (Sigma), 100 mg/mL penicillin/streptomycin solution (Thermo Fisher Scientific) and maintained at 37 °C and 5% CO2 in a humidified atmosphere. All cells used were tested monthly using the Mycoalert Mycoplasma Detection Kit (cat. no. LT07-318, Lonza) to exclude mycoplasma contamination.

4.3 Degradation assays by Western blot

General method of Western blot: Lysates were sonicated and centrifuged at 4 °C at 20000 g for 15 min, and the supernatants were transferred to new tubes. Protein concentration of cell lysates was measured using the DC Protein Assay kit (BIO-RAD) following manufacturer's instructions. Cell lysates were mixed with reducing sample buffer (0.5 M Tris-HCl (pH 6.8), 43% glycerol, 9.2% SDS, 5% 2-mercaptoethanol, 5% bromophenol blue) and boiled for 5 min at 95°C. Proteins were separated by SDS–PAGE (SDS polyacrylamide gel electrophoresis) with 25–40 µg of total protein per well, transferred to 0.2-µm-pore nitrocellulose membranes (BIO-RAD), then blocked in 5%(w/v) milk in PBS and 0.5% Tween-20, and incubated overnight at 4°C with primary antibodies described in **section 4.1**. Secondary antibodies were added for 1h at room temperature after 3 times washing with blocking buffer. After 6 times of washing

with PBS (Thermo Fisher Scientific) and 0.5% Tween-20, Blots were detected using the Clarity Western ECL Substrate (BIO-RAD) and visualized on iBright CL 750 (Thermo Fisher Scientific) and images were analyzed using iBright Firmware 1.7.0.

4.3.1 Evaluation of the degradation capacity of PROTACs

HEK293 or A549 cells were seeded at 2 $\times 10^6$ cells per ml in six-well plates overnight. The following day, cells were treated with compounds at concentrations of 0.1 μM, 1 μM, and 10 μM, or an equivalent volume of DMSO as control group. After 4h treatment, cells were washed three times with PBS (Thermo Fisher Scientific) and 100-200 μl of Laemmli lysis buffer (0.125 M Tris–HCl [pH 6.8], 10% glycerol, 2.3% SDS) were added into each well. Cell lysates were collected for Western blotting.

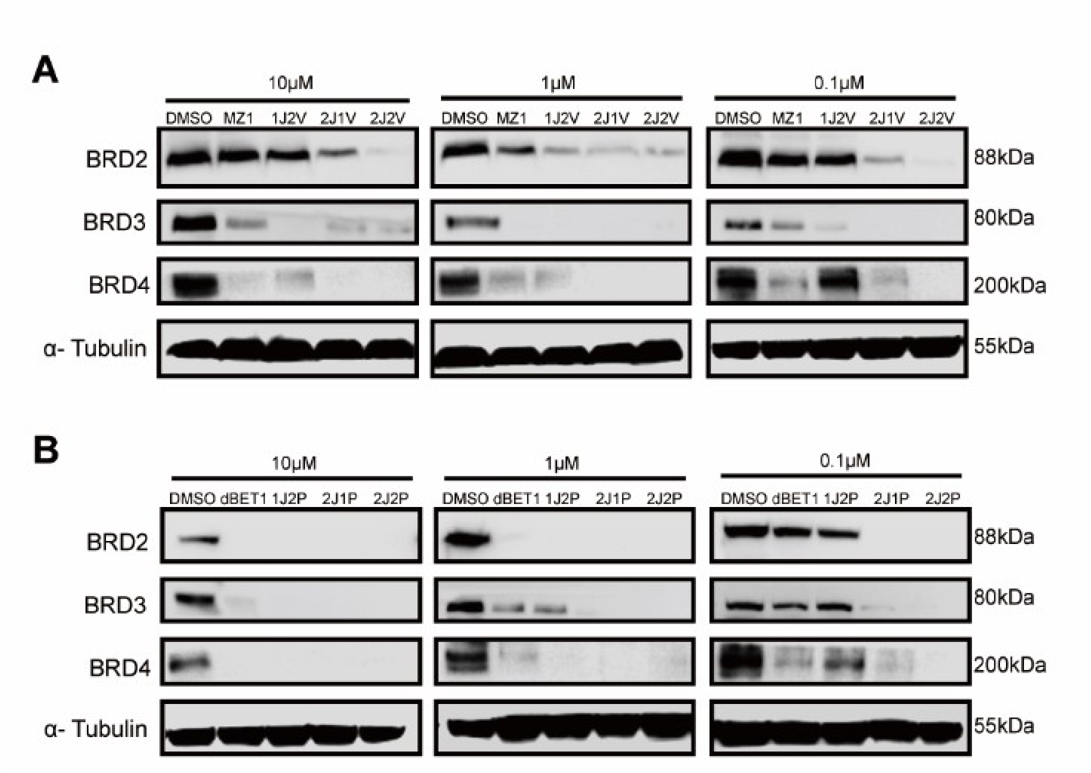

Figure S1 Dual-ligand PROTACs induce target protein degradation in A549 cells. A549 cells were treated with all synthesized PROTACs for 4h. Subsequently, cells were lysed and BRD2, BRD3 and BRD4 protein levels were measured by Western blot. **(A)** Representative image of BET protein degradation was screened for PROTACs recruiting the VHL E3 ligase and commercial MZ1 at a concentration of 0.1, 1, 10 μM, respectively (equivalent volume of DMSO as control group). **(B)** Representative image of BET protein degradation was screened for PROTACs recruiting the CRBN E3 ligase and commercial dBET1 at a concentration of 0.1, 1, 10 μM, respectively (equivalent volume of DMSO as control group).

4.3.2 Comparison of degradation capability of single and dual-ligand PROTACs.

HEK293 cells were seeded at 2×10^6 cells per ml in six-well plates overnight. The following day, cells were treated with **MZ1**/**2J2V** and **dBET1**/**2J2P** at concentrations from 10 μM to 0.001nM or an equivalent volume of DMSO as control group. After 4h treatment, cells were washed three times with PBS (Thermo Fisher Scientific) and 100 μl of Laemmli lysis buffer was added into each well. Cell lysates were collected for Western blotting.

4.3.3 Comparison of control PROTACs with similar linker length to that of dual-ligand PROTACs

HEK293 cells were seeded at 2×10^6 cells per ml in six-well plates overnight. The following day, cells were treated with **1J1V*/1J1P***, **2J1V*/2J1V/2J2V** and **2J1P*/2J1P/2J2P** at concentrations from 0.1μM to 1nM or an equivalent volume of DMSO as control group. After 4h treatment, cells were washed three times with PBS (Thermo Fisher Scientific) and 100 μl of Laemmli lysis buffer was added into each well. Cell lysates were collected for Western blotting.

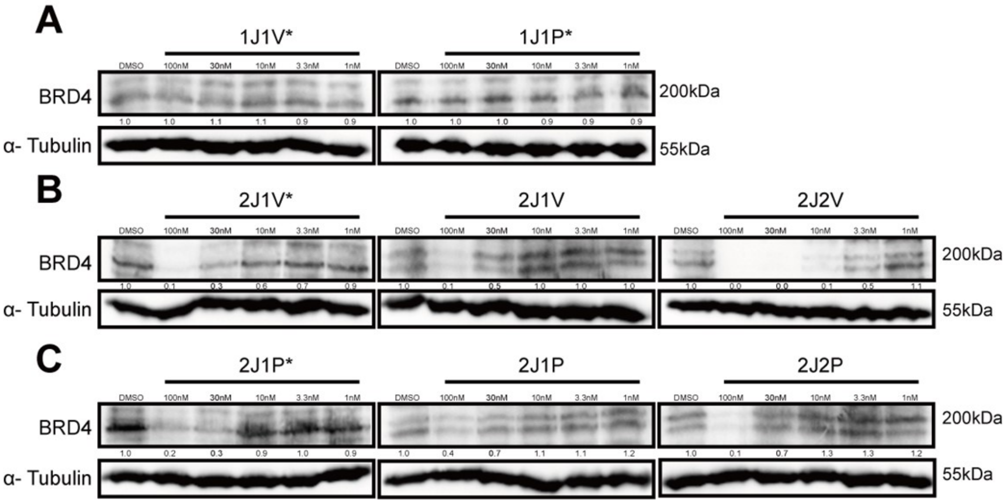

Figure S2 Dual-ligand PROTACs equipped with negative control CRBN ligands or with pomalidomide induce target protein degradation in HEK293 cells. HEK293 cells were treated with PROTACs for 4h. Subsequently, cells were lysed and BRD4 protein levels were measured by Western blot. **(A)** BRD4 protein degradation by 1J1V/1J1P was tested over a 0.1μM to 1nM concentration range (vehicle-only control contained DMSO). **(B)** BRD4 protein degradation by 2J1V*/2J1V/2J2V was tested over a 0.1μM to 1nM concentration range (vehicle-only control contained DMSO). **(C)** BRD4 protein degradation by 2J1P*/2J1P/2J2P was tested over a 0.1μM to 1nM concentration range (vehicle-only control contained DMSO). The numerical number is the relative BRD4 protein level compared to that of cells treated with DMSO normalized by the internal reference Tubulin.

4.3.4 Kinetic Assay

HEK293 were seeded at 2×10^6 cells per ml in six-well plates overnight. The following day, cells were treated with **2J2V**/**MZ1**(100 nM), **2J2P**/ **dBET1** (100 nM) and equivalent volumes

of DMSO as control group. At 4h, medium was removed and replaced with fresh medium. Cell lysates were collected after 4 h,12 h,24 h,36 h,60 h of treatment for Western blotting.

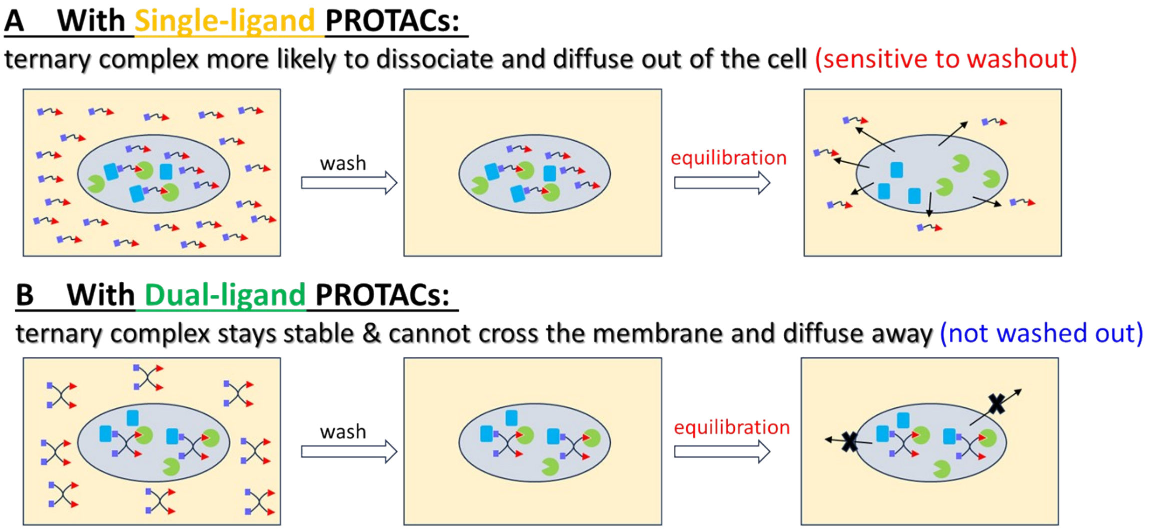

Figure S3 A schematic model to demonstrate the sustained protein degradation effect by dual-ligand PROTACs after the washout experiment. The PROTACs will stay both inside and outside the cell when they are added to the culture medium. However, the removal of the culture medium will also remove the PROTACs outside the cell. **(A)** In the case of single-ligand PROTACs, due to the removal of the PROTACs-containing culture medium, the PROTACs will diffuse from intracellular space to the extracellular space and result in the significant loss of intracellular PROTACs. **(B)** In the case of dual-ligand PROTACs, although the removal of the PROTACs-containing culture medium will drive the PROTACs to diffuse outward the cell, the dual-ligand PROTACs can still be entrapped inside the cell due to the stabilized ternary complex formation among dual-ligand PROTACs, E3 ligase and BET protein.

4.3.5 Rescue Assay

Proteosome-dependent experiment: HEK293 were seeded at 2×10^6 cells per ml in six-well plates overnight. The following day, cells were treated with or without proteasome inhibitor MG132 (50 μM). After 4 h, the medium was removed and replaced with fresh medium with 2J2V/MZ1(100 nM), 2J2P/ dBET1 (100 nM) and equivalent volumes of DMSO as control group. After another 4 h treatment, Cell lysates were collected for Western blotting.

CRBN ligase-engagement experiment: HEK293 were seeded at 2×10^6 cells per ml in six-well plates overnight. The following day, cells were treated with or without lenalidomide (10 μM). After 2 h, 2J2P (100 nM) or an equivalent volume of DMSO as control group was added to the medium without washout. After another 4 h treatment, Cell lysates were collected for Western blotting.

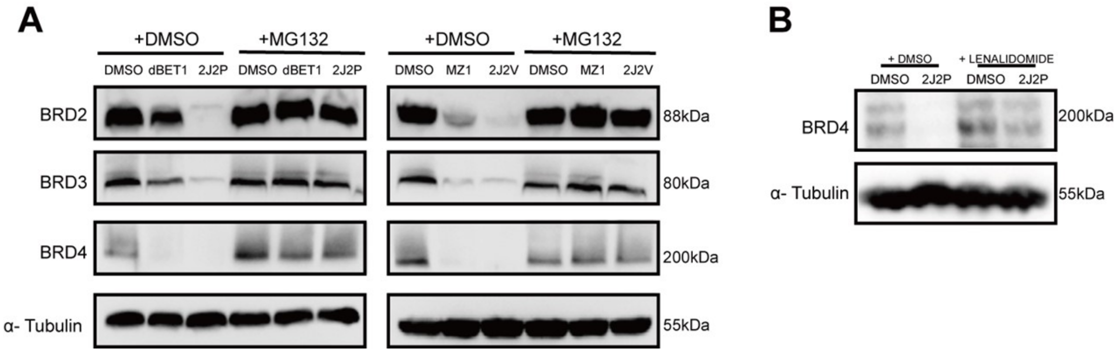

Figure S4 Dual-ligand PROTACs degrade protein in a proteasome and E3 ligase-dependent way. **(A)** HEK293 cells were preincubated with or without the proteasome inhibitor MG132 (50 μM) for 4 h, then the medium was washed and replaced with medium containing PROTACs. Representative image of BET protein degradation in HEK293 cells treated with PROTACs, both with and without the addition of MG132. (equivalent volume of DMSO as control group). Experiments were performed as three independent replicates. **(B)** HEK293 cells were preincubated with or without lenalidomide (10 μM) for 2 hours, followed by the addition of 2J2P(100nM) or an equivalent volume of DMSO for the control group. The representative image shows BRD4 protein degradation in HEK293 cells treated with 2J2P, with and without lenalidomide.

4.3.6 Evaluation of E3 ligase homo-degradation.

HEK293 were seeded at 2×10^6 cells per ml in six-well plates overnight. The following day, cells were treated with compounds or an equivalent volume of DMSO as the control group. After 4 h treatment, cells were washed three times with PBS (Thermo Fisher Scientific) and 100 μl of Laemmli lysis buffer were added into each well. Cell lysates were collected for Western blotting.

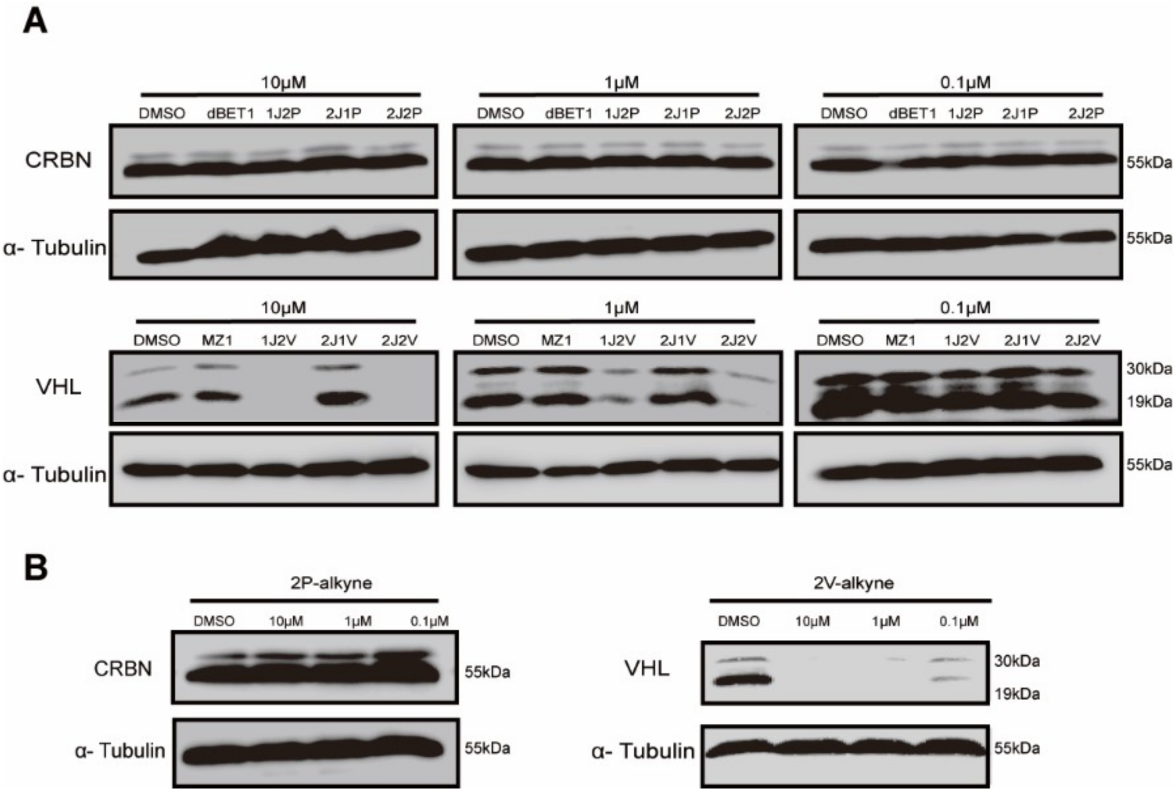

Figure S5 Homo-degradation effect in Dual-ligand PROTACs. HEK293 cells were treated with all synthesized PROTACs or 2V-alkyne/2P-alkyne for 4 hours. Subsequently, cell lysates were prepared and the protein levels of CRBN or VHL E3 ligase were assessed by Western blotting. (A) Representative image of CRBN or VHL E3 ligase homo-degradation was screened for all PROTACs and commercial single-ligand PROTACs at a concentration of 0.1, 1 and 10 μM, respectively (equivalent volume of DMSO as control group). (B) Representative image of CRBN or VHL E3 ligase homo-degradation was screened for 2P-alkyne or 2V-alkyne at a concentration of 0.1, 1 and 10 μM, respectively (equivalent volume of DMSO as control group). Experiments were performed as three independent replicates.

4.4 Cell viability assay on 2D cells cultures

HEK293, HCT116, A549,andSKOV-3 cells were seeded in 96-well tissue culture plates at a density of 2000-3000 cells per well in 100 μl of growth medium and incubated overnight at 37 °C with 5% CO2. The following day, the cells were treated with various concentrations of compounds or 0.05% DMSO for 48 h. After treatment, add 100 μl of Cell Titer-Glo® 2.0 Reagent (cat. no. G9243, Promega) in each well, then the 96-well plates were put on an orbital shaker for 10 min and then allow the plate to incubate at room temperature for 10-20min to stabilize the luminescent signal. Luminescence readout (Gen5 Data Analysis Software v3.08.01) was performed to record luminescence.

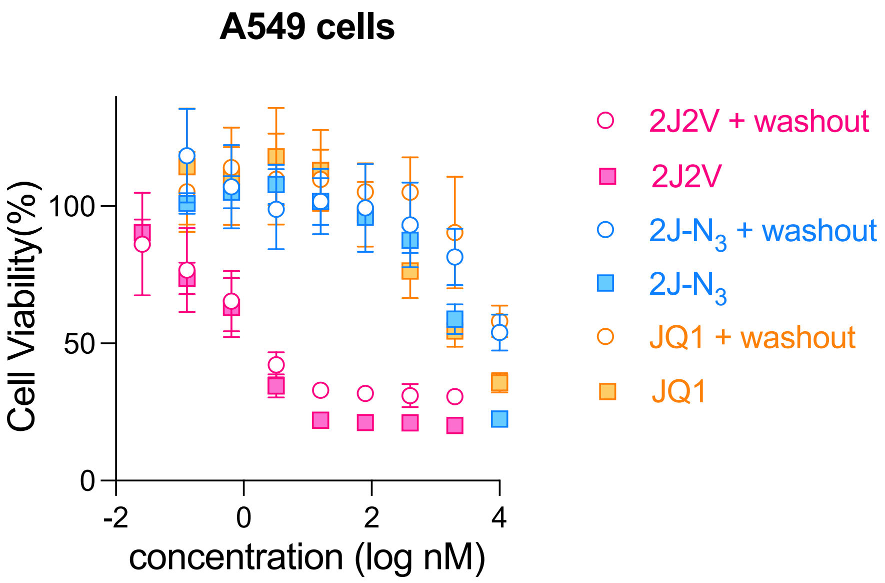

Figure S6 Dual-ligand PROTAC activity is not caused by enhanced BRD-inhibition by dual JQ1 display. A549 cells were treated with an escalating dose of 2J2V, 2J-N_3 and JQ1, respectively, for 8h. Subsequently, cells were either washed and cultured in fresh medium, or left untouched. After a total of 48h, cell viability was measured by MTT assay (n=6).

4.5 Cell viability assay on 3D cell cultures

The single cell suspension containing 2.5 $\times 10^4$ cells/ml of A549 or 2 $\times 10^4$ cells/ml of HCT116 were seeded into the U-shaped, 384-well ULA plates (cat. no. MS-9384UZ, S-bio), each well containing 80 μl single cell suspension. The 384-well ULA plates were sealed with Breath-Easy semipermeable tape (cat. no. BEM-1, Diversified Biotech) to prevent evaporation. The spheroids were put into incubator and then cultured at 37 °C and 10% CO2. After 48h of spheroids formation, the spheroids were treated with various concentrations of compounds or 0.05% DMSO. After 72h treatment, each spheroid in the culture medium was pipetted into white micro-96-well plates (cat. no. 236108, ThermoFisher) and an equal volume of Cell Titer-Glo 3D (cat. no. G9683, Promega) reagent was added. Luminescence readouts were the same as described above for 2D culture.

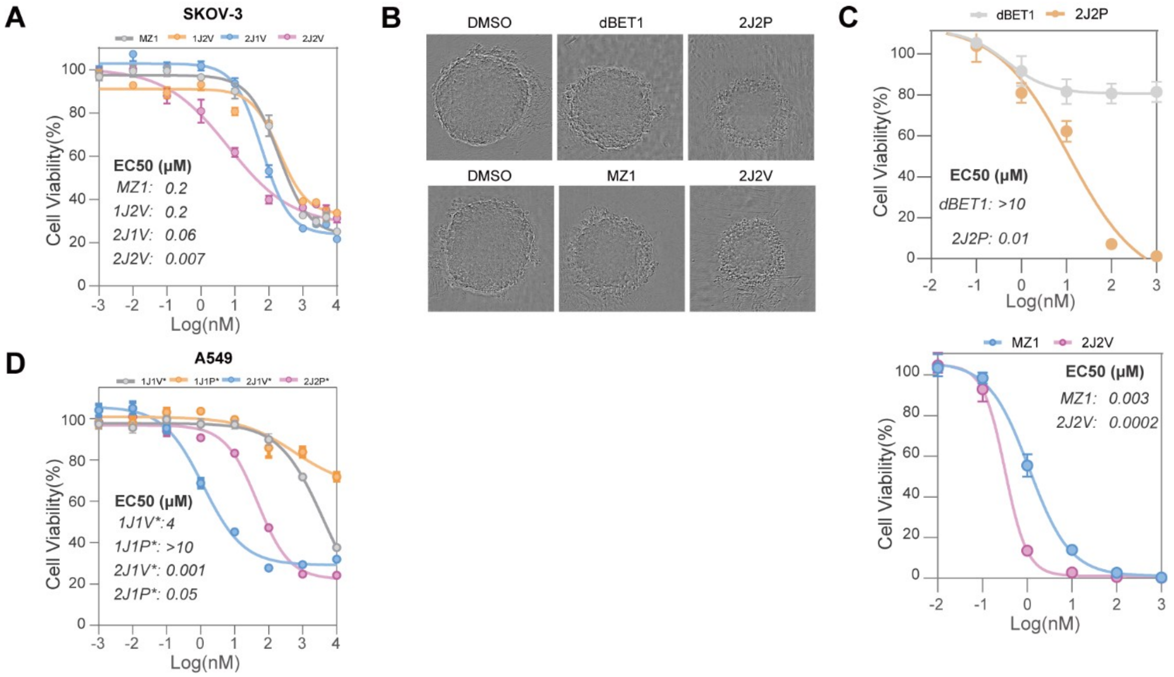

Figure S7. Dual-ligand PROTACs inhibit cancer cell growth in 2D and 3D *in vitro cell* cultures. (A) 2D-cultured SKOV-3 were treated with an escalating dose of PROTACs for 48h. Cell viability was assessed by CellTiter-Glo 2D assay. EC_{50} values were calculated from fitted curves. **(B)** HCT116 spheroids were treated with an escalating dose of PROTACs for 72h (equivalent volume of DMSO as control group). Representative transmitted microscopy images at the dose of 10nM. **(C)** Cell viability was assessed by Cell Titer-Glo 3D assay. Both experiments were performed as three independent replicates. **(D)** 2D-cultured A549 cells were treated with an escalating dose of PROTACs equipped with similar linker length as dual-ligand PROTACs. Cell viability was assessed by CellTiter-Glo 2D assay. EC50 values were calculated from fitted curves.

4.6 Mouse serum stability of 2J2V and MZ1

The mouse serum stability of compound **2J2V** and **MZ1** were studied in the company of Eurofins Panlabs, 6 Research Park Dr. St. Charles, MO 63304, U.S.A. Compounds were incubated in the mouse serum at 37°C for certain time, and at 0, 0.5, 1, 1.5, 2 hours post incubation, the mouse serum was analyzed by HPLC-MS/MS according to the reference: Di, L. et al. (2005), Int. J. Pharm., 297 (1-2): 110-119. At the end of incubation at each of the time points, acetonitrile was added to the incubation mixture followed by centrifugation. Samples were analyzed by HPLC-MS/MS and peak areas were recorded for each analyte. The area of precursor compound remaining after each of the time points relative to the amount remaining at time zero, expressed as percent, is calculated. Subsequently, the half-life (T1/2) is estimated from the slope of the initial linear range of the logarithmic curve of compound remaining (%) versus time, assuming first order kinetics. The results are shown in the table below.

Compound I.D.	Client Compound I.D.	Test Concentration	Incubation (minutes)	% Compound Remaining (% remaining) 1st	2nd	Mean	Half-Life (minutes) 1st	2nd	Mean
**Half-life (plasma, mouse, CD-1)**									
100067858-1	2J2V	1.0E-06 M	0	100.00	100.00	100.00	556.6	550.8	>120
100067858-1	2J2V	1.0E-06 M	30	101.79	99.37	100.58			
100067858-1	2J2V	1.0E-06 M	60	88.92	89.37	89.14			
100067858-1	2J2V	1.0E-06 M	90	89.07	{91.4}	89.07			
100067858-1	2J2V	1.0E-06 M	120	88.69	87.25	87.97			
100067858-2	MZ1	1.0E-06 M	0	100.00	100.00	100.00	2808.7	2774.1	>120
100067858-2	MZ1	1.0E-06 M	30	{90.9}	108.53	108.53			
100067858-2	MZ1	1.0E-06 M	60	98.07	107.65	102.86			
100067858-2	MZ1	1.0E-06 M	90	101.98	97.80	99.89			
100067858-2	MZ1	1.0E-06 M	120	95.29	{110.5}	95.29			

Table S1. Data of **2J2V** and **MZ1** level in mouse serum over time by HPLC-MS/MS.

4.7 Therapeutic anti-tumor evaluation

Animal experiments were carried out in accordance with the regulatory guidelines of the Ethics Committee of the Ghent University Hospital. 30 female Swiss Nude Immunodeficient mice (Crl:NU(Ico)-Foxn1nu) of 5 weeks-6 weeks old (Charles River) were subcutaneously injected with 1×10^6 A549 (1:1 serum free DMEM medium: Matrigel (Corning)). 10 days post inoculation ,mice were randomized into five groups (6 mice/group) with equal average tumor volume and treatment was started: (1) the control group: Vehicle(20% PEG400 and 5% Kolliphor in 75% PBS), (2) the **MZ1** group (0.1 mg/kg), (3) the **MZ1** group (0.5 mg/kg), (4) the **2J2V** group (0.1 mg/kg), (5) the **2J2V** group(0.5 mg/kg).Mice were treated on Day 0, Day 2, Day 4, Day 11, Day 18 by intraperitoneal injection. Tumor growth was monitored every 3 days by caliper, and the tumor volume was calculated as 1/2 × length × width^2. Body weight was monitored every 3 days by weighing scale. The mice were sacrificed on Day 21 after treatment.

4.8 Proteomics study

4.8.1 Sample preparation

A549 cells in DMEM medium were seeded at 1×10^6 cells on a 6-well plate 24 h before treatment. Cells were treated in triplicate by addition of 10 nM MZ1/2J2V or an equivalent volume of DMSO. After 4 h, the cells were digested by trypsin, centrifuged at 250g for 5min and washed three times with PBS. Cells were resuspended in lysis buffer (50 mM triethylammonium bicarbonate (TEAB), 5% SDS (pH 7.5)), then sonicated, and clarified with centrifugation of 13,000g for 8 minutes. Each sample was reduced with 20 mM DTT, then warmed the solution first at 95 °C for 10 minutes and then at 60 °C for 1 hour. The reduced proteins were alkylated with 40 mM iodoacetamide (IAA) in the dark for 30 minutes. Then samples were acidified with 1.2% aqueous phosphoric acid and mixed with 6× volumes of 90% methanol, 100 mM TEAB, pH 7.1 (S-Trap binding buffer). The samples were loaded onto the

filter of the S-Trap micro column (ProtiFi) and centrifuged at 4000×g for 30 seconds. The flow-through was discarded each time and the samples were washed 9 times with the S-Trap binding buffer. Proteins trapped in the S-Trap column were digested with Trypsin/Lys-C Mix (Promega, USA) at 47°C for 1 hour at a protein-to-enzyme ratio of 1:25 (w/w). The digested peptides were eluted with buffers in order: 50 mM TEAB pH 8.0, 0.2% aqueous formic acid, and 50% acetonitrile/0.2% aqueous formic acid. Then the samples were lyophilized using a SpeedVac (Thermo Fisher Scientific) with cold trap and desalted with C18 tips (Empore). Finally, the desalted peptides were again SpeedVac dried and dissolved in 0.1% formic acid for liquid chromatography-mass spectrometry analysis.

4.8.2 Liquid chromatography-tandem mass spectrometry analysis

The peptide samples were analyzed by a nanoflow HPLC system (Easy-nLC1200, Thermo Fisher Scientific) coupled to Orbitrap Fusion Lumos mass spectrometer (Thermo Fisher Scientific, Bremen, Germany) equipped with a high-field asymmetric waveform ion mobility spectrometry (FAIMS) device between a nano-electrospray ionization source and the mass spectrometer. Compensation voltages of -50 V and -70 V were used. Peptides were first loaded on a trapping column and then separated inline on a 15 cm C18 column (75 µm × 15 cm, ReproSil-Pur 3 µm 120 Å C18-AQ, Dr. Maisch HPLC GmbH, Ammerbuch-Entringen, Germany) with a 120-minute 2-step gradient consisting of solvents A (0.1% formic acid) and solvent B (acetonitrile/water (95:5(v/v)) with 0.1% formic acid) from 5–21% B in 62 minutes, 21-36% B in 48 minutes, 36-100% in 5 minutes and held at 100% B for 5 minutes. All samples were analyzed by data independent acquisition (DIA), in which each cycle consisted of a full scan of 395-1000 m/z at a resolution of 120,000, maximum injection time of 50 ms and automatic gain control (AGC) target of 7e5. DIA MS2 scans were employed for all DIA scans at a resolution of 30,000, AGC target 1e6, maximum injection time of 52 ms and 30 windows of variable window scheme with an overlap of 1 m/z. DIA spectra were acquired at both -50 V and -70 V FAIMS compensation voltages.

4.8.3 **Data availability.** The protemics data based on mass spectrometry in this study have been deposited to ProteomeXchange Consortium via the PRIDE (Perez-Riverol et al., 2022) partner repositoryat the European Bioinformatics Institute (http://www.ebi.ac.uk/pride/) and is now accessible with the dataset identifier PXD049407.

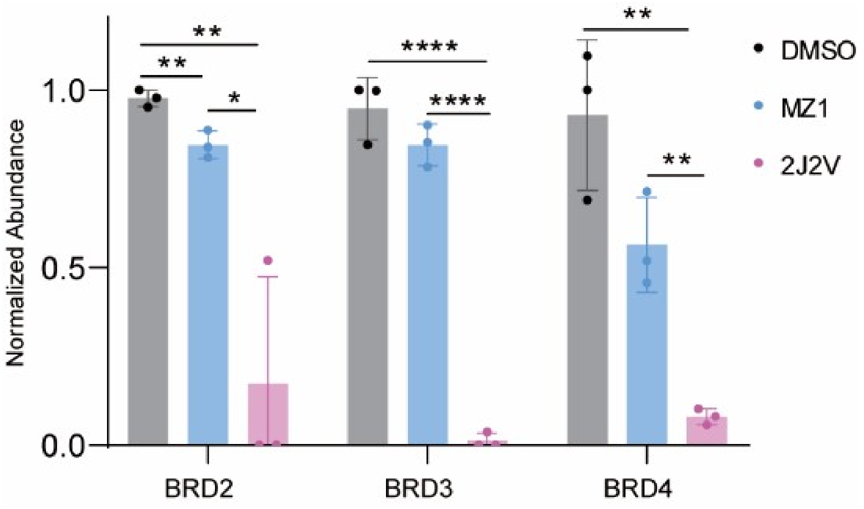

Figure S8 Quantitative analysis of BRD2, BRD3 and BRD4 protein levels from mass spectrometry proteomics on Figure 7. Data are presented as mean values with error bars representing the SD from triplicates. Student's t-test were performed for analysis. (*: $p<0.05$, **:$p<0.01$, ***:$p<0.001$, ****:$p<0.0001$)

4.9 Study of membrane permeability of single-ligand and dual-ligand PROTACs

Parallel artificial membrane permeability assay (PAMPA) is a non-cell based assay designed to predict passive, transcellular permeability of a test compound through a PVDF membrane filter pretreated with lipid. At a given pH a log Pe (log of effective permeability) can be determined by a UV spectrophotometer. The PAMPA study of **MZ1**, **dBET1**, **2J2V** and **2J2P** is carried out in the company of Eurofins Panlabs, 6 Research Park Dr. St. Charles, MO 63304, U.S.A. The results are attached as an excel file.

Sample preparation The test compound is prepared from a 10 mM DMSO stock solution and diluted to a single final concentration of 10 uM in donor buffer. The donor solution is placed in contact with the acceptor buffer at a single pH = 7.4 with the membrane in between and the sandwich is incubated for four hours at ambient temperature.

Reference compounds Ketoprofen, propranolol and furosemide. The reference compounds yield log Pe (10-6 cm/s) values that cover the typical range of the assay from high (Propranolol), mid (Ketoprofen) to low (Furosemide) permeability.

Analytical method UV/Vis spectrophotometer

The UV absorption is measured from 250 to 500 nm for both donor and acceptor plate.

Adjustments to the method may be made as necessary to improve detection or UV spectrum.

Data analysis The amount of compound UV absorbance is determined in the donor plate prior to starting the experiment for each compound. The UV absorbance was measured from the

donor and acceptor plate after a four hour incubation time. The log Pe was calculated using the equation below.

$$\log P_e = \log \{C \bullet -1n[1 - \frac{[drug]_{acceptor}}{[drug]_{equilibrium}}]\} \text{ where } C = (\frac{V_D \bullet V_A}{(V_D + V_A) \text{ Area} \bullet \text{time}})$$

4.10 Statistical Analysis.

Data analyses were performed using GraphPad Prism 9 software. The results are presented as the mean ± standard deviation (SD) or standard error of mean (SEM). All statistical analyses were evaluated with one-way analysis of variance (ANOVAs), or unpaired Student’s t-test. The difference was considered statistically significant when $P < 0.05$.

5. NMR spectra

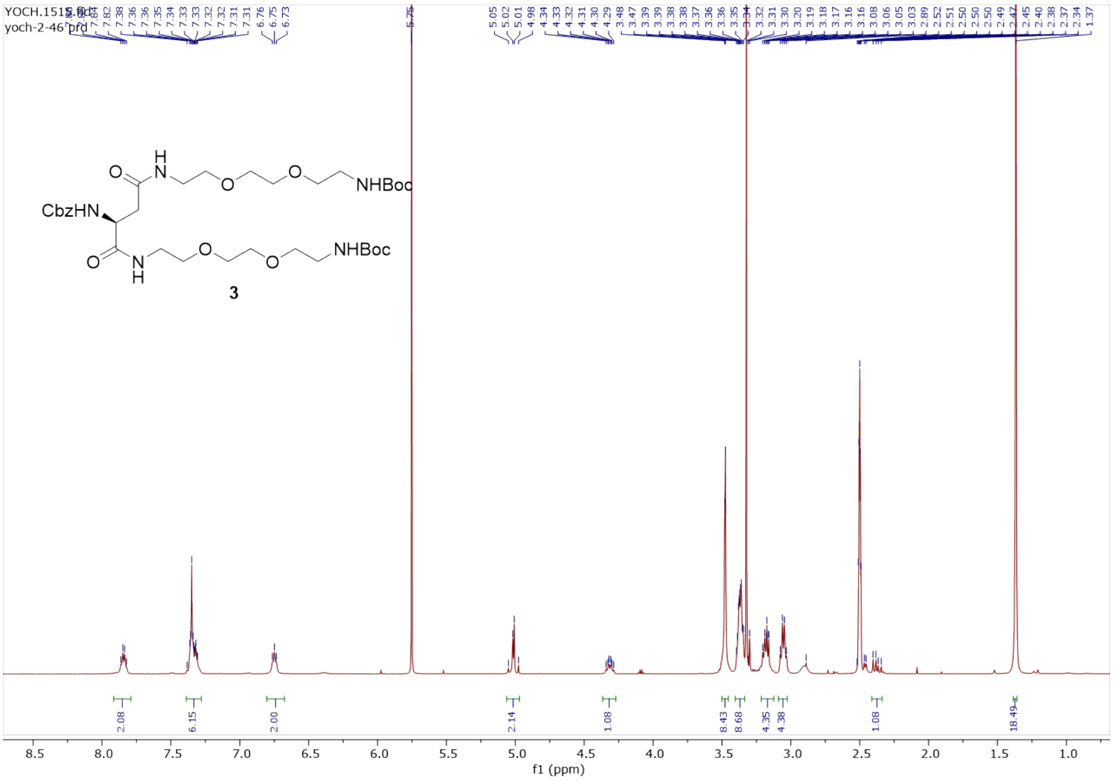

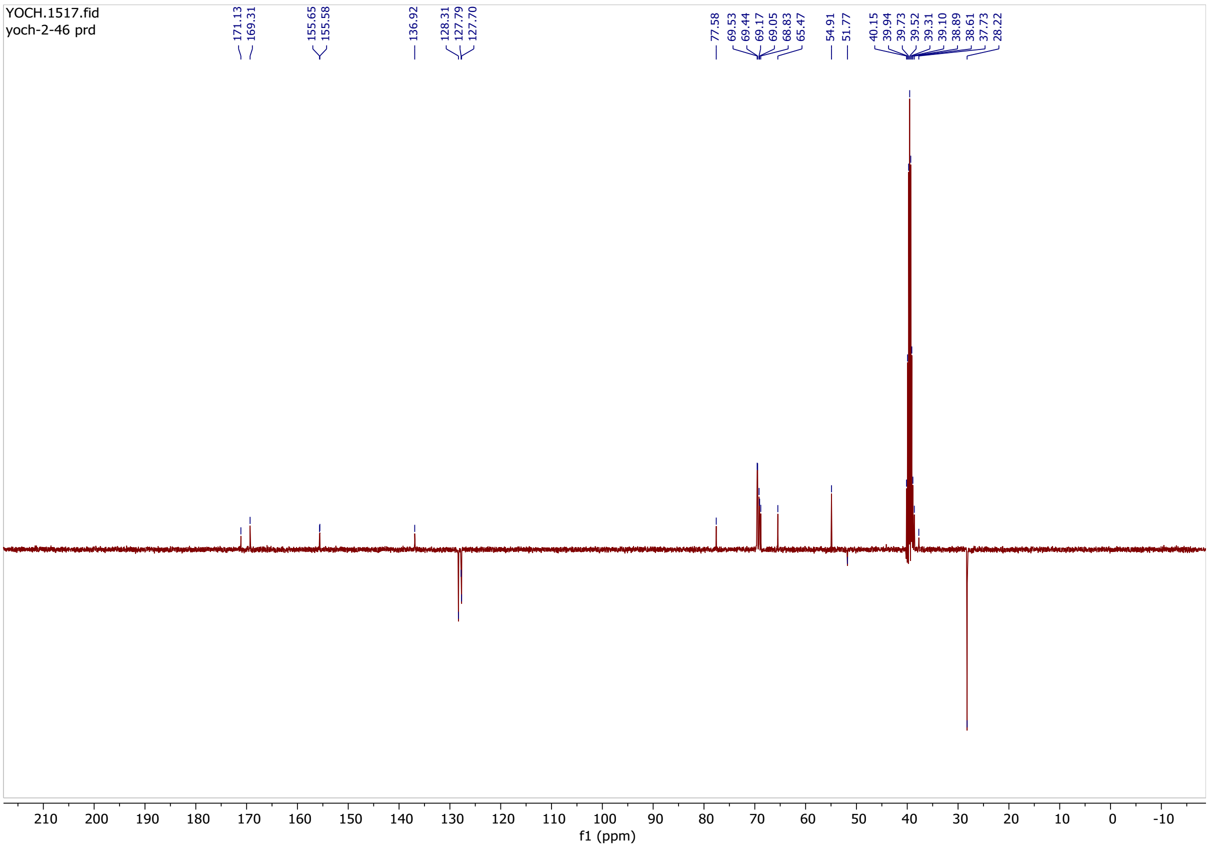

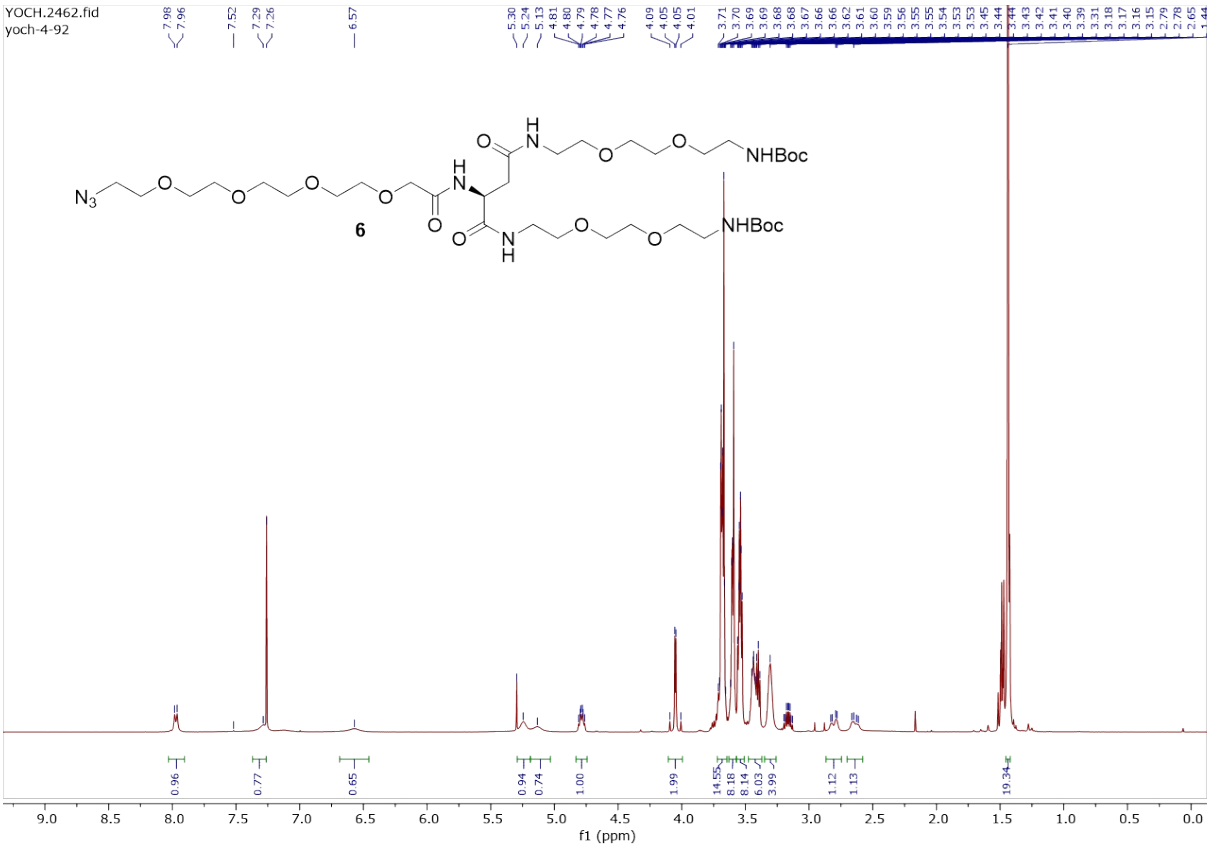
YOCH.2462.fid
yoch-4-92
6
f1 (ppm)

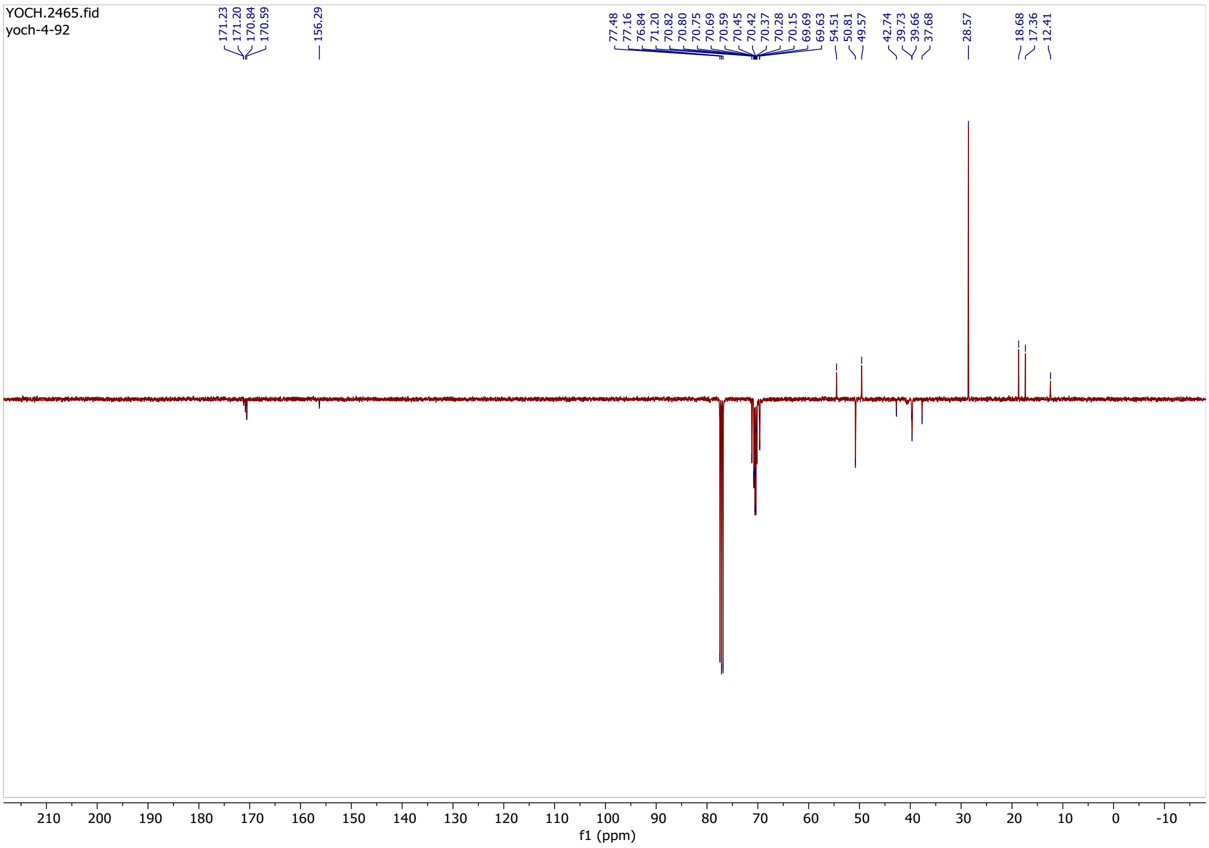
YOCH.2465.fid
yoch-4-92
f1 (ppm)

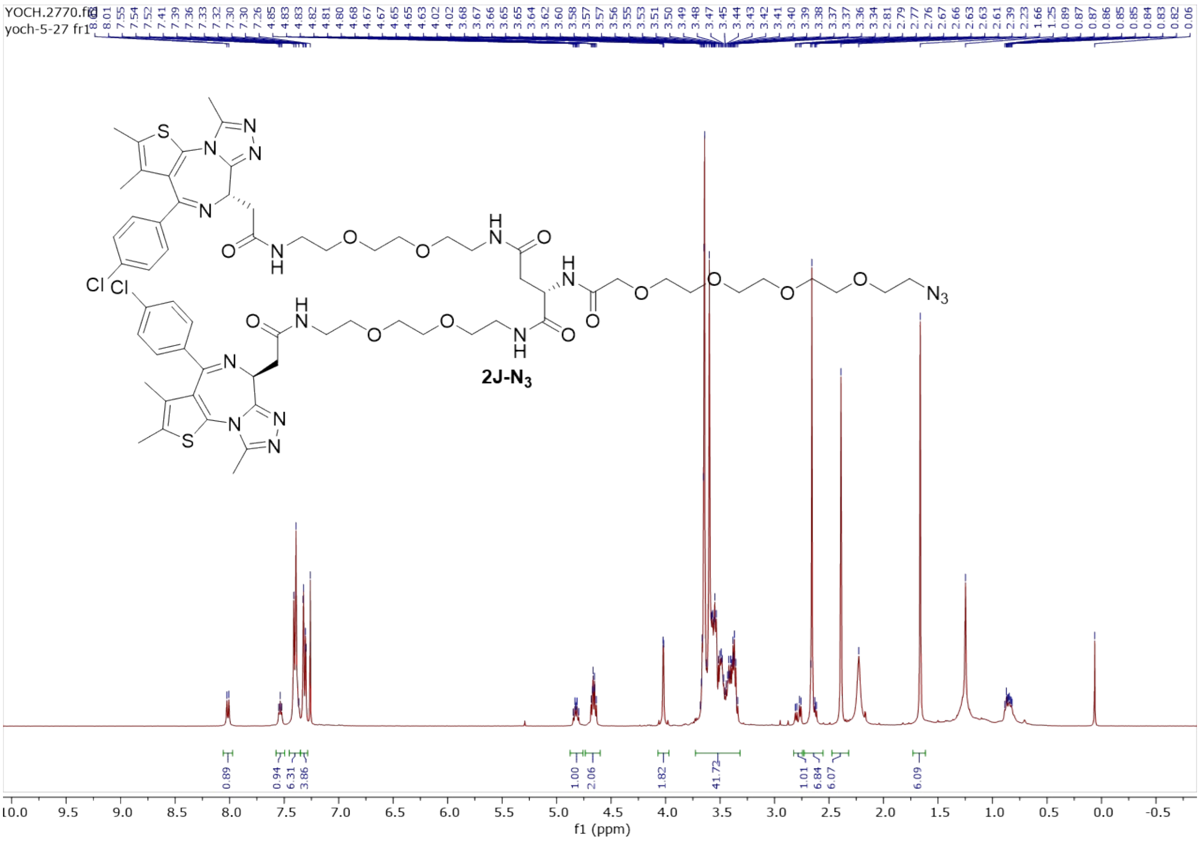

YOCH.2770.fid
yoch-5-27 fr1
[illegible] 8.01 7.55 7.54 7.52 7.41 7.39 7.36 7.33 7.32 7.30 7.30 7.26 4.85 4.83 4.83 4.82 4.81 4.80 4.68 4.67 4.67 4.65 4.65 4.63 4.02 4.02 3.68 3.67 3.66 3.65 3.65 3.65 3.64 3.62 3.60 3.58 3.57 3.57 3.56 3.55 3.53 3.51 3.50 3.49 3.48 3.47 3.45 3.45 3.43 3.42 3.41 3.40 3.39 3.38 3.37 3.37 3.36 3.34 2.81 2.79 2.77 2.76 2.67 2.66 2.63 2.63 2.61 2.39 2.23 1.66 1.25 0.89 0.87 0.87 0.86 0.85 0.85 0.84 0.83 0.82 0.06
2J-N3
0.89
0.94
6.31
3.86
1.00
2.06
1.82
41.72
1.01
6.84
6.07
6.09
f1 (ppm)

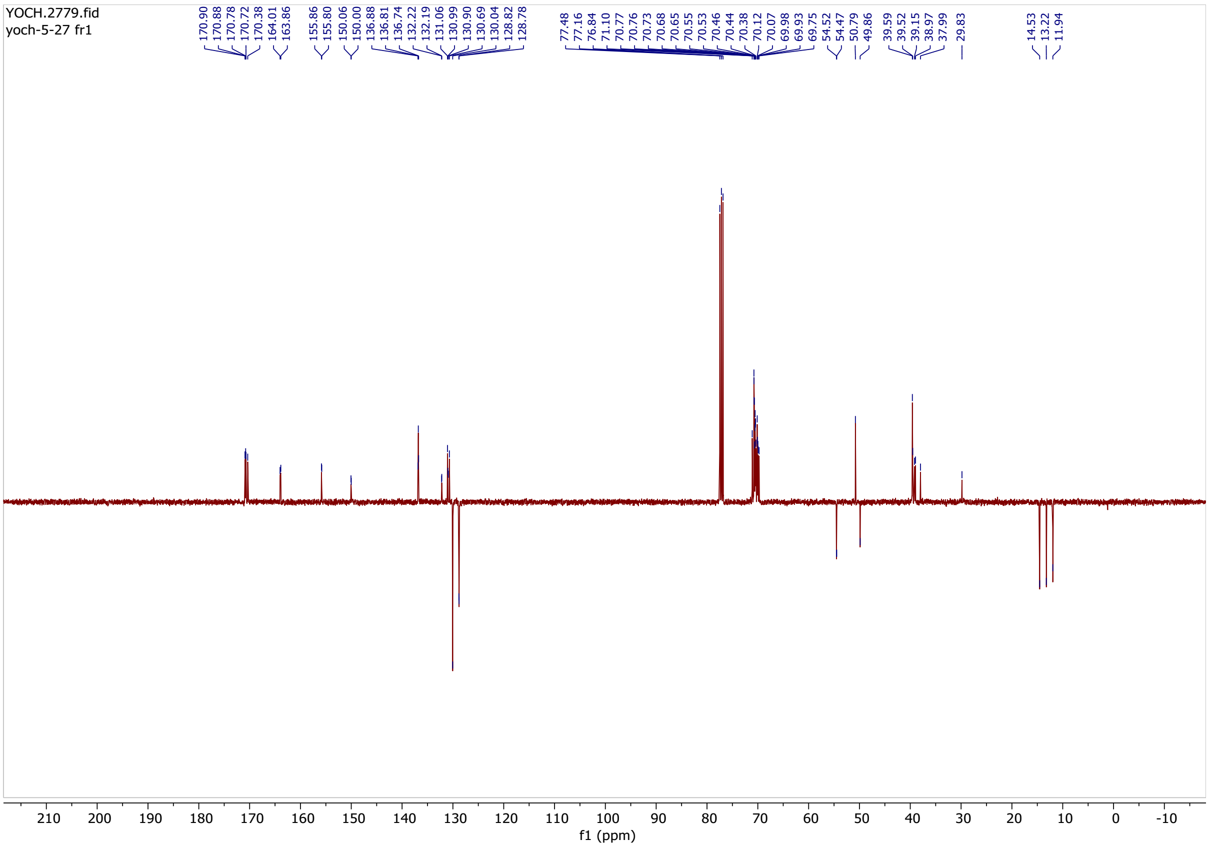

YOCH.2779.fid
yoch-5-27 fr1
170.90 170.88 170.78 170.72 170.38 164.01 163.86 155.86 155.80 150.06 150.00 136.88 136.81 136.74 132.22 132.19 131.06 130.99 130.90 130.69 130.04 128.82 128.78 77.48 77.16 76.84 71.10 70.77 70.76 70.73 70.68 70.65 70.55 70.53 70.46 70.44 70.38 70.12 70.07 69.98 69.93 69.75 54.52 54.47 50.79 49.86 39.59 39.52 39.15 38.97 37.99 29.83 14.53 13.22 11.94
f1 (ppm)

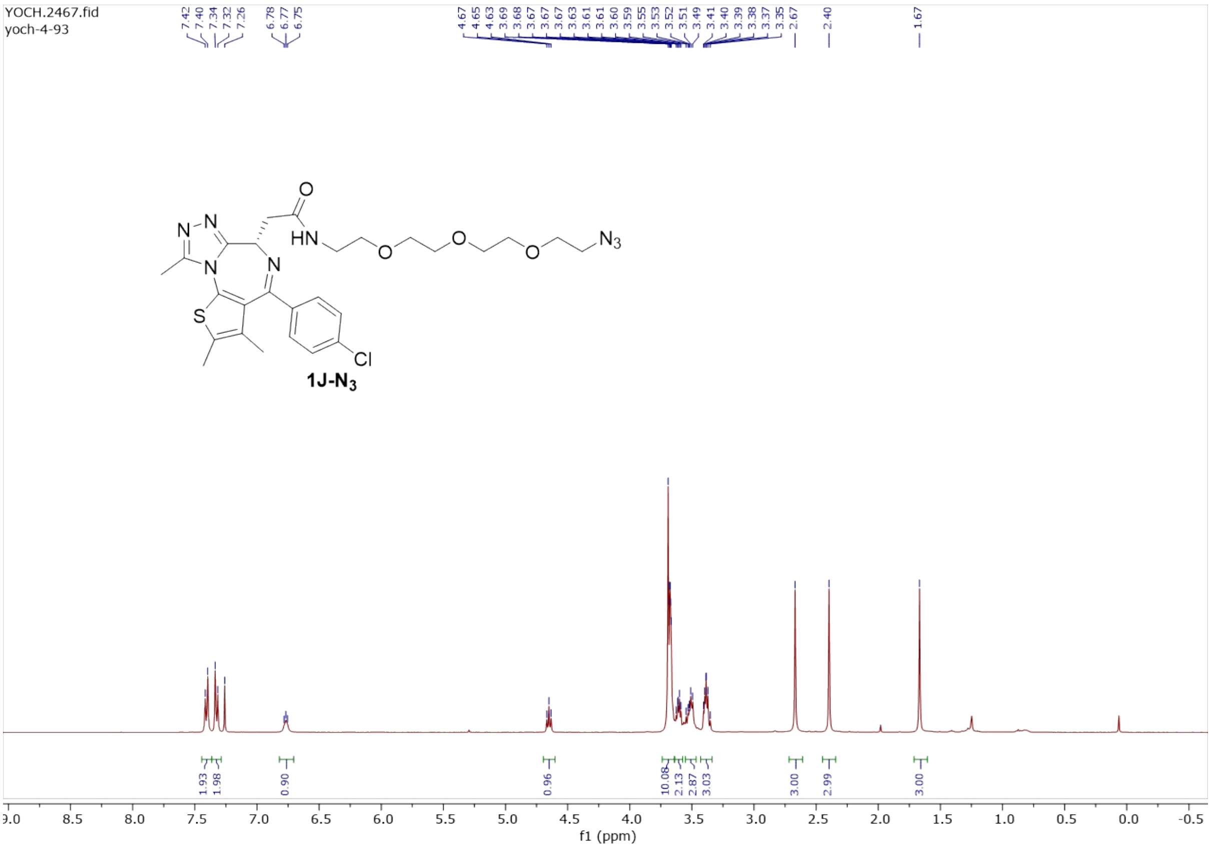
YOCH.2467.fid
yoch-4-93
7.42 7.40 7.34 7.32 7.26 6.78 6.77 6.75
4.67 4.65 4.63 3.69 3.68 3.67 3.67 3.67 3.63 3.61 3.61 3.60 3.59 3.55 3.53 3.52 3.51 3.49 3.41 3.40 3.39 3.38 3.37 3.35 2.67 2.40 1.67
1J-N3
1.93 1.98 0.90 0.96 10.08 2.13 2.87 3.03 3.00 2.99 3.00
f1 (ppm)

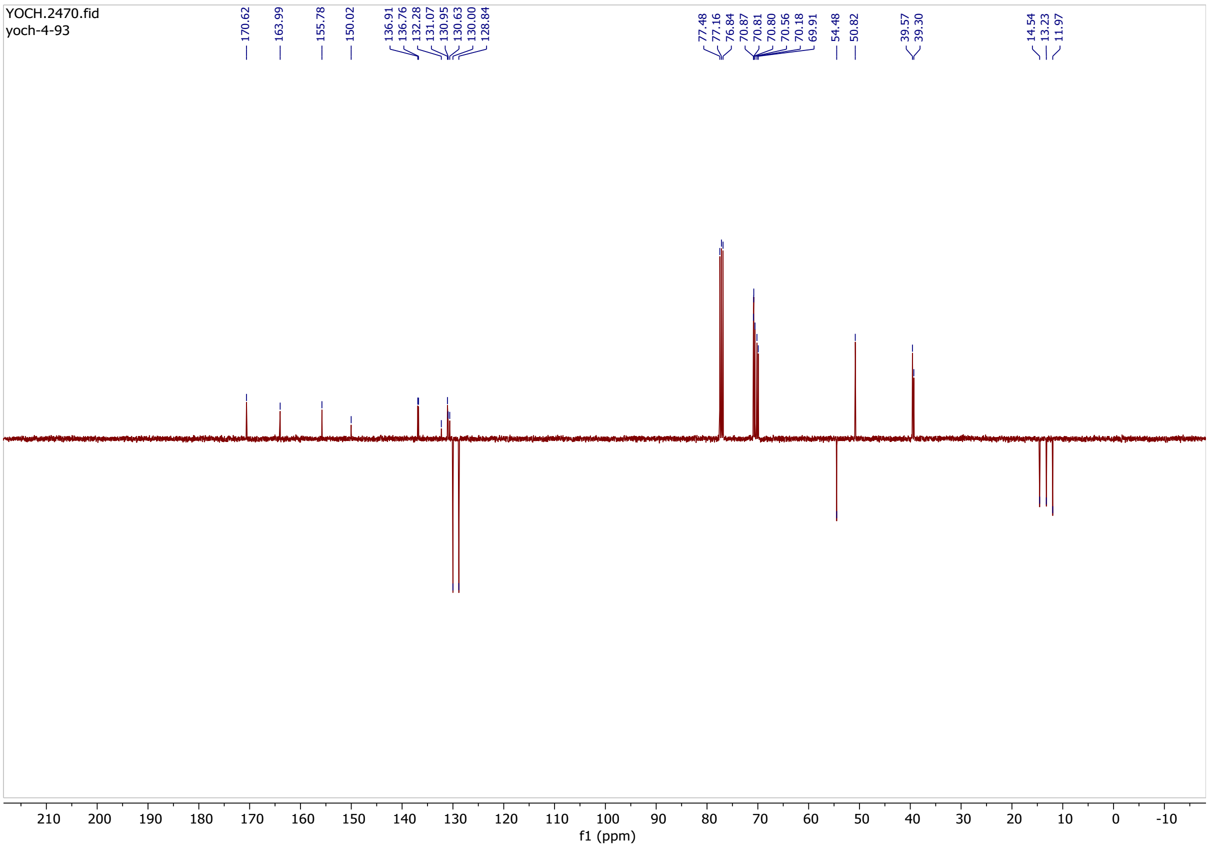
YOCH.2470.fid
yoch-4-93
170.62 163.99 155.78 150.02 136.91 136.76 132.28 131.07 130.95 130.63 130.00 128.84
77.48 77.16 76.84 70.87 70.81 70.80 70.56 70.18 69.91 54.48 50.82 39.57 39.30 14.54 13.23 11.97
f1 (ppm)

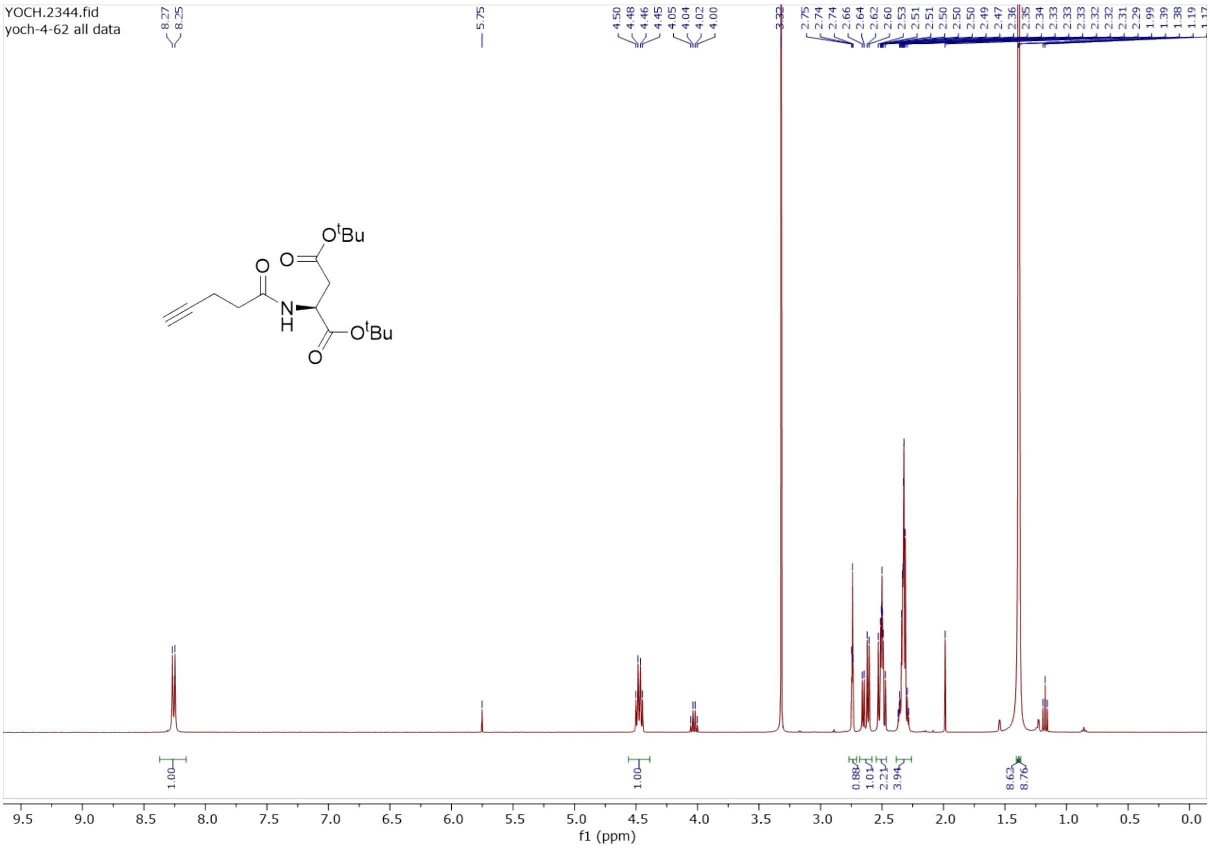
YOCH.2344.fid
yoch-4-62 all data
8.27
8.25
5.75
4.50
4.48
4.46
4.45
4.05
4.04
4.02
4.00
3.32
2.75
2.74
2.74
2.66
2.64
2.62
2.60
2.53
2.51
2.51
2.50
2.50
2.50
2.49
2.47
2.36
2.35
2.34
2.33
2.33
2.33
2.32
2.32
2.31
2.29
1.99
1.39
1.38
1.19
1.17
O
O
N
H
OtBu
OtBu
O
1.00
1.00
0.88
1.01
2.21
3.94
8.62
8.76
9.5 9.0 8.5 8.0 7.5 7.0 6.5 6.0 5.5 5.0 4.5 4.0 3.5 3.0 2.5 2.0 1.5 1.0 0.5 0.0
f1 (ppm)

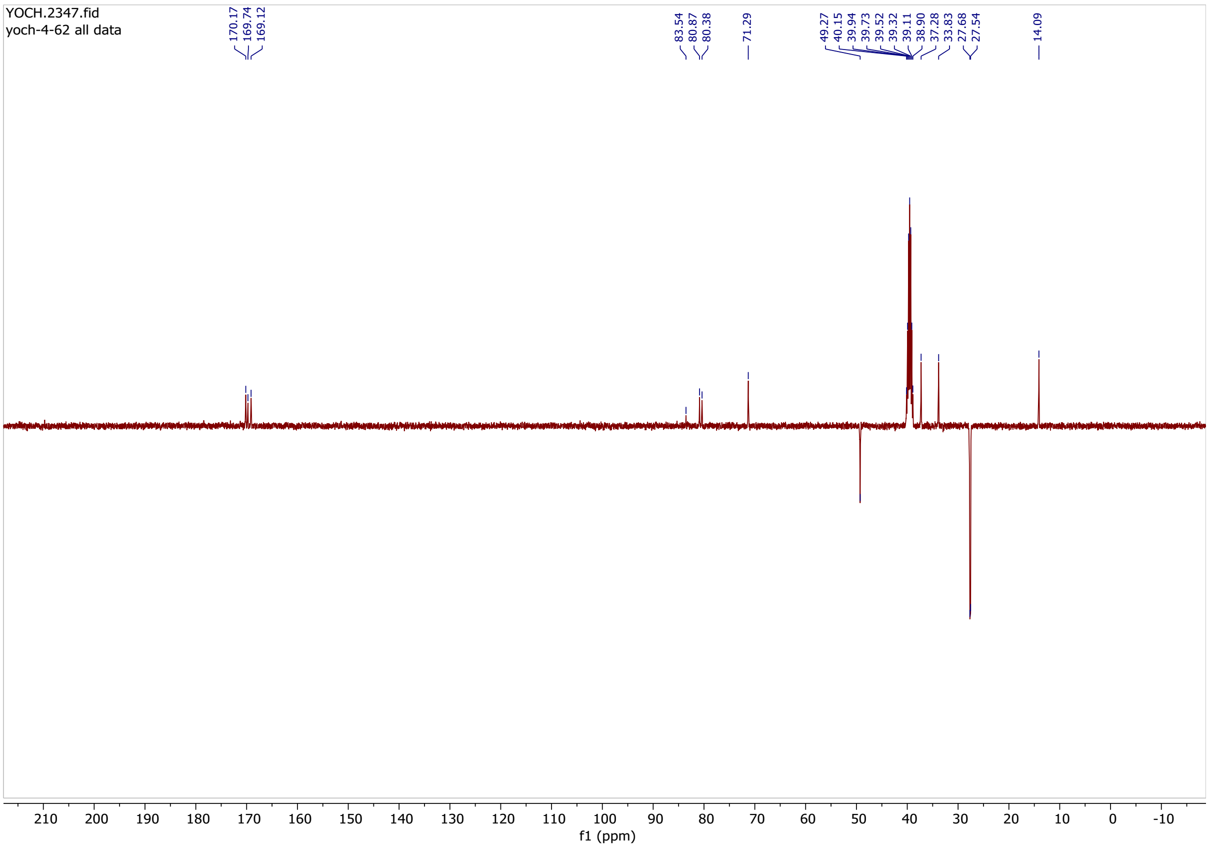
YOCH.2347.fid
yoch-4-62 all data
170.17
169.74
169.12
83.54
80.87
80.38
71.29
49.27
40.15
39.94
39.73
39.52
39.32
39.11
38.90
37.28
33.83
27.68
27.54
14.09
210 200 190 180 170 160 150 140 130 120 110 100 90 80 70 60 50 40 30 20 10 0 -10
f1 (ppm)

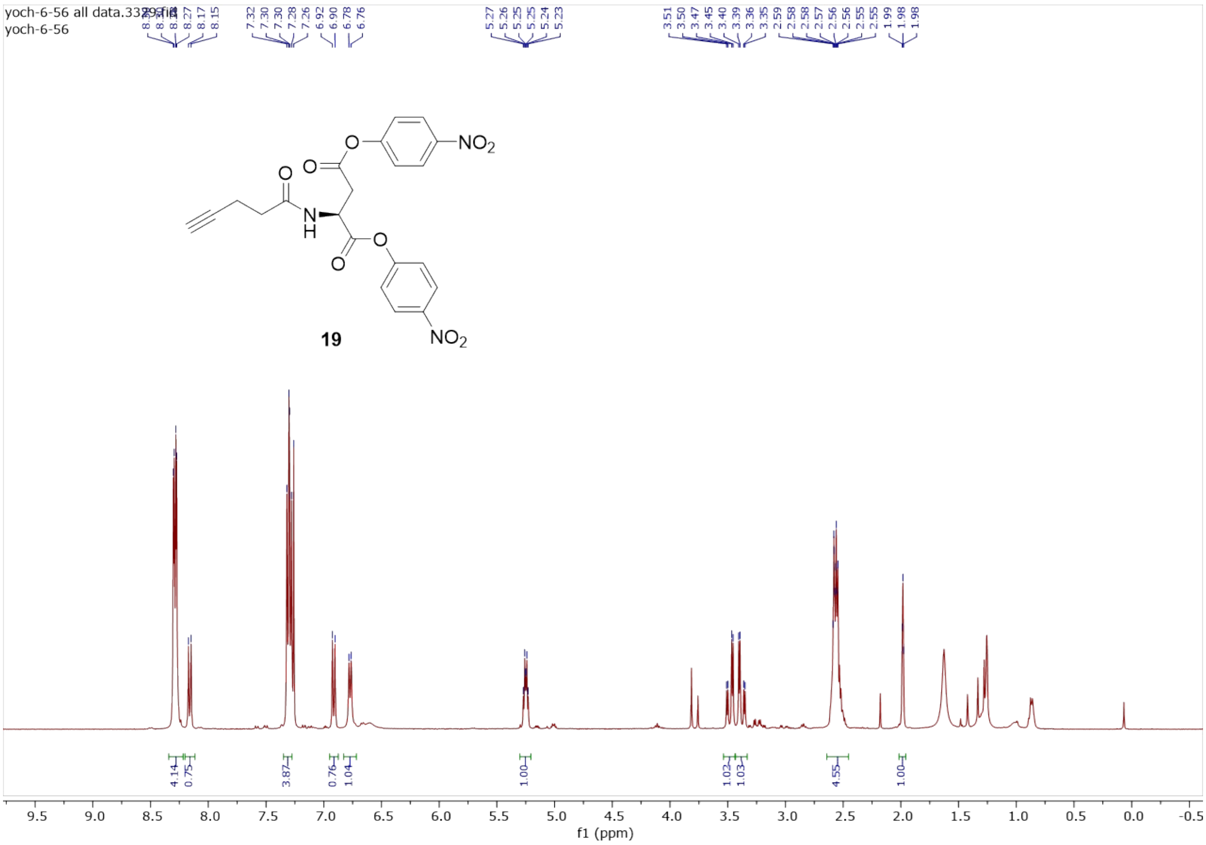
yoch-6-56
NO2
NO2
19
f1 (ppm)

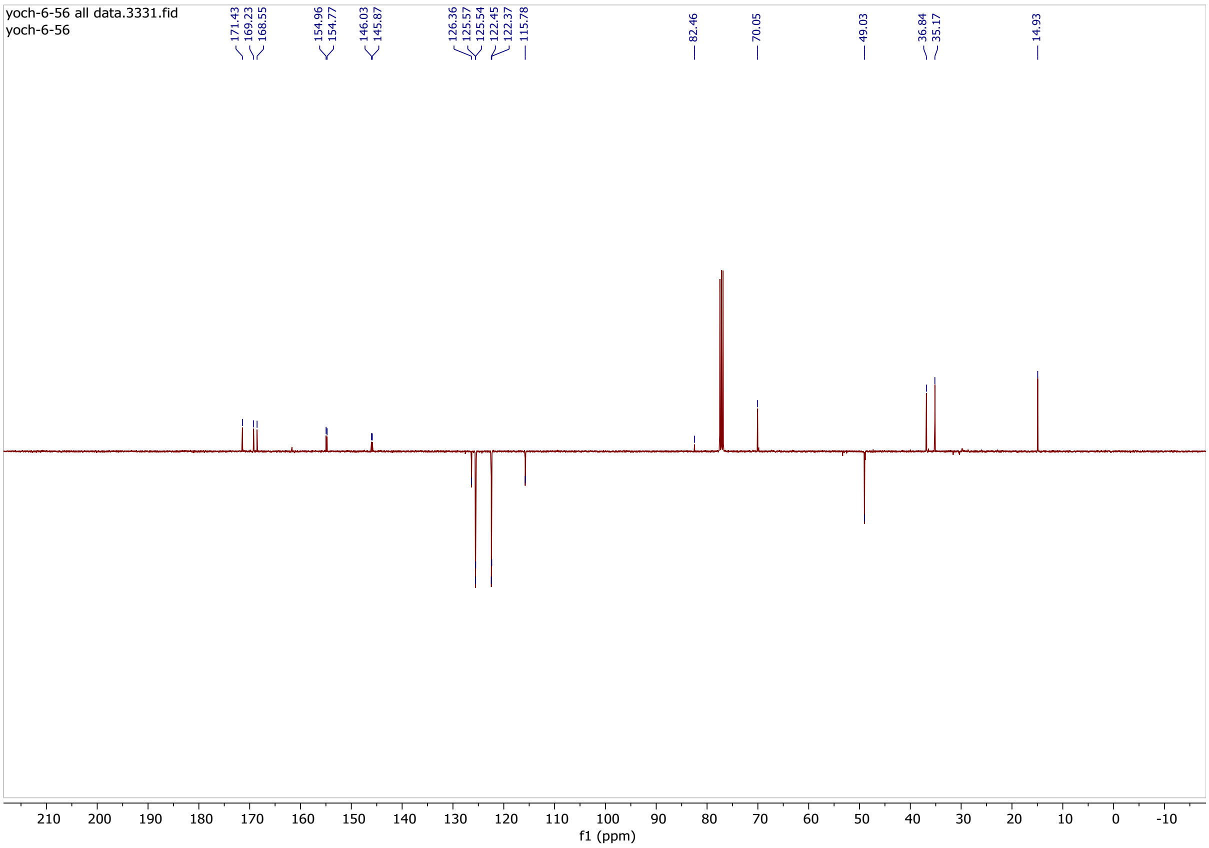
yoch-6-56 all data.3331.fid
yoch-6-56
171.43
169.23
168.55
154.96
154.77
146.03
145.87
126.36
125.57
125.54
122.45
122.37
115.78
82.46
70.05
49.03
36.84
35.17
14.93
f1 (ppm)

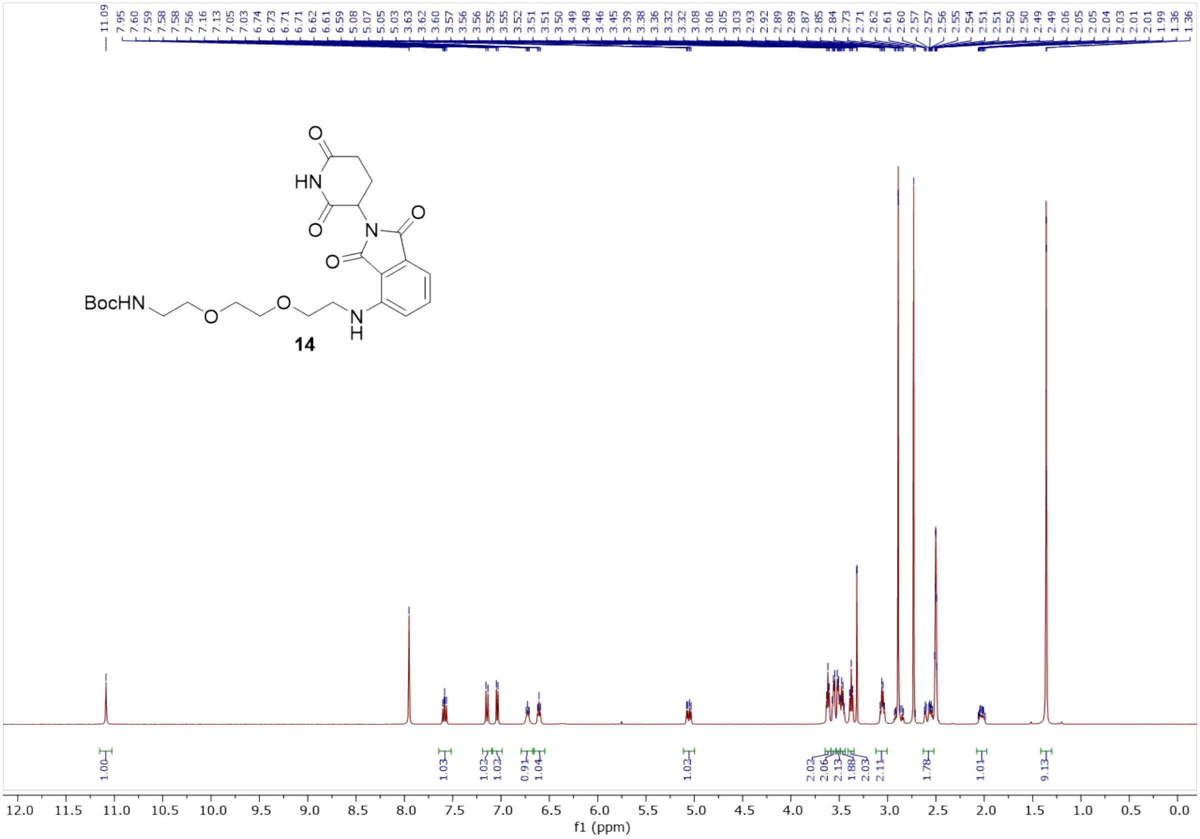
11.09
7.95
7.60
7.59
7.58
7.58
7.56
7.16
7.13
7.05
7.03
6.74
6.73
6.71
6.71
6.62
6.61
6.59
5.08
5.07
5.05
5.03
3.63
3.62
3.60
3.57
3.56
3.56
3.55
3.55
3.52
3.51
3.51
3.50
3.49
3.48
3.46
3.45
3.39
3.38
3.36
3.32
3.32
3.08
3.06
3.05
3.03
2.93
2.92
2.89
2.89
2.87
2.85
2.84
2.73
2.71
2.62
2.61
2.60
2.57
2.57
2.56
2.55
2.54
2.54
2.51
2.51
2.50
2.50
2.49
2.49
2.06
2.05
2.05
2.04
2.03
2.03
2.01
2.01
1.99
1.36
1.36
O
HN
O
N
O
O
BocHN
O
O
N
H
14
1.00
1.03
1.02
1.03
0.91
1.04
1.02
2.02
2.06
2.13
1.88
2.03
2.11
1.78
1.01
9.13
12.0 11.5 11.0 10.5 10.0 9.5 9.0 8.5 8.0 7.5 7.0 6.5 6.0 5.5 5.0 4.5 4.0 3.5 3.0 2.5 2.0 1.5 1.0 0.5 0.0
f1 (ppm)

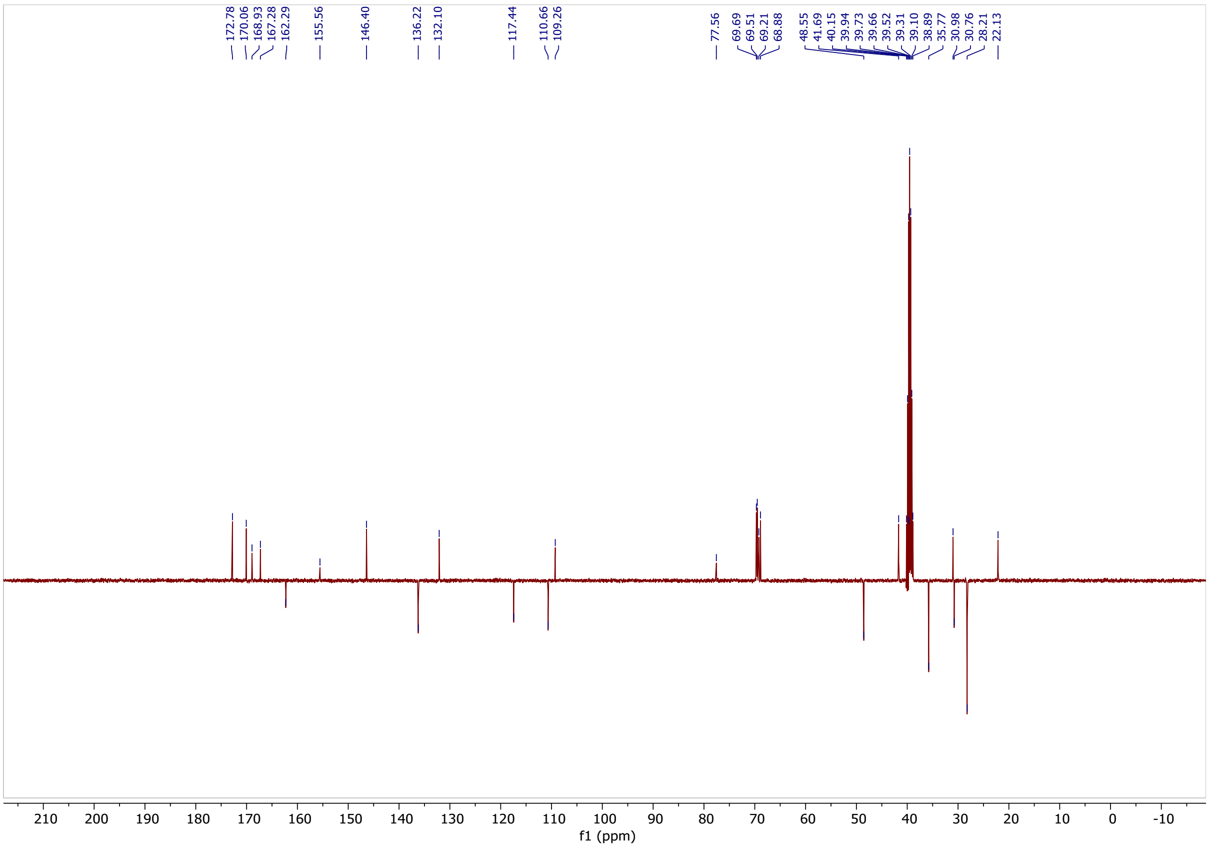
172.78
170.06
168.93
167.28
162.29
155.56
146.40
136.22
132.10
117.44
110.66
109.26
77.56
69.69
69.51
69.21
68.88
48.55
41.69
40.15
39.94
39.73
39.66
39.52
39.31
39.10
38.89
35.77
30.98
30.76
28.21
22.13
210 200 190 180 170 160 150 140 130 120 110 100 90 80 70 60 50 40 30 20 10 0 -10
f1 (ppm)

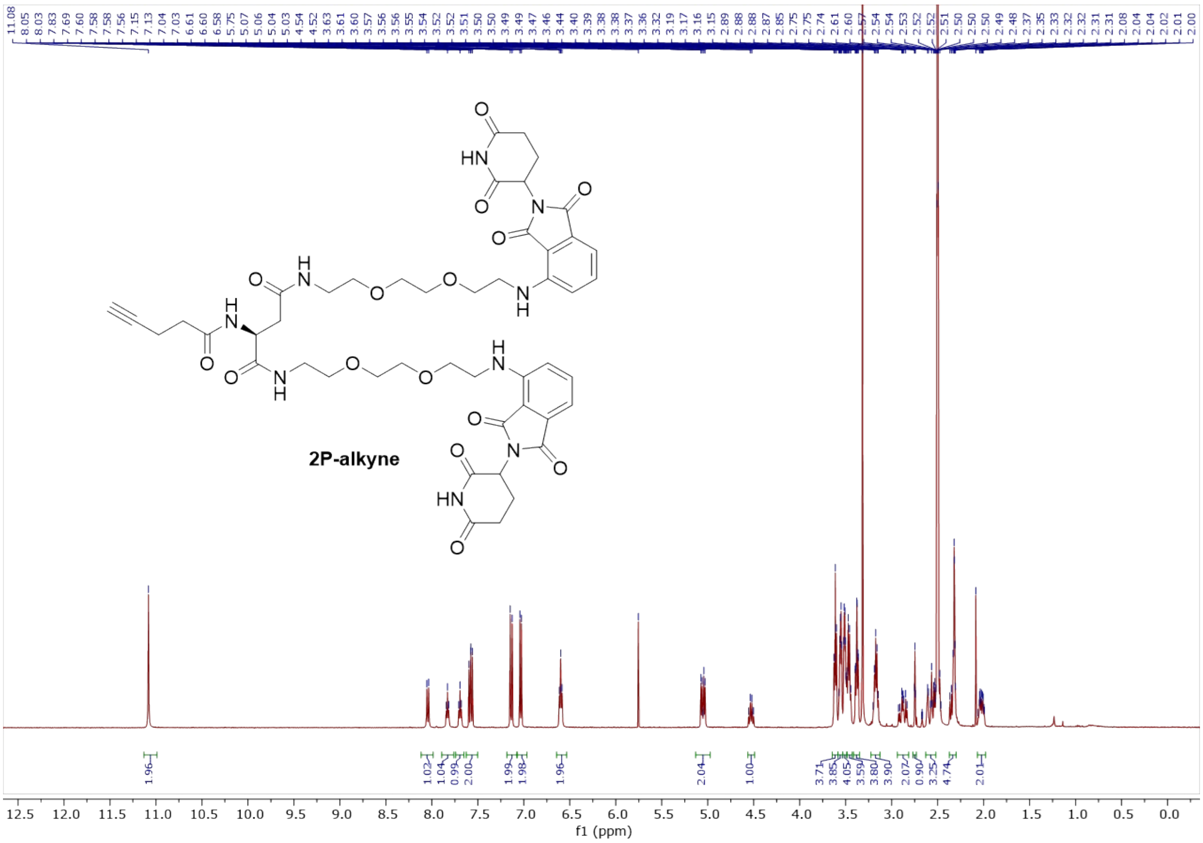

11.08 8.05 8.03 7.83 7.69 7.60 7.58 7.58 7.56 7.15 7.13 7.04 7.03 6.61 6.60 6.58 5.75 5.07 5.06 5.04 5.03 4.54 4.52 3.63 3.61 3.60 3.57 3.56 3.56 3.55 3.54 3.52 3.52 3.51 3.50 3.50 3.49 3.49 3.47 3.46 3.44 3.40 3.39 3.38 3.38 3.37 3.36 3.32 3.19 3.17 3.16 3.15 2.89 2.88 2.88 2.87 2.85 2.75 2.75 2.74 2.61 2.60 2.57 2.54 2.54 2.53 2.52 2.52 2.51 2.50 2.50 2.49 2.48 2.37 2.35 2.33 2.32 2.32 2.31 2.31 2.08 2.04 2.04 2.02 2.01 2.00
HN
N
O
H
2P-alkyne
1.96 1.02 1.04 0.99 2.00 1.99 1.98 1.96 2.04 1.00 3.71 3.85 4.05 3.59 3.80 3.90 2.07 0.90 3.25 4.74 2.01
12.5 12.0 11.5 11.0 10.5 10.0 9.5 9.0 8.5 8.0 7.5 7.0 6.5 6.0 5.5 5.0 4.5 4.0 3.5 3.0 2.5 2.0 1.5 1.0 0.5 0.0
f1 (ppm)

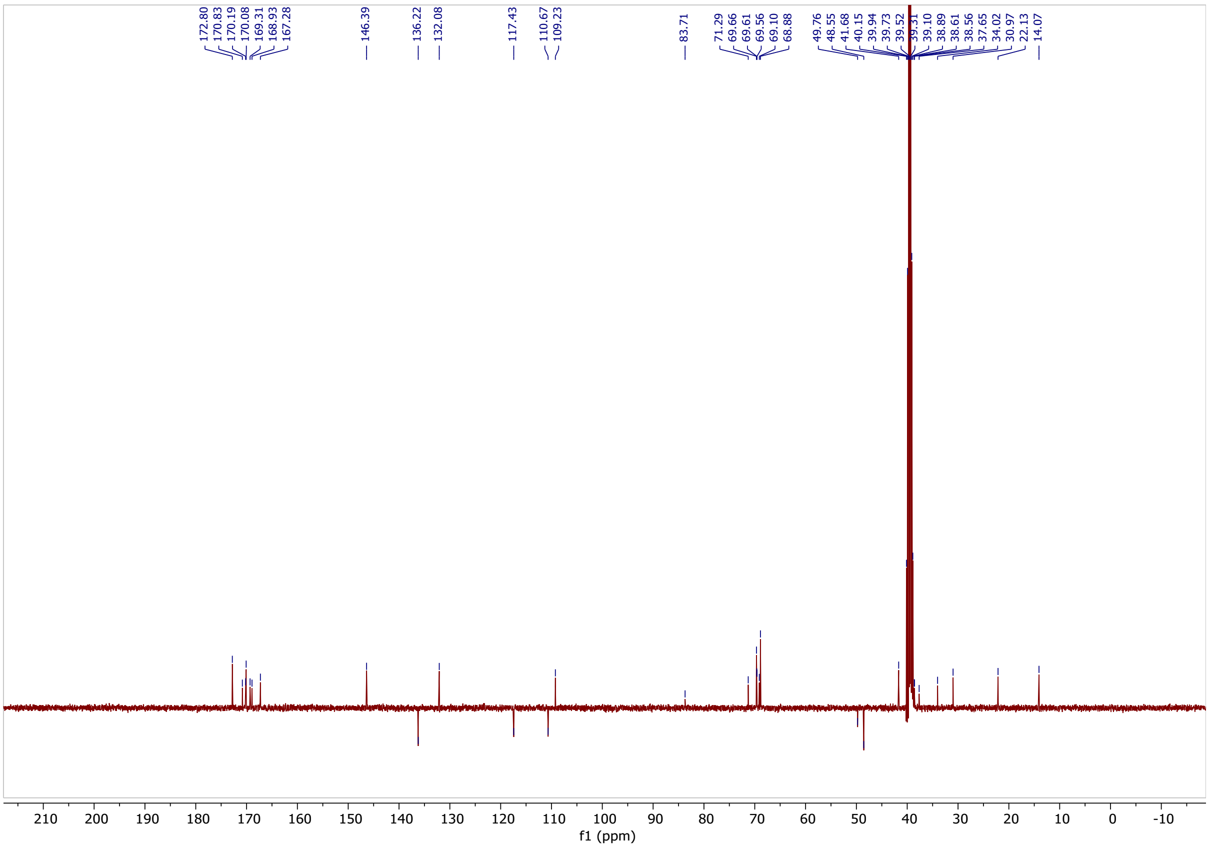

172.80 170.83 170.19 170.08 169.31 168.93 167.28 146.39 136.22 132.08 117.43 110.67 109.23 83.71 71.29 69.66 69.61 69.56 69.10 68.88 49.76 48.55 41.68 40.15 39.94 39.73 39.52 39.31 39.10 38.89 38.61 38.56 37.65 34.02 30.97 22.13 14.07
210 200 190 180 170 160 150 140 130 120 110 100 90 80 70 60 50 40 30 20 10 0 -10
f1 (ppm)

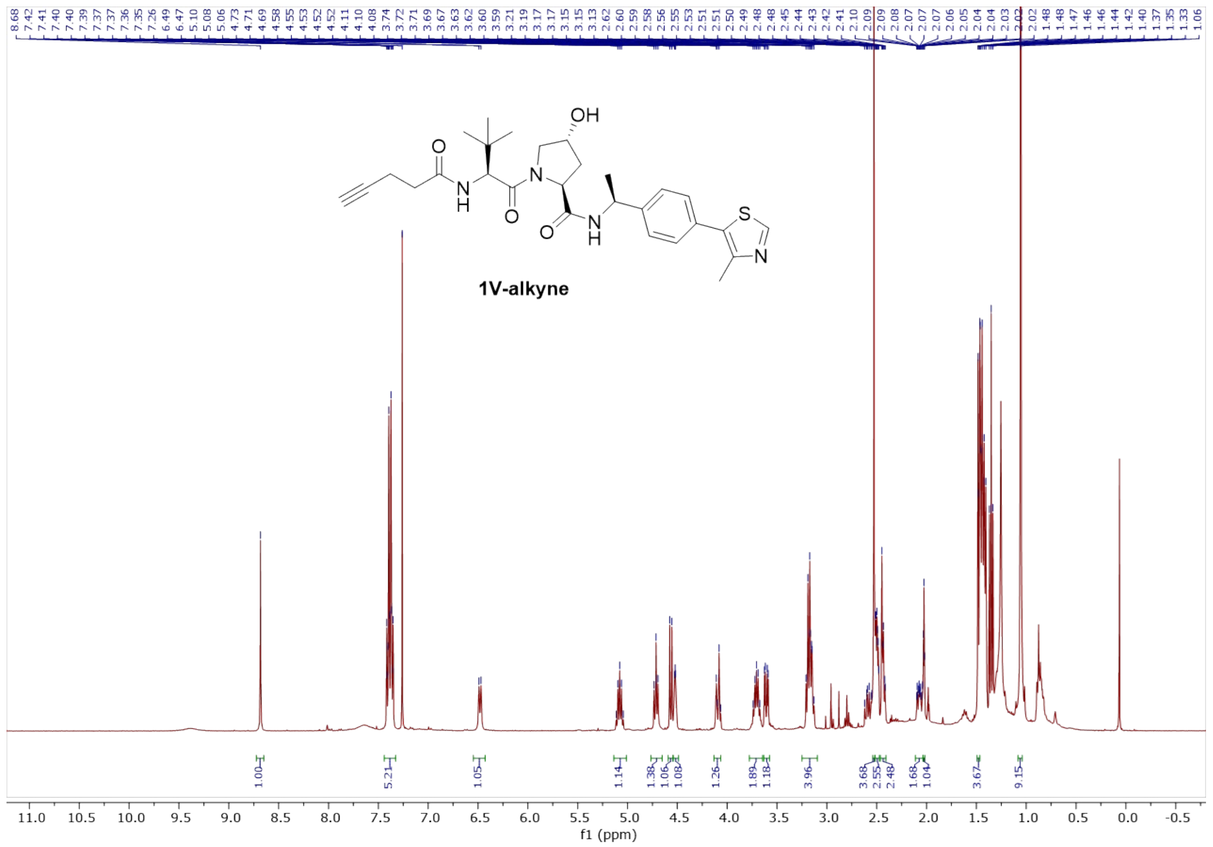
8.68 7.42 7.41 7.40 7.40 7.39 7.37 7.37 7.36 7.35 7.26 6.49 6.47 5.10 5.08 5.06 4.73 4.71 4.69 4.58 4.55 4.53 4.52 4.52 4.11 4.08 3.74 3.72 3.71 3.69 3.67 3.63 3.62 3.60 3.59 3.21 3.19 3.17 3.17 3.15 3.15 3.13 2.62 2.60 2.59 2.58 2.56 2.55 2.53 2.51 2.51 2.50 2.49 2.48 2.48 2.45 2.44 2.43 2.42 2.41 2.10 2.09 2.09 2.08 2.07 2.07 2.07 2.06 2.05 2.04 2.04 2.03 2.02 2.02 1.48 1.48 1.47 1.46 1.46 1.44 1.42 1.40 1.37 1.35 1.33 1.06
OH
O
N
H
O
N
O
N
H
S
N
1V-alkyne
1.00 5.21 1.05 1.14 1.38 1.06 1.08 1.26 1.89 1.18 3.96 3.68 2.55 2.48 1.68 1.04 3.67 9.15
11.0 10.5 10.0 9.5 9.0 8.5 8.0 7.5 7.0 6.5 6.0 5.5 5.0 4.5 4.0 3.5 3.0 2.5 2.0 1.5 1.0 0.5 0.0 -0.5
f1 (ppm)

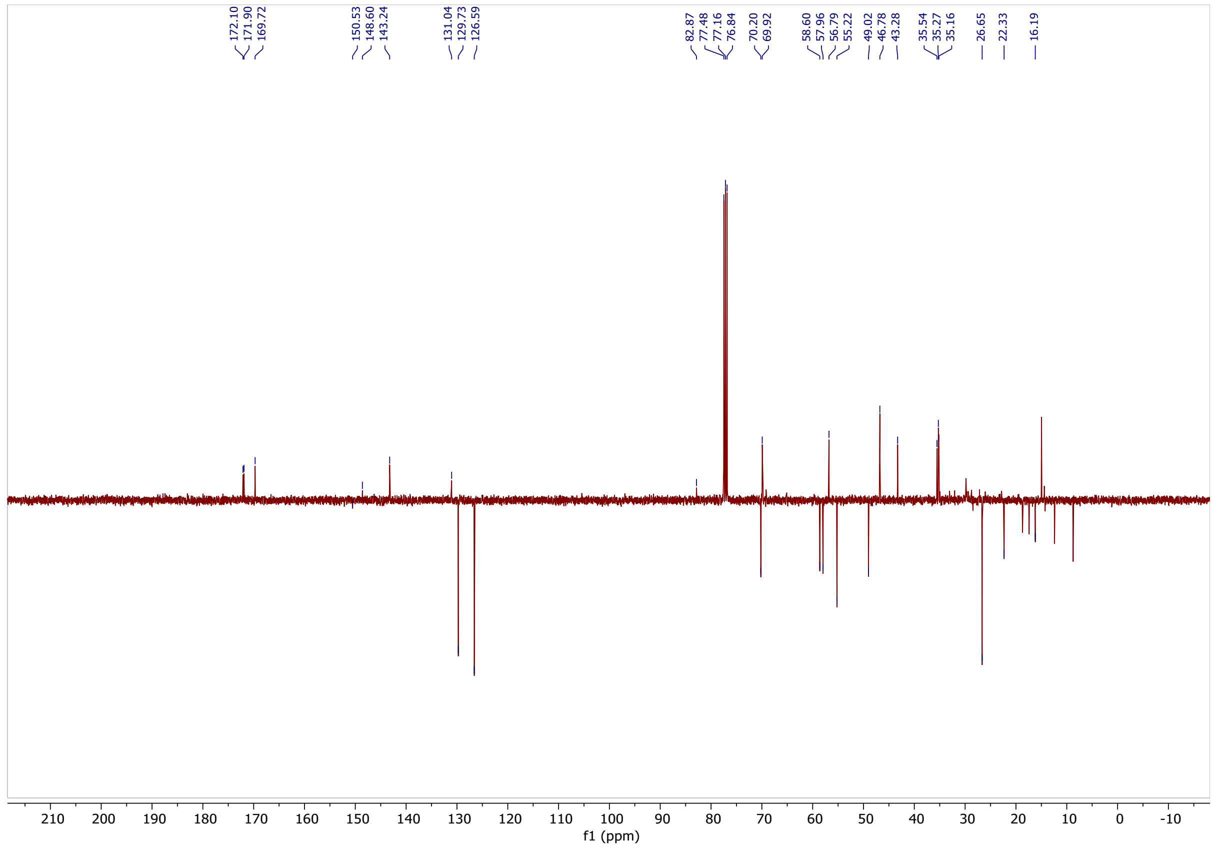
172.10 171.90 169.72 150.53 148.60 143.24 131.04 129.73 126.59 82.87 77.48 77.16 76.84 70.20 69.92 58.60 57.96 56.79 55.22 49.02 46.78 43.28 35.54 35.27 35.16 26.65 22.33 16.19
210 200 190 180 170 160 150 140 130 120 110 100 90 80 70 60 50 40 30 20 10 0 -10
f1 (ppm)

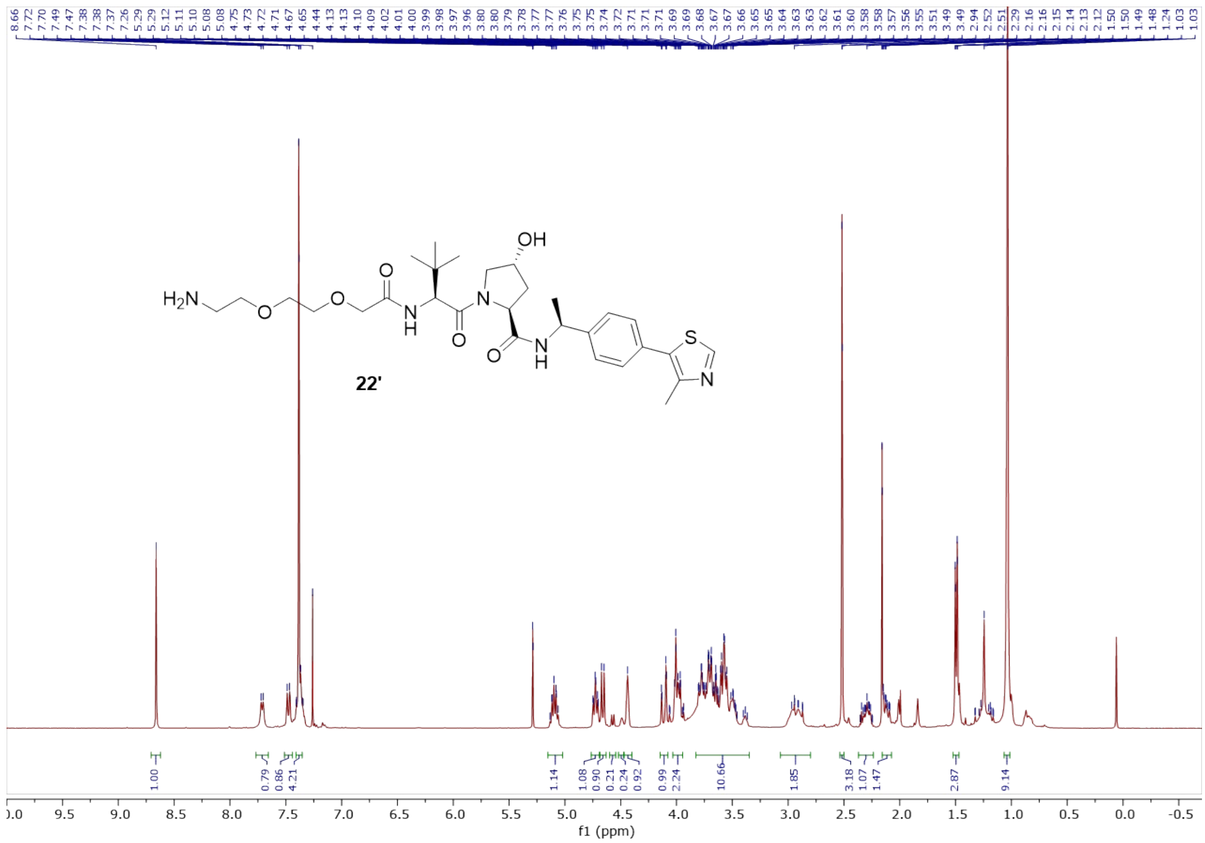
8.66 7.72 7.70 7.49 7.47 7.38 7.38 7.37 7.26 5.29 5.29 5.12 5.11 5.10 5.08 5.08 4.75 4.73 4.72 4.71 4.67 4.65 4.44 4.13 4.13 4.10 4.09 4.02 4.01 4.00 3.99 3.98 3.97 3.96 3.80 3.80 3.79 3.78 3.77 3.77 3.76 3.75 3.75 3.74 3.72 3.71 3.71 3.71 3.69 3.69 3.68 3.67 3.67 3.66 3.65 3.64 3.63 3.63 3.62 3.61 3.60 3.58 3.58 3.57 3.56 3.55 3.51 3.49 3.49 2.94 2.52 2.51 2.29 2.16 2.16 2.15 2.14 2.13 2.12 1.50 1.50 1.49 1.48 1.24 1.03 1.03
OH
H_2N
O
N
H
S
N
22'
1.00 0.79 0.86 4.21 1.14 1.08 0.90 0.21 0.24 0.92 0.99 2.24 10.66 1.85 3.18 1.07 1.47 2.87 9.14
10.0 9.5 9.0 8.5 8.0 7.5 7.0 6.5 6.0 5.5 5.0 4.5 4.0 3.5 3.0 2.5 2.0 1.5 1.0 0.5 0.0 -0.5
f1 (ppm)

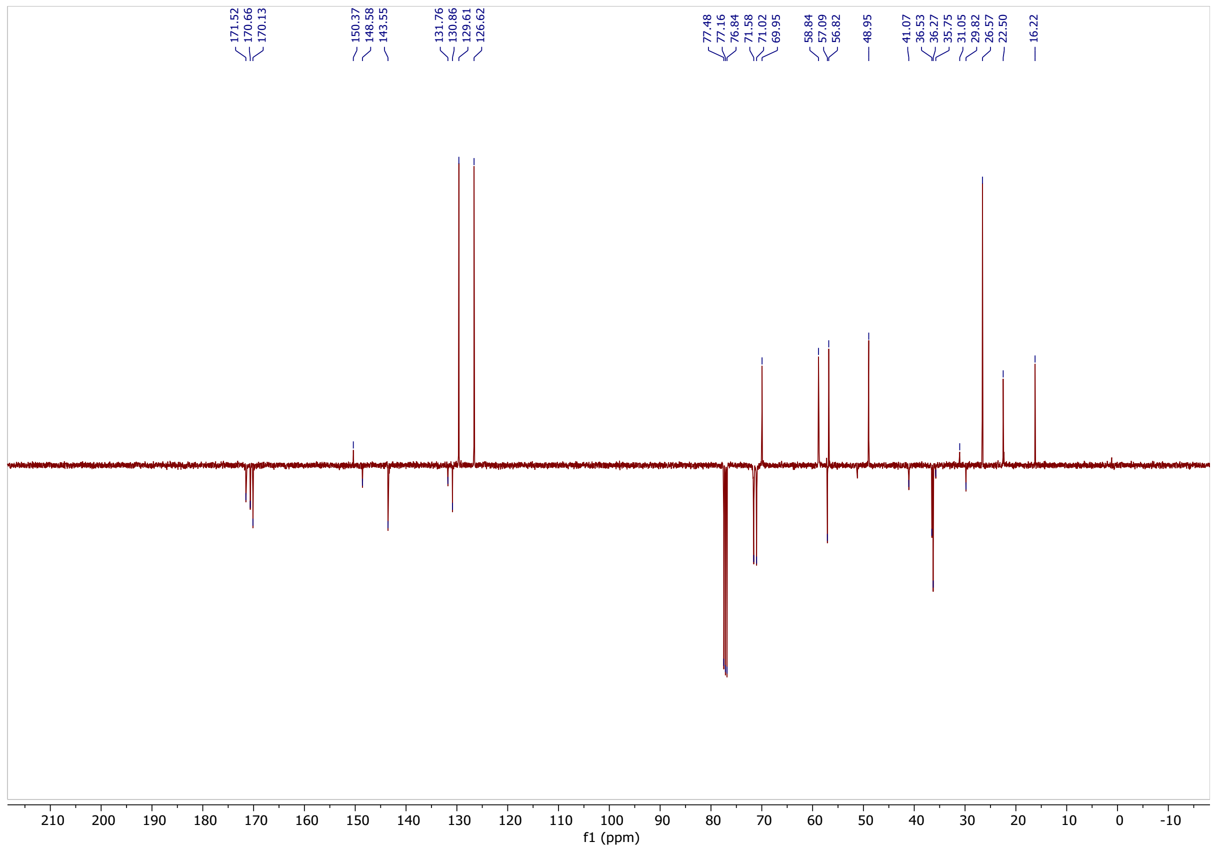
171.52 170.66 170.13 150.37 148.58 143.55 131.76 130.86 129.61 126.62 77.48 77.16 76.84 71.58 71.02 69.95 58.84 57.09 56.82 48.95 41.07 36.53 36.27 35.75 31.05 29.82 26.57 22.50 16.22
210 200 190 180 170 160 150 140 130 120 110 100 90 80 70 60 50 40 30 20 10 0 -10
f1 (ppm)

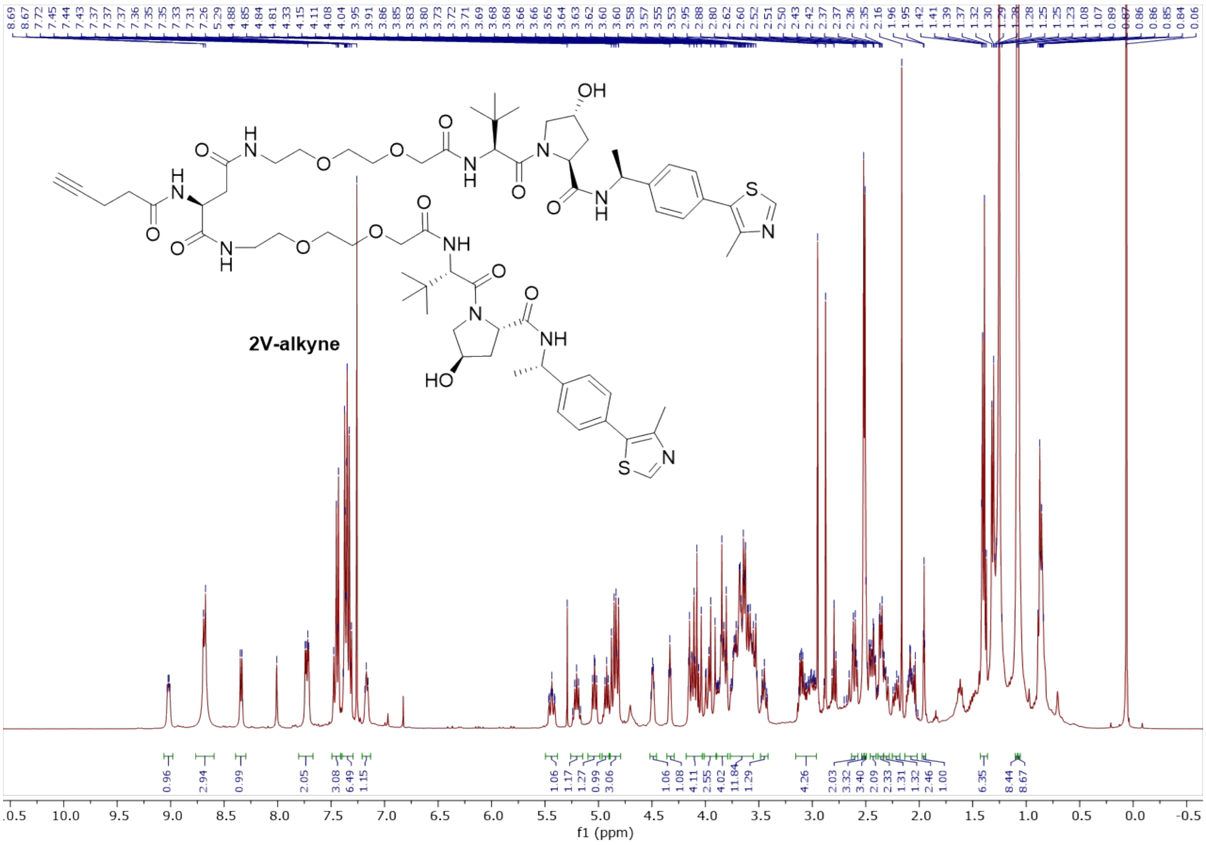
2V-alkyne
f1 (ppm)

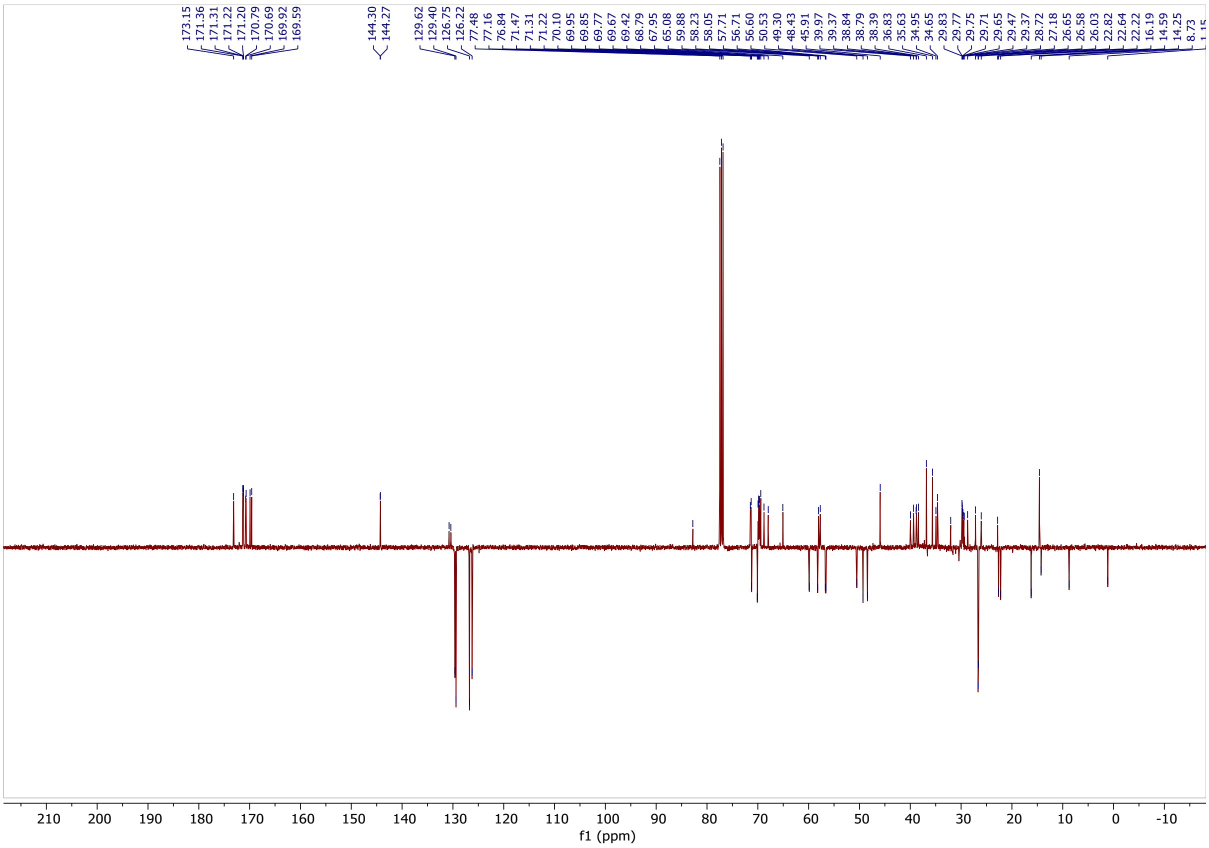
f1 (ppm)

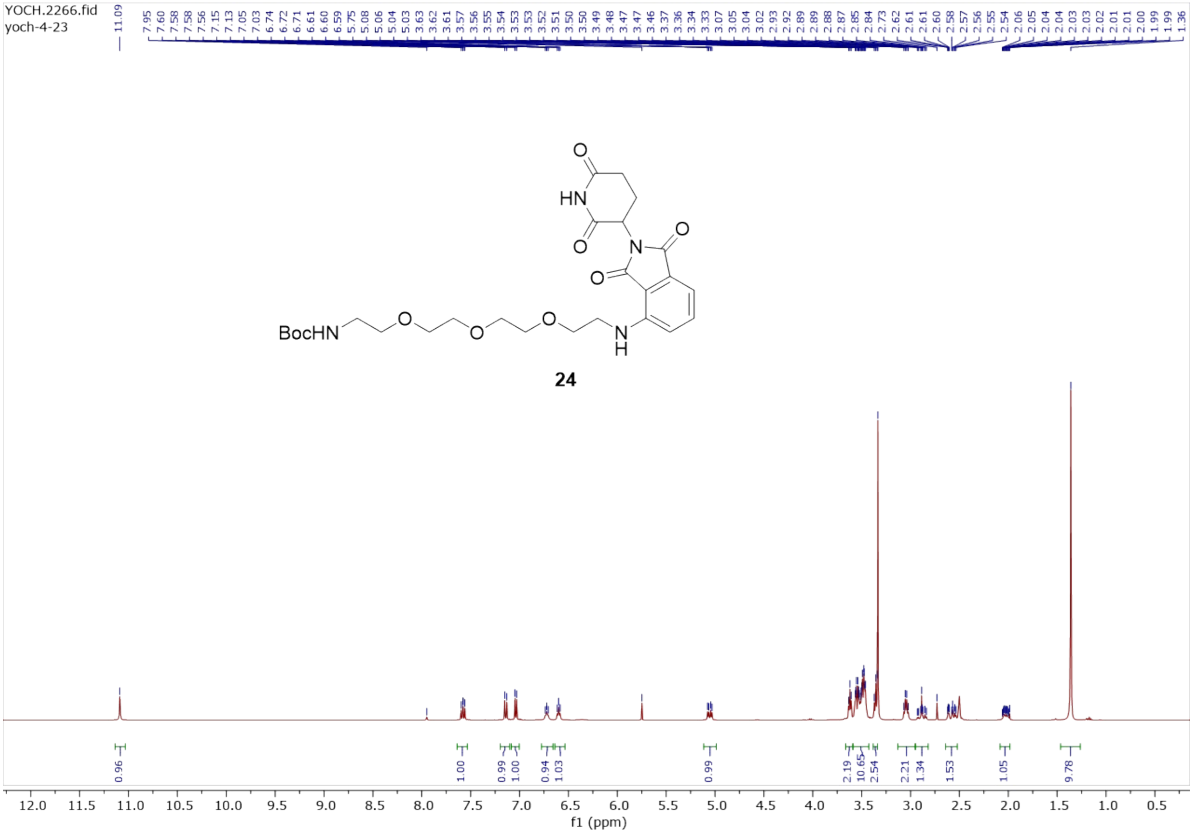
YOCH.2266.fid
yoch-4-23
11.09
7.95 7.60 7.58 7.58 7.56 7.15 7.13 7.05 7.03 6.74 6.72 6.71 6.61 6.60 6.59 5.75 5.08 5.06 5.04 5.03 3.63 3.62 3.61 3.57 3.56 3.55 3.54 3.53 3.53 3.52 3.51 3.50 3.50 3.49 3.48 3.47 3.47 3.46 3.37 3.36 3.34 3.33 3.07 3.05 3.04 3.02 2.93 2.92 2.89 2.89 2.88 2.87 2.85 2.84 2.73 2.62 2.61 2.61 2.60 2.58 2.57 2.56 2.55 2.54 2.06 2.05 2.04 2.04 2.03 2.03 2.02 2.01 2.01 2.00 1.99 1.99 1.36
BocHN
24
0.96
1.00
0.99
1.00
0.94
1.03
0.99
2.19
10.65
2.54
2.21
1.34
1.53
1.05
9.78
f1 (ppm)

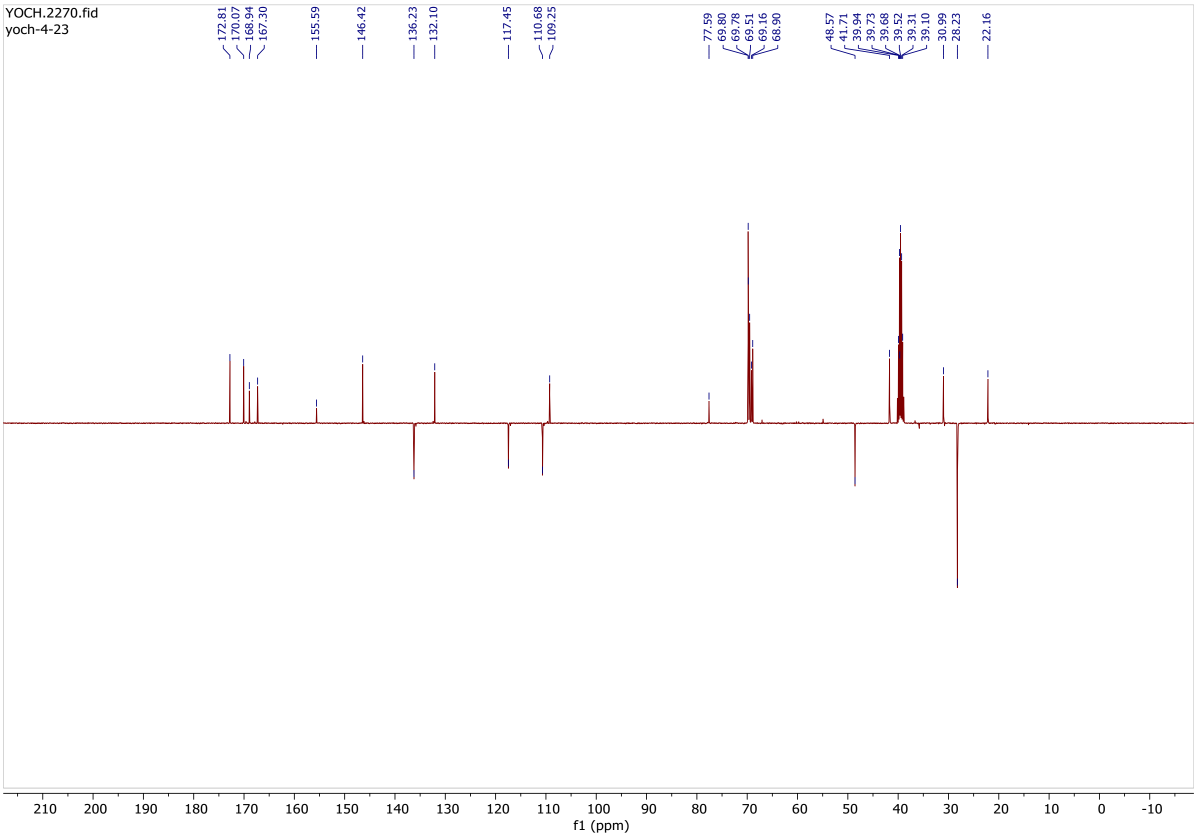
YOCH.2270.fid
yoch-4-23
172.81 170.07 168.94 167.30
155.59
146.42
136.23 132.10
117.45
110.68 109.25
77.59 69.80 69.78 69.51 69.16 68.90
48.57 41.71 39.94 39.73 39.68 39.52 39.31 39.10
30.99 28.23
22.16
f1 (ppm)

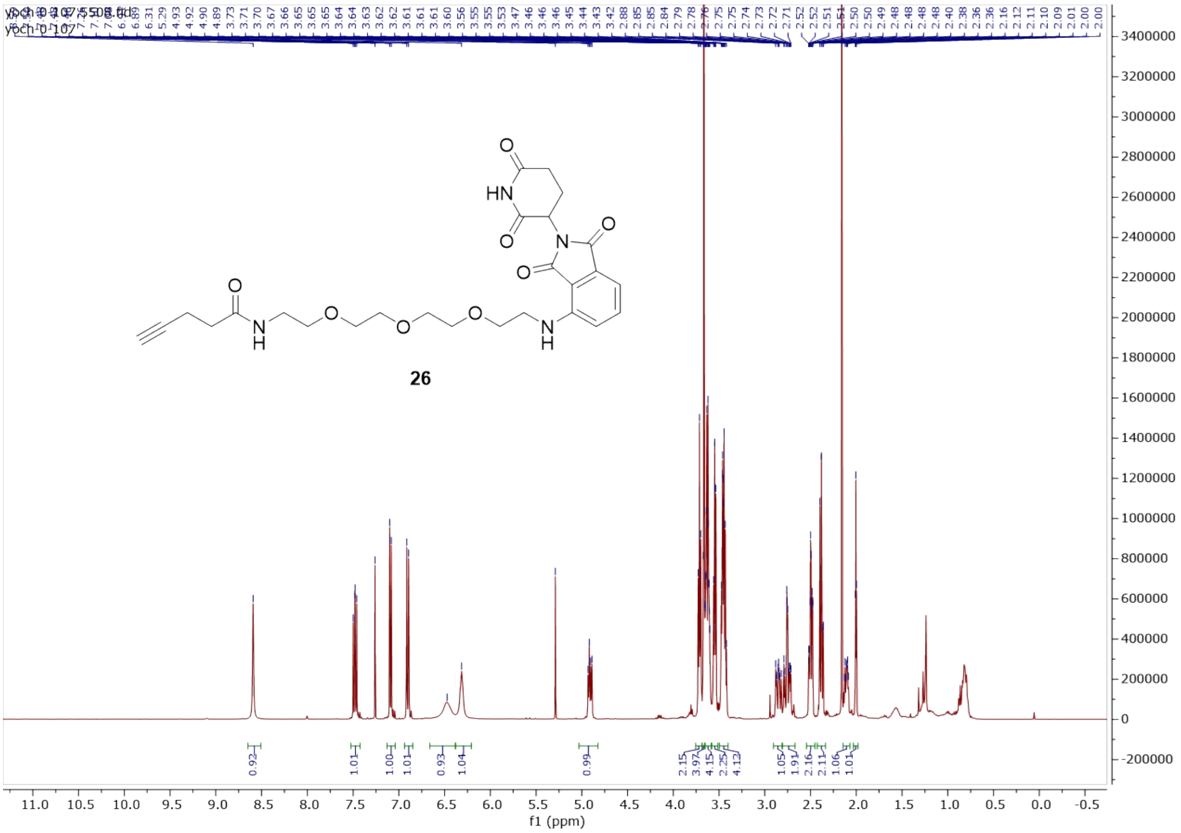
yoch-0-107
HN
O
N
H
26
0.92
1.01
1.00
1.01
0.93
1.04
0.99
2.15
3.97
4.15
2.25
4.12
1.05
1.91
2.16
2.11
1.06
1.01
f1 (ppm)

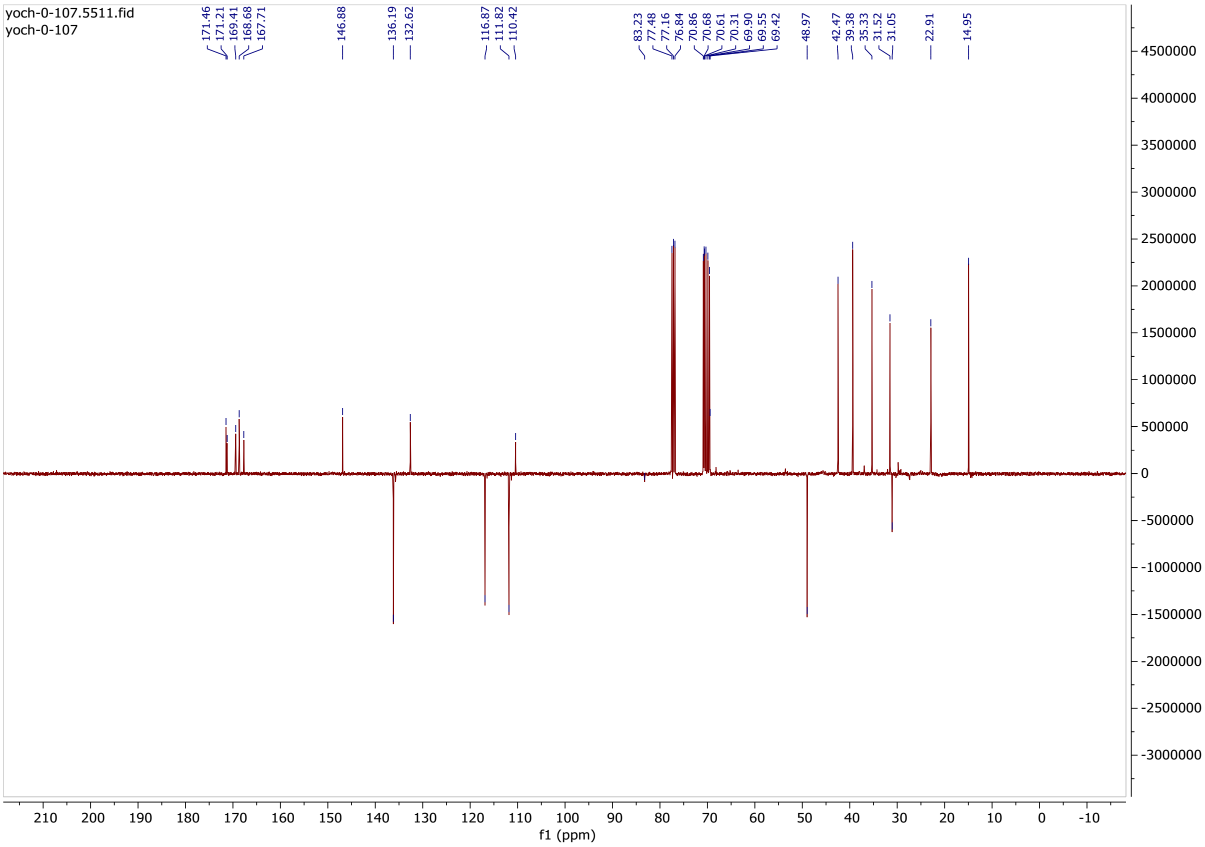
yoch-0-107.5511.fid
yoch-0-107
171.46
171.21
169.41
168.68
167.71
146.88
136.19
132.62
116.87
111.82
110.42
83.23
77.48
77.16
76.84
70.86
70.68
70.61
70.31
69.90
69.55
69.42
48.97
42.47
39.38
35.33
31.52
31.05
22.91
14.95
f1 (ppm)

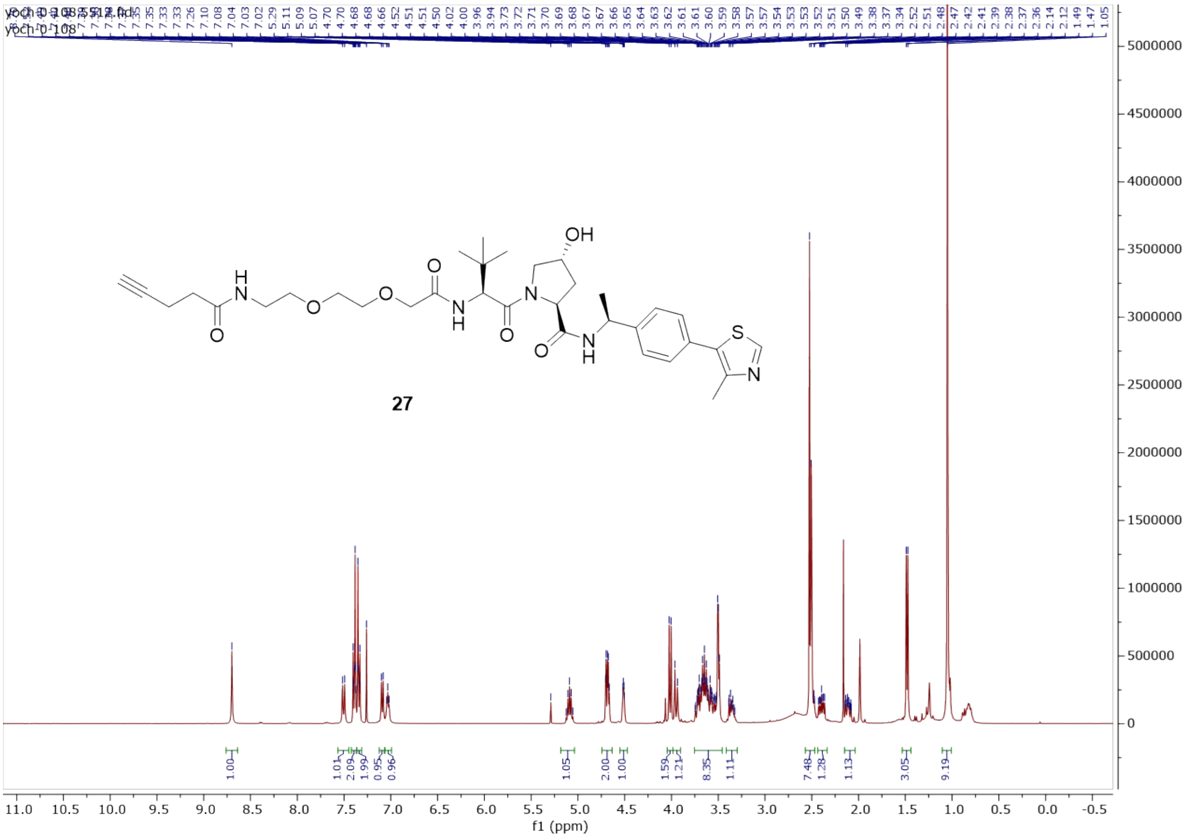

yoch-0-108
7.35 7.33 7.33 7.26 7.10 7.08 7.04 7.03 7.02 5.29 5.11 5.09 5.07 4.70 4.70 4.68 4.68 4.66 4.52 4.51 4.51 4.50 4.02 4.00 3.96 3.94 3.73 3.72 3.71 3.70 3.69 3.68 3.67 3.67 3.66 3.65 3.64 3.63 3.62 3.61 3.61 3.60 3.59 3.58 3.57 3.57 3.54 3.53 3.53 3.52 3.51 3.50 3.49 3.38 3.37 3.34 2.52 2.51 2.48 2.47 2.42 2.41 2.39 2.38 2.37 2.36 2.14 2.12 1.49 1.47 1.05
OH
H
N
O
S
N
27
1.00 1.01 2.09 1.99 0.95 0.96 1.05 2.00 1.00 1.59 1.21 8.35 1.11 7.48 1.28 1.13 3.05 9.19
f1 (ppm)

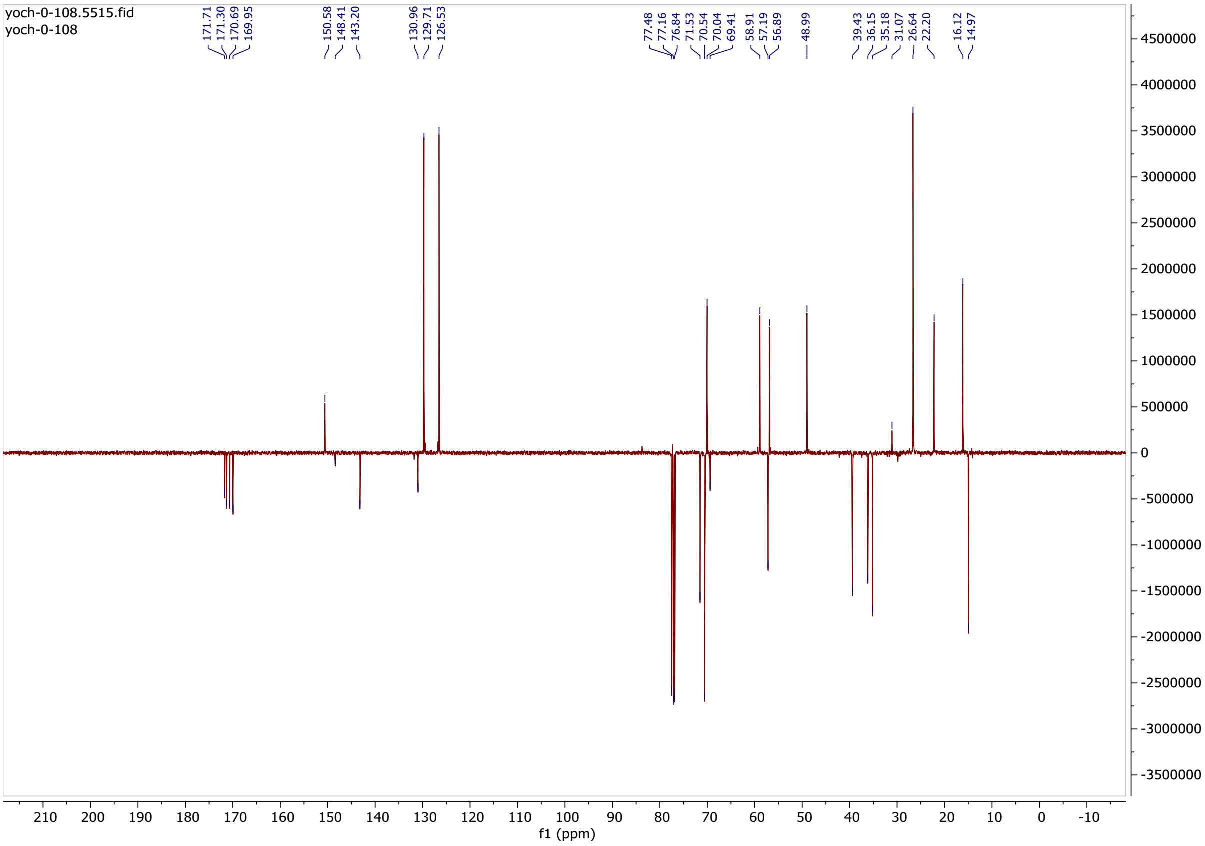

yoch-0-108.5515.fid
yoch-0-108
171.71 171.30 170.69 169.95 150.58 148.41 143.20 130.96 129.71 126.53 77.48 77.16 76.84 71.53 70.54 70.04 69.41 58.91 57.19 56.89 48.99 39.43 36.15 35.18 31.07 26.64 22.20 16.12 14.97
f1 (ppm)

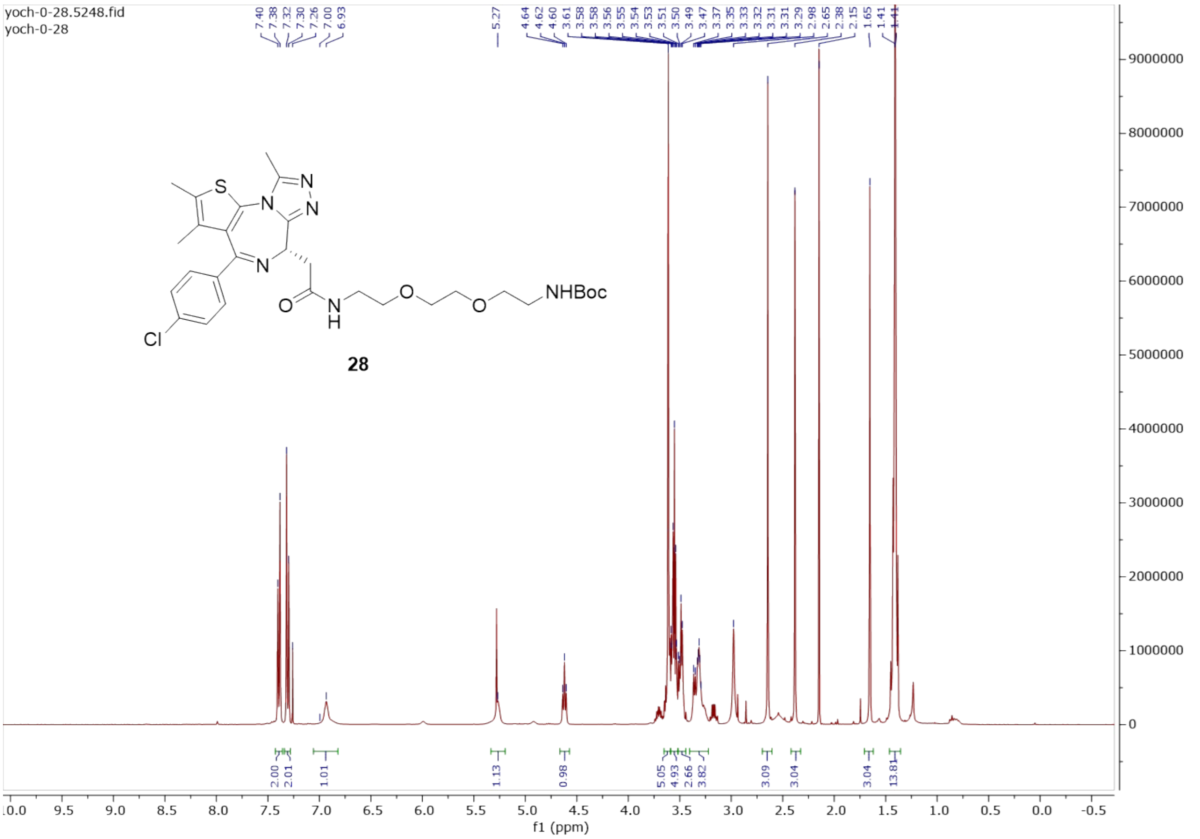
yoch-0-28.5248.fid
yoch-0-28
7.40 7.38 7.32 7.30 7.26 7.00 6.93
5.27
4.64 4.62 4.60 3.61 3.58 3.58 3.56 3.55 3.54 3.53 3.51 3.50 3.49 3.47 3.37 3.35 3.33 3.32 3.31 3.31 3.29 2.98 2.65 2.38 2.15 1.65 1.41 1.41
NHBoc
28
2.00 2.01 1.01 1.13 0.98 5.05 4.93 2.66 3.82 3.09 3.04 3.04 13.81
f1 (ppm)

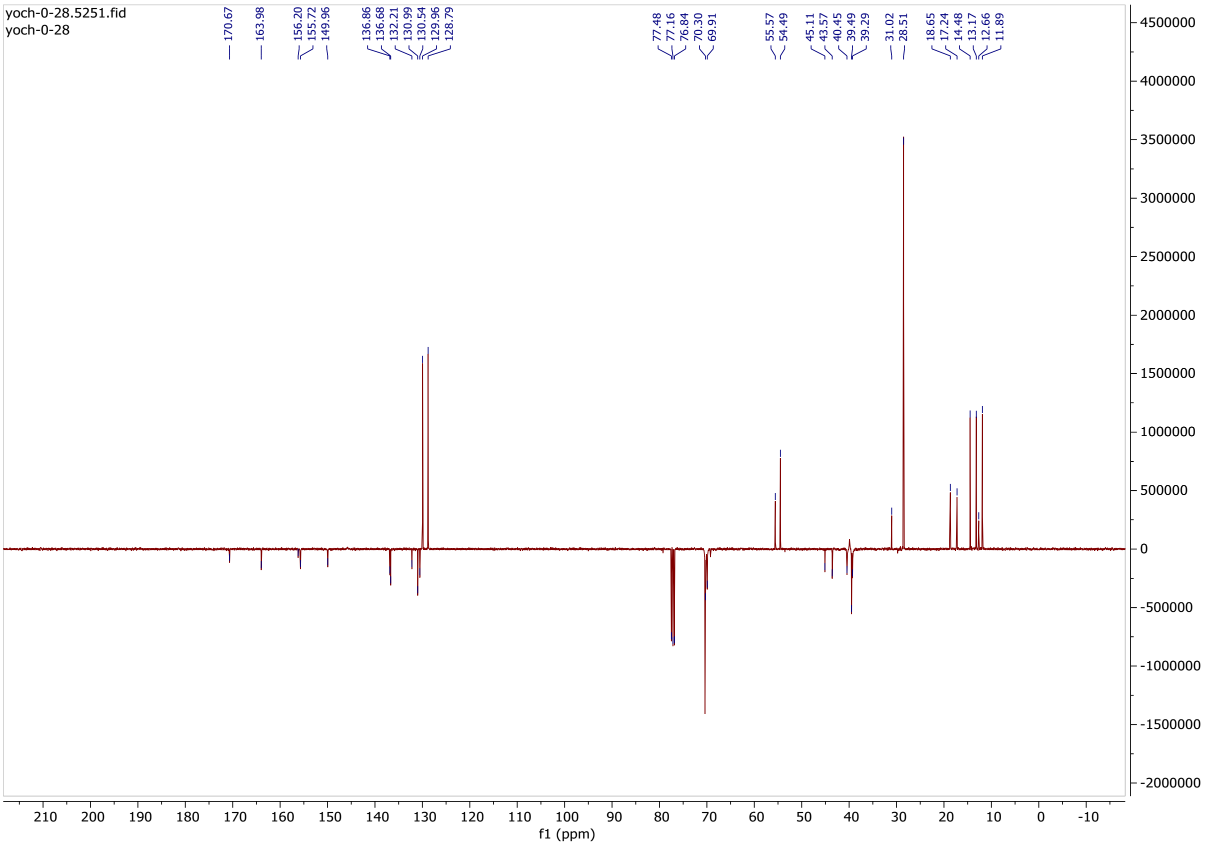
yoch-0-28.5251.fid
yoch-0-28
170.67 163.98 156.20 155.72 149.96 136.86 136.68 132.21 130.99 130.54 129.96 128.79
77.48 77.16 76.84 70.30 69.91
55.57 54.49 45.11 43.57 40.45 39.49 39.29 31.02 28.51 18.65 17.24 14.48 13.17 12.66 11.89
f1 (ppm)

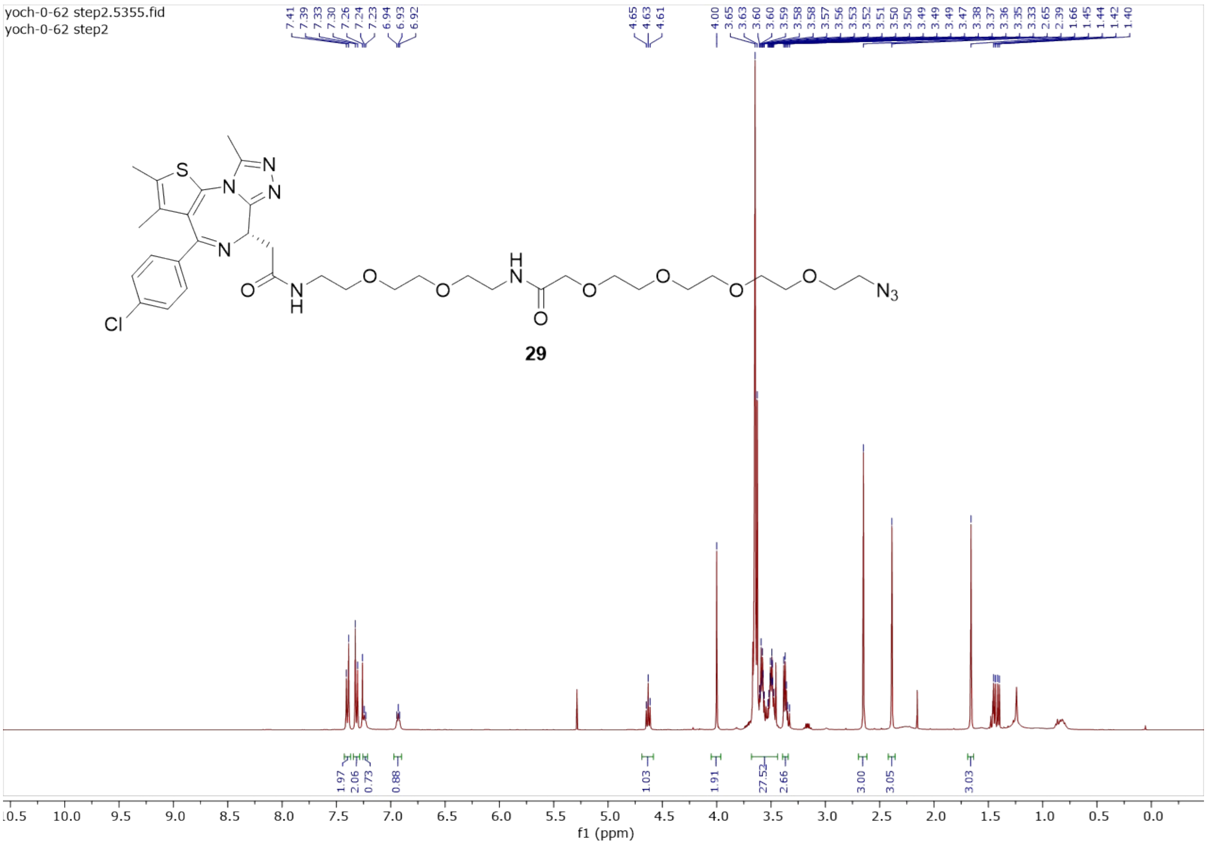
yoch-0-62 step2.5355.fid
yoch-0-62 step2
7.41 7.39 7.33 7.30 7.26 7.24 7.23 6.94 6.93 6.92
4.65 4.63 4.61
4.00 3.65 3.63 3.60 3.60 3.59 3.58 3.58 3.57 3.56 3.53 3.52 3.51 3.50 3.50 3.49 3.49 3.49 3.47 3.38 3.37 3.36 3.35 3.33 2.65 2.39 1.66 1.45 1.44 1.42 1.40
S
N
Cl
O
H
N_3
29
1.97 2.06 0.73 0.88 1.03 1.91 27.52 2.66 3.00 3.05 3.03
10.5 10.0 9.5 9.0 8.5 8.0 7.5 7.0 6.5 6.0 5.5 5.0 4.5 4.0 3.5 3.0 2.5 2.0 1.5 1.0 0.5 0.0
f1 (ppm)

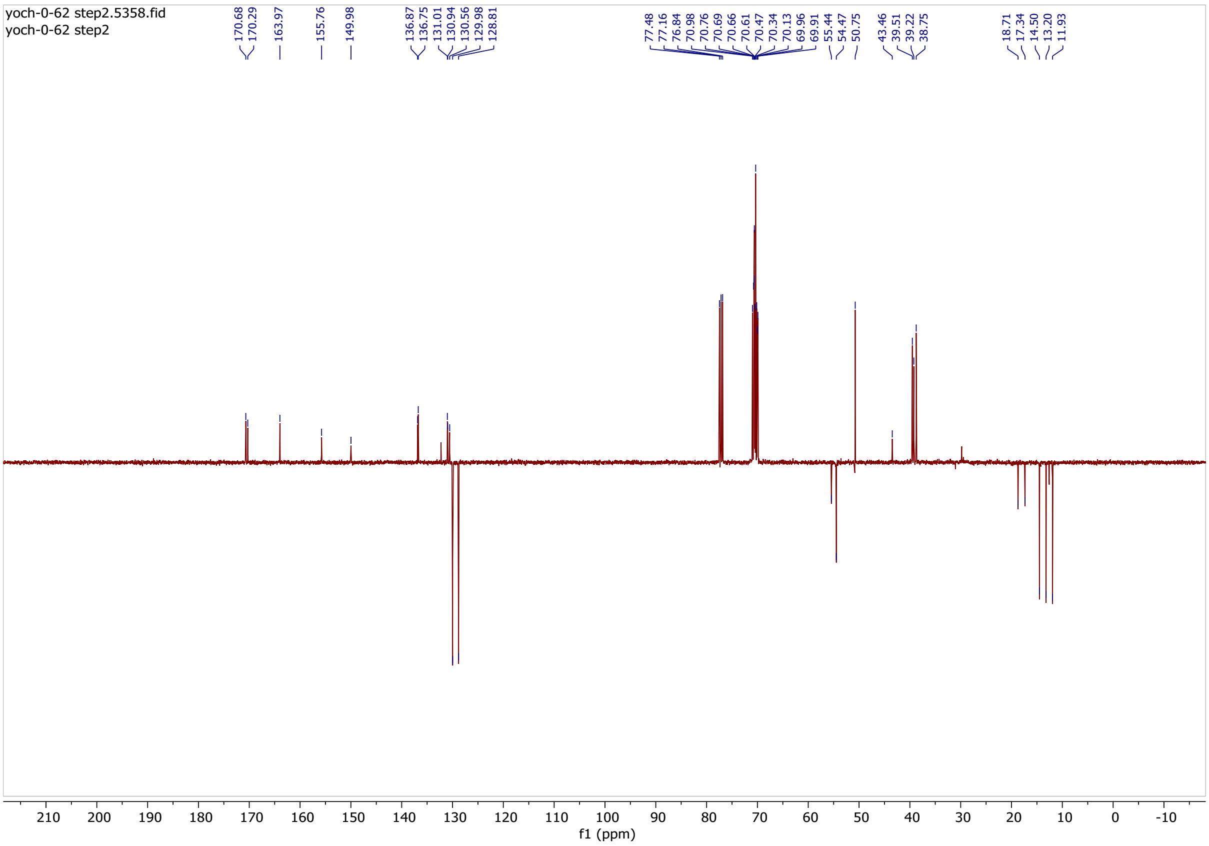
yoch-0-62 step2.5358.fid
yoch-0-62 step2
170.68 170.29 163.97 155.76 149.98 136.87 136.75 131.01 130.94 130.56 129.98 128.81
77.48 77.16 76.84 70.98 70.76 70.69 70.66 70.61 70.47 70.34 70.13 69.96 69.91 55.44 54.47 50.75 43.46 39.51 39.22 38.75
18.71 17.34 14.50 13.20 11.93
210 200 190 180 170 160 150 140 130 120 110 100 90 80 70 60 50 40 30 20 10 0 -10
f1 (ppm)
